# A simultaneous optical and electrical *in-vitro* neuronal recording system to evaluate microelectrode performance

**DOI:** 10.1371/journal.pone.0237709

**Published:** 2020-08-20

**Authors:** Zaid Aqrawe, Nitish Patel, Yukti Vyas, Mahima Bansal, Johanna Montgomery, Jadranka Travas-Sejdic, Darren Svirskis

**Affiliations:** 1 School of Pharmacy, The University of Auckland, Auckland, New Zealand; 2 Department of Electrical and Computer Engineering, The University of Auckland, Auckland, New Zealand; 3 Department of Physiology and Center for Brain Research, The University of Auckland, Auckland, New Zealand; 4 School of Chemical Sciences and MacDiarmid Institute for Advanced Materials and Nanotechnology, The University of Auckland, Auckland, New Zealand; University of Minnesota, UNITED STATES

## Abstract

**Objectives:**

In this paper, we aim to detail the setup of a high spatio-temporal resolution, electrical recording system utilising planar microelectrode arrays with simultaneous optical imaging suitable for evaluating microelectrode performance with a proposed ′performance factor′ metric.

**Methods:**

Techniques that would facilitate low noise electrical recordings were coupled with voltage sensitive dyes and neuronal activity was recorded both electrically via a customised amplification system and optically via a high speed CMOS camera. This technique was applied to characterise microelectrode recording performance of gold and poly(3,4-ethylenedioxythiophene)/polystyrene sulfonate (PEDOT/PSS) coated electrodes through traditional signal to noise (SNR) calculations as well as the proposed performance factor.

**Results:**

Neuronal activity was simultaneously recorded using both electrical and optical techniques and this activity was confirmed via tetrodotoxin application to inhibit action potential firing. PEDOT/PSS outperformed gold using both measurements, however, the performance factor metric estimated a 3 fold improvement in signal transduction when compared to gold, whereas SNR estimated an 8 fold improvement when compared to gold.

**Conclusion:**

The design and functionality of a system to record from neurons both electrically, through microelectrode arrays, and optically via voltage sensitive dyes was successfully achieved.

**Significance:**

The high spatiotemporal resolution of both electrical and optical methods will allow for an array of applications such as improved detection of subthreshold synaptic events, validation of spike sorting algorithms and a provides a robust evaluation of extracellular microelectrode performance.

## Introduction

The brain is one of the most intricate organs, functioning to control our physical senses, emotions and bodily processes. Specialised cells named neurons enable these complex functions through a network of connections and sophisticated electrochemical communication. The last decade has seen a surge of research which aims to interface these neural networks with electrode arrays in order to monitor and affect diseased pathways at an *in vitro* level through microelectrode arrays (MEAs) and an *in vivo* level through implantable electrode arrays [1]. The rationale behind this approach is that electrodes can correct or stimulate activity in certain neurons through a current pulse which causes depolarisation or hyperpolarisation of the cell [2]. Thus, achieving control over the diseased neural circuit.

At the front-line of these devices are electrodes which interface with the neuron to record or stimulate activity. They are typically made of noble metal materials, such as platinum, but the demand for smaller electrodes to achieve high spatiotemporal resolution has strained the performance of these materials through a subsequent increase in impedance [3]. Therefore, this space has seen the rise of an exhaustive list of electrode materials which aim to increase the electrochemically active surface area whilst maintaining the same desired geometric area. Materials which have received the most attention due to superior electrochemical performance, stability and biocompatibility include poly(3,4-ethylenedioxythiophene) (PEDOT) [4–8], carbon nanotubes (CNTs) [9–11], glassy carbon [12], iridium oxide (IrOx) [13, 14] and nanostructured platinum [13, 15].

Characterisation methods of these electrode materials are well established and comprise morphological, biological, electrochemical and cellular activity recording investigations [8]. Morphological tests are generally carried out through scanning electron microscopy (SEM) to elicit the microstructure of the electrode material. Electrochemical tests comprise three individual parameters being (i) charge storage capacity (CSC_*c*_) through cyclic voltammetry within the materials water window potentials, (ii) impedance through electrochemical impedance spectroscopy and (iii) charge injection limit through voltage transient measurements [16]. These electrochemical parameters predict favourable electrode properties for neuronal recording and stimulation. Biocompatibility assessment is carried out through the growth of cell cultures onto an MEA *in vitro* [8, 17, 18] and foreign body responses to implanted electrode arrays *in vivo* [10, 13, 19]. Recording and stimulation performance of the microelectrode are usually quantified through attribution of a signal to noise ratio (SNR) and the calculation of spikes arising from activated neurons in response to an injected current pulse. The SNR is an important metric and is generally calculated through (Eq 1)

$$\begin{array}{c} SNR\left(dB\right)=20*log_{10}\frac{S_{p-p}}{N_{p-p}} \end{array}$$ (1)

where S_*p*−*p*_ is the amplitude of signals attributed to neuronal spiking and N_*p*−*p*_ is the amplitude of electrical activity where no neuronal signals are recorded. The SNR is a generally accepted metric of electrode recording performance *in vitro* and *in vivo*. The impedance of a microelectrode is indirectly proportional to S_*p*−*p*_ and directly proportional to N_*p*−*p*_, therefore if impedance is lowered a higher SNR can be achieved—justifying the SNR metrics place in characterisation of neuronal microelectrodes. However, a large determinant of S_*p*−*p*_ is the distance of the signal source (neuron) from the microelectrode and this factor is not taken into account in the Eq 1. Therefore, if a cell is distant from the recording electrode the SNR may be reported falsely low for a specific electrode material.

We hypothesise that a characterisation method which takes into account the distance of the signal source would more accurately represent the performance of an electrode material to transduce neuronal signals. The proposed metric will be named ‘performance factor’ and will require locating the firing neuron optically alongside simultaneous electrical recording of its activity. To do this, we aim to construct an electrophysiology system which can record electrical spiking activity from a population of primary neuronal cells, and couple this with optical imaging of neuronal action potentials through voltage sensitive dyes (VSDs). VSDs are optical indicators sensitive to membrane potential. They offer the possibility to visualise, in real time, the electrical activity of large neuronal populations with high spatial (up to 0.5 *μ*m [20]) and temporal (*μ*seconds) resolution [21, 22], with the fastest VSDs based on electrochromic mechanisms (also known as the Stark effect) having less than 0.1 *μ*s response times. Chien and Pine confirmed the ability of VSDs to detect hyperpolarisation events, action potentials and subthreshold synaptic potentials with simultaneous patch electrode recording [23, 24]. Since then, improvements to VSD design have seen an increase in probe sensitivity from 1%/100 mV to 10%/100 mV fluorescence changes, leading to better detection of these electrophysiological events [20, 25].

In this paper, we present schematics for a low noise amplifier to acquire high quality electrical recordings from MEA devices as well as optimised VSD protocols and imaging techniques using a high speed CMOS camera. Primary hippocampal cells are cultured onto MEA devices which contain both bare gold and PEDOT coated microelectrodes to test the ′performance factor′ of different electrode materials. This metric is then compared to the conventional SNR calculation using the same materials. The constructed system is also validated for detection of neuronal signals from both visual and electrical sources through addition of tetrodotoxin (TTX), an irreversible sodium channel blocker. We believe that this methodology will more accurately characterise microelectrode performance, and provides a blueprint for further applications such as validation of spike sorting algorithms.

## Materials and methods

### Microelectrode array fabrication

A custom 14 channel microelectrode array to allow for extracellular neuronal recordings was constructed. Gold slides with a titanium adhesion layer (Au/Ti) were purchased from Deposition Research Laboratories Incorporated (DRLI) and patterned using conventional photolithographic protocols. nLOF 2070 was used as a mask to allow for selective etching of Au and Ti layers, SU-8 2005 was then used as an overlying insulation layer—exposing 20 *μ*m diameter areas of gold on each of the electrodes. Each microelectrode could be individually addressed through header pins which were soldered onto the MEA at a distant site.

#### Microelectrode modification with conducting polymer

Conducting polymer (CP) modification was undertaken to increase electrochemical surface area, decrease impedance and theoretically improve microelectrode recording performance. Poly(3,4-ethylenedioxythiophene) doped with polystyrene sulfonate (PEDOT/PSS) was electrochemically polymerised onto gold microelectrodes from a solution of 0.01 M 3,4-ethylenedioxythiophene (Sigma, 483028) and 0.1 M poly(sodium 4-styrene sulfonate) (Sigma, 243051). Polymerisation was carried out in a three electrode cell consisting of a working electrode (gold microelectrode), silver/silver chloride (Ag/AgCl) reference electrode and gold counter electrode. Galvanostatic polymerisation (constant current) was employed with a current density of 2 mA cm^−2^ until 1000 nC of charge was passed (318 mC cm^−2^). This was carried out using the Biologic VSP-300 electrochemical workstation.

#### Impedance characterisation

Microelectrode viability and electrochemical properties were assessed through electrochemical impedance spectroscopy (EIS). A three electrode set up was employed, consisting of an Ag/AgCl reference electrode, gold counter electrode and the microelectrode to be tested as the working electrode. A 10 mV sinusoidal wave was applied (Biologic VSP-300 electrochemical workstation) from 1 Hz to 10 kHz at open circuit potential in artificial cerebrospinal fluid (ACSF) to mimic impedance properties during recording.

### MEA acquisition system

#### MEA to amplifier interface

Microelectrodes on the MEA were connected to the amplifier input using an interface board. The board plugged into the MEA header pins and sat over the MEA slide with a window allowing the microscope objective lens to access the solution. Outputs from the board consisted of 14x 2-pin headers, with each pair consisting of a pin tied to a microelectrode on the MEA (connected to non-inverting input at amplifier) and the other to a common Ag/AgCl reference immersed in solution (connected to inverting input). The board also had a ground plane which was connected to the amplifier ground to reduce noise in the measurements.

#### Amplifier

The amplifier circuit (S1 File) was designed to be stable, low noise and low cost (US$21 for the first channel + US$11 for each additional channel). Full characterisation of this system can be found at [26]. Power circuitry consisted of a 9 V battery source (negative terminal connected to ground) with an ultra-low noise, linear regulator (LT3042, Linear Technology) used to maintain supply voltage to the circuit at +5 V. A reference voltage (REF) was set at 2.5 V by REF5025 (Texas Instruments), which provided a low noise, low drift reference potential. Two stages of amplification were used, the first stage being an instrumentation amplifier (LT1167, Linear Technology), and the second, an operational amplifier (LT1678, Linear Technology). Inverting and non-inverting input signals were AC coupled using with a high pass cut off frequency set at 0.7 Hz. To prevent voltage bias drifts at the input through capacitive charging, discharge paths were utilised using resistors. A LT1167 instrumentation amplifier was chosen at the first stage of amplification due to its low noise operation, high common mode rejection ratio (140 dB at a gain of 1000), low input bias current and high input impedance (200 GΩ) which allows the use of high impedance sources without additional offset voltage errors [27]. The high common mode rejection ratio (CMRR) ensures that the desired differential signal is amplified and unwanted common mode signals are attenuated. A single resistor sets the gain for the instrumentation amplifier at 1000. The common mode voltage is removed from the original signal by the instrumentation amplifier and results in a single-ended output voltage referenced to the voltage on the REF pin (2.5 V). The output voltage from LT1167 is high pass filtered with a cut off frequency set at 15.9 Hz and connected to the non-inverting input of LT1678 with reference to 2.5 V. Gain at LT1167 was set to 2 with a non-inverting feedback loop.

#### Analog to digital converter

Amplified signals were digitised using a data acquisition (DAQ) device from National Instruments (USB-6356). Samples were acquired simultaneously at 20 kHz per channel with 16 bit resolution. A custom built LabVIEW programme (S2 File) was used to interface with the DAQ and acquire/log data which was saved in .*tdms* format for later processing.

### Cell culture

Primary hippocampal neurons were obtained from P0 Wister rat pups under the University of Auckland’ Animal Ethics Committee approval (AEC numbers 1504 & 2051). The devices were immersed in deionised (MilliQ, 18.2 MΩ.cm) water for a minimum of 24 hours prior to culture to promote neuronal growth in order to remove aqueous contaminants left on the surface of the MEA devices during microfabrication and polymerisation processes. Primary hippocampal neurons from Wistar rats were cultured at postnatal day zero using established techniques [28, 29] onto the MEA devices. The MEA devices were first sterilised with 100% ethanol and exposure to UV light then coated with 10 *μ*g mL^−1^ poly-D-lysine (PDL, Sigma P1499) and left overnight at 37°C. Wistar pups were decapitated and hippocampi removed and placed in sterilised ice cold Hanks’ Balanced Salt solution (HBSS, Sigma H2387) buffer. The hippocampal neurons were enzymatically dissociated with papain (Worthington Biochemicals LK003178) in 5 mL HBSS and incubated at 37°C for 15 minutes. Enzyme inactivation solution (4.5 mL Minimum Essential Medium [Gibco 11090-081] + 0.5 mL Fetal Bovine Serum [Gibco 10091-148]) was added to the neurons after removal of papain to stop the dissociation process. Neurons were then titrated with Neural Basal Media (NBM, NBM: Gibco 21103-049, B27 supplement: Gibco 17504-044, and 1:100 GlutaMAX supplement [200mM L-glutamine in 0.85% NaCl, Invitrogen 35050-061]) until a homogenous mixture was formed. The resulting suspension was plated onto the MEA device at a density of one hippocampi per MEA setup. The MEA devices were then placed in a 5% CO2 incubator at 37°C for 28 days. Half the NBM was replaced at *days in vitro* 1 (DIV 1) and a quarter replaced at DIV 7, 14 and 21. Electrophysiological recording of neurons through the MEA was carried out in ACSF following VSD staining (detailed below).

### Optical acquisition system

#### VSD staining protocol

Pyridinium, 4-(2-(6-(dibutylamino)-2-naphthalenyl)ethenyl)-1-(3-sulfopropyl)-, hydroxide (Di-4-ANEPPS, Molecular Probes D1199) was used as the VSD to label primary hippocampal cells due to its consistant potentiometric response [30]. The dye solution was made from a 1:1 mix of 2 mM Di-4-ANEPPS in dimethyl sulfoxide (DMSO, Sigma D4540-500ML) and 2% pluronic F-127 (Sigma P2443-250G) in DMSO. DMSO was used as a diluent in line with the manufacturers recommendations. 10 *μ*L of the mixture was added to the cell culture media (4 mL) resulting in a final concentration of 2 *μ*M Di-4-ANEPPS and 0.02% pluronic F-127. The cells were incubated with the dye solution for 20 minutes at 37°C, followed by washing with dye free culture media.

#### Image acquisition

A high speed CMOS camera (MotionPro X3, IDT) was fitted onto a fluorescent microscope (Leica DM RXA2) consisting of a 50 W mercury light source (Leica), an I3 filter cube and a x10 water immersion fluorescent lens (Leica 10X/0.3 HCX APO). Excitation/emission maxima for Di-4-ANEPPS were approximately 465/635 nm (as measured in model membranes), respectively—I3 filter cube was characterised by an excitation filter at 450-490 nm and a long pass emission filter for wavelengths over 515 nm, making it suitable for use with Di-4-ANEPPS. Note that results could be further optimised by using filters provided by the manufacturer of the VSD as spectra may be shifted in live cell experiments by up to 100 nm. The high speed camera was set at an acquisition rate of 1000 Hz with an exposure time of 958 *μ*s (single exposure). Image acquisition and shutter position (open or closed) on the microscope were triggered by a digital signal from the DAQ to synchronise visual and electrical acquisition start times. Fluorescence intensity within the optical images was quantified using the Time Series Analyzer (V3) plug-in within ImageJ. A region of interest (ROI) around the visibly firing neuron was first selected, then the average fluorescence intensity was calculated within this ROI across the recorded frames. The ROI was saved and applied to subsequent data sets which contained the same neuron to ensure consistency within the analysis.

## Results & discussion

### MEA fabrication

**Fig 1:**
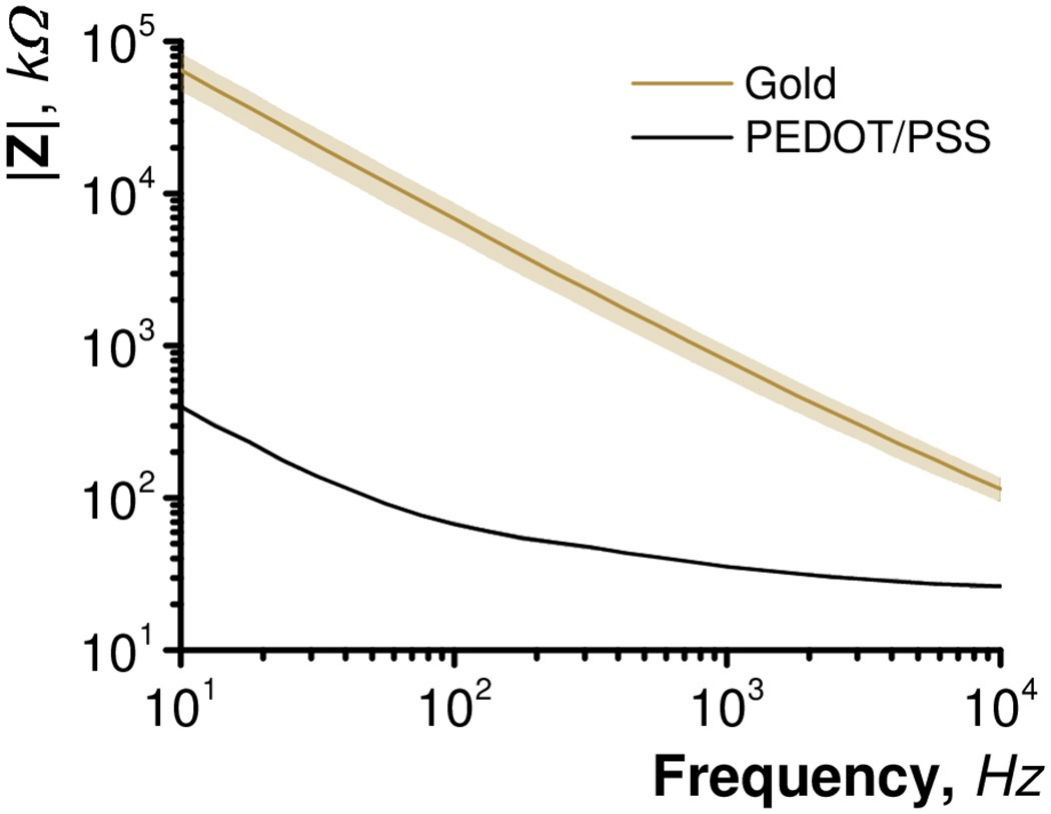
Impedance magnitude of uncoated gold and PEDOT/PSS modified microelectrodes in artificial cerebrospinal fluid (n = 3).

MEAs were successfully fabricated and uncoated gold microelectrodes displayed typical impedance spectra with a constant slope of -0.8 from the |*Z*| vs frequency bode plot (Fig 1), indicating capacitive impedance. This is consistent with previous reports of uncoated gold microelectrodes due to their capacitive mechanism of charge transfer at the electrode/electrolyte interface [31]. Electro-polymerisation of PEDOT/PSS onto the gold surface was accompanied by a drastic reduction of |*Z*| at all frequencies in the impedance spectra. This is commonly observed and can be attributed to the large electrochemical surface area offered by PEDOT/PSS [4, 32]. Electrodes intended for extracellular neuronal recordings are often characterised by their impedance magnitude at 1 kHz—a value close to the frequency of neuronal signals. In this case, PEDOT/PSS is dominated by its access resistance at 1 kHz therefore a comparison at a frequency of 100 Hz is more descriptive of the difference between the two electrodes. PEDOT/PSS produced a substantial drop in |*Z*|_100*Hz*_ from 4305 ± 342 kΩ to 67 ± 2 kΩ. Lower impedance magnitude is favourable for recording due a smaller noise floor and improved charge transfer properties [16, 33].

### MEA acquisition system

The amplifier boards were assembled into an aluminum case to shield the inputs from electromagnetic noise, preventing saturation between the amplifier supply rails. Three-core wires, consisting of two insulated wires and a shield, were used between the MEA interface board and amplifier input. Although these wires successfully reduced noise at the inputs, it was found that further reduction could be achieved by tying the cable shielding to the 2.5 V reference. The final intrinsic output noise of the amplifier was calculated to be 6.92 *μ*V_*peak*−*peak*_ (inverting and non-inverting input shorted).

**Fig 2:**
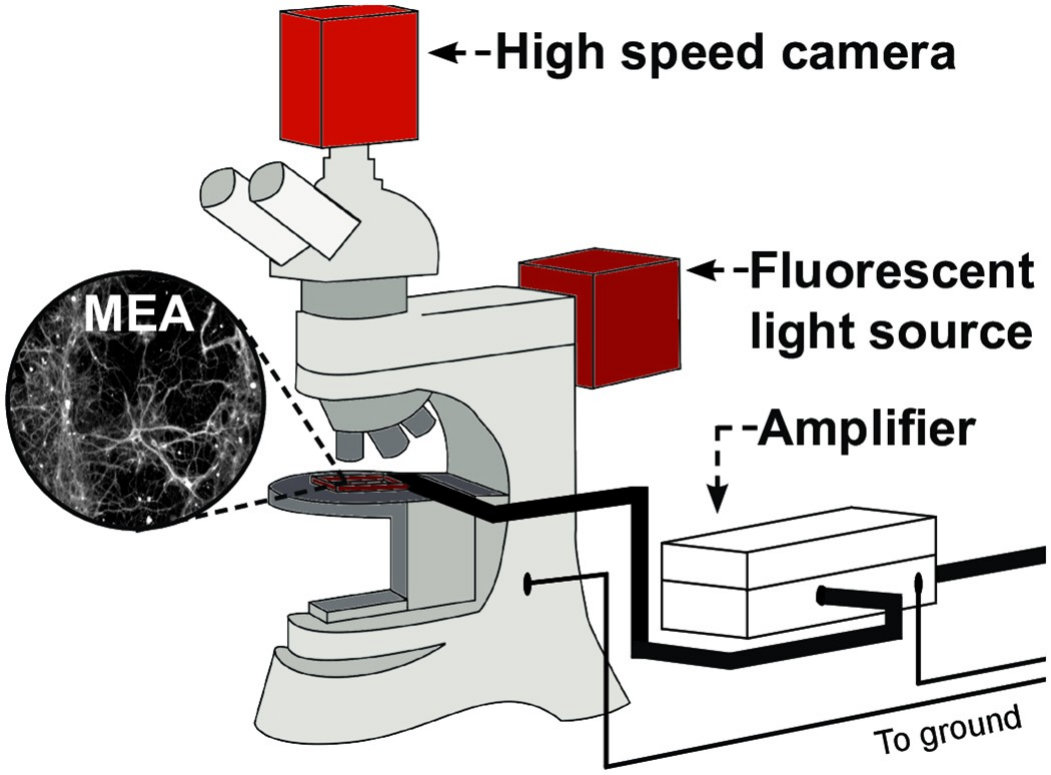
Illustration of the set-up used to obtain simulatanous electrical and optical signals from primary hippocampal cells cultured onto MEA devices. The MEA device sat on the microscope stage and shielded wires were connected to each of the electrodes through an interface board to enable electrical recording. The camera and mercury light source (highlighted in red) were mounted onto the microscope to enable optical recordings.

Peak to peak noise values rose to 38.2 ± 4.4*μ*V or 19 ± 2.2*μ*V when amplifier inputs were connected to gold or PEDOT/PSS microelectrodes, respectively. (average ± SD (n = 7)). This can be explained by high electrode impedance values, with gold having significantly higher values than PEDOT/PSS [16, 33]. Insertion of the objective lens into the media bathing the electrodes caused the amplifier to saturate with noise. This was likely due to noise being introduced into the system by the microscope and was resolved by connecting a metal portion of the microscope chassis to amplifier ground (Fig 2).

### Cell culture

**Fig 3:**
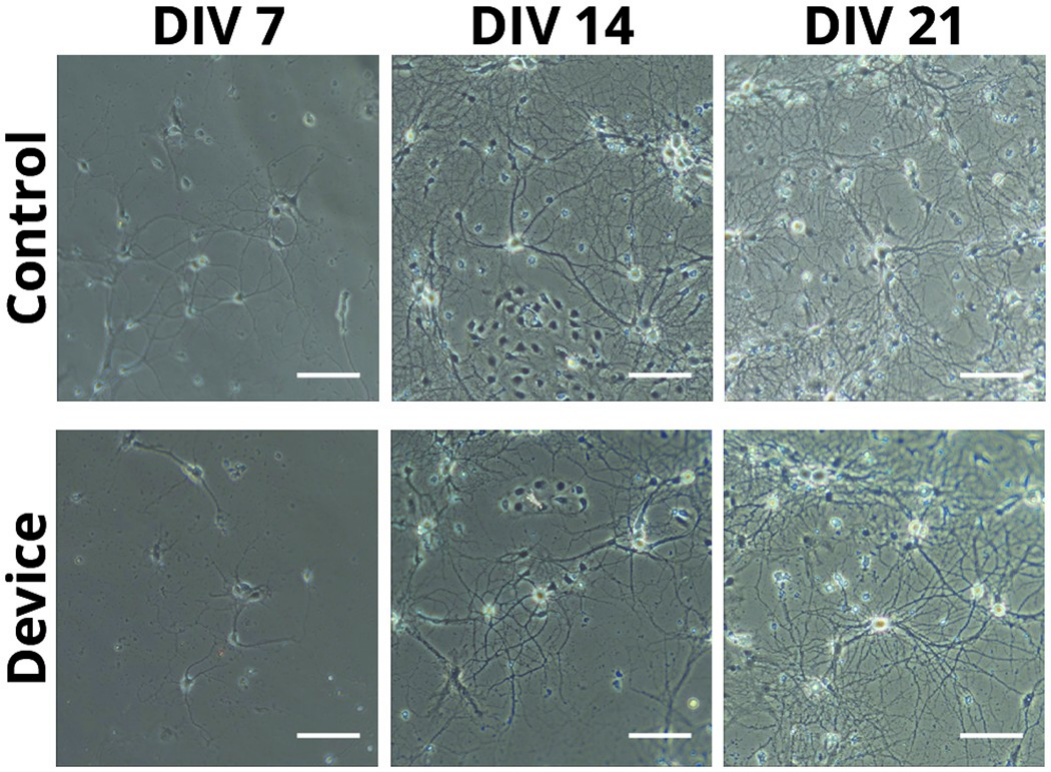
Primary hippocampal cells cultured onto control cover slips or MEA devices at DIV 7, 14 and 21. The images were taken using differential interference contrast microscopy through a light microscope equipped with a 20x water immersion objective lens. Scale bar represents 100 *μ*m.

Primary hippocampal neurons were cultured onto the MEA devices and displayed growth comparable to control cover slips routinely used in cell culture protocols. A time lapse of neuronal growth was achieved by taking photos at DIV 7, 14 and 21 to assess health and density (Fig 3). It can be seen that growth and connection density between neurons are consistent between the two samples.

### Simultaneous electrical and optical recording

**Fig 4:**
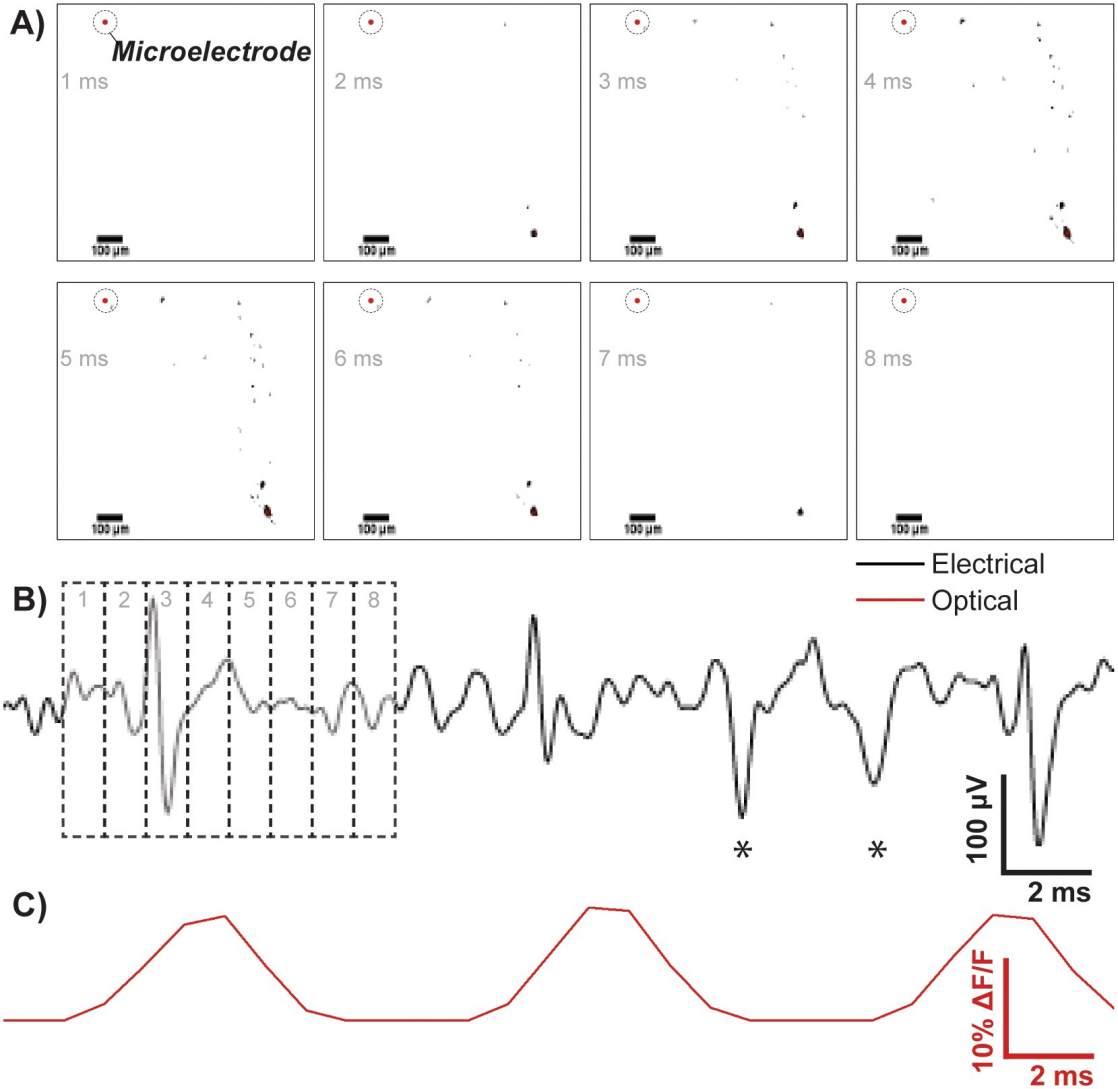
An example of combined electrical and optical recordings from a single electrode (indicated by a red encircled dot). A) Processed images of a typical action potential imaged through the high-speed camera, each frame is 1 ms long and the neuron is represented by the black dots which appear in the image. The correlating electrical signal to each of the frames is shown in B) where the numbers 1,2,3,4,5,6,7,8 correlate with the frame image at 1 ms, 2 ms, 3 ms, 4 ms, 5 ms, 6 ms, 7 ms and 8 ms in A. The measured change in fluorescence intensity from the acquired images is displayed in C) and this trace correlates in time with the electrical recording trace above it. The stars (⋆) highlight action potentials which were recorded electrically but not optically, possibly arising from another source within the culture.

Fig 4 demonstrates the successful integration of both optical and electrical recording systems. The 8 images within Fig 4A show the progression of an action potential as visualised by the VSD. Initially neurons were incubated with 1 *μ*M Di-4-ANEPPS, however signal strength was poor and individual cells were difficult to visualise on-screen. Increasing the incubation concentration to 2 *μ*M, resulted in clearer images with visible neuronal processes extending from the cell body. This was still within the manufacturers recommended loading concentration of 0.2—2 *μ*M. Increases in the excitation intensity or exposure time to improve signal quality were avoided to reduce the occurrence of phototoxicity and maintain temporal resolution, respectively. It should also be noted that while this study employed Di-4-ANEPPS, newer generation VSDs, such as di-2-ANEPEQ, di-3-ANEPPDHQ and di-4-AN(F)EPPTEA may have resulted in improved imaging [34]. Camera acquisition settings were adjusted to maximise the quality of digitised fluorescent signals. To visualise electrical activity high temporal resolution (<2 ms) and spatial resolution (<5 *μ*m) was required. The final settings utilised an acquisition rate of 1000 Hz with an exposure time of 958 *μ*s meaning a photo was taken every millisecond. The camera sensor gain was increased (x2) to accommodate for the decrease in signal strength due to low exposure times and 2x2 image binning was applied to further improve signal quality. Further binning at 3x3 and 4x4 did not provide any additional benefit and resulted in unnecessary loss of spatial resolution.

The representative electrical recording trace shown is derived from one PEDOT/PSS microelectrode which is indicated on the images in Fig 4 as a red encircled dot. The recording was band-pass filtered between 200 Hz and 2000 Hz using a 4^*th*^ order butterworth IIR filter to isolate neuronal spike data. The spikes were easily distinguishable from the noise floor making action potential identification simple. A digital cue to initiate acquisition resulted in synchronised activity for both electrical and optical acquisition methods—with overlapping activity in both methods seen clearly from Fig 4A, 4B & 4C. Spikes marked with a star (⋆) were identified electrically through the microelectrode but not optically. This could be due to a different neuron firing at a distant location, not within the cameras field of view. This raises an issue as a large field of view is needed to make full use of VSD within this method. A way to remedy this problem is to decrease the objective lens magnification or adapt the set-up so that it can be used with a fluorescent macroscope capable of viewing the entire slide [35]. It should be noted that microelectrodes on MEAs are typically separated by a distance of around 25 to 50 *μ*m, unlike the MEA presented here, therefore more than one electrode would typically be visible within the cameras field of view.

**Fig 5:**
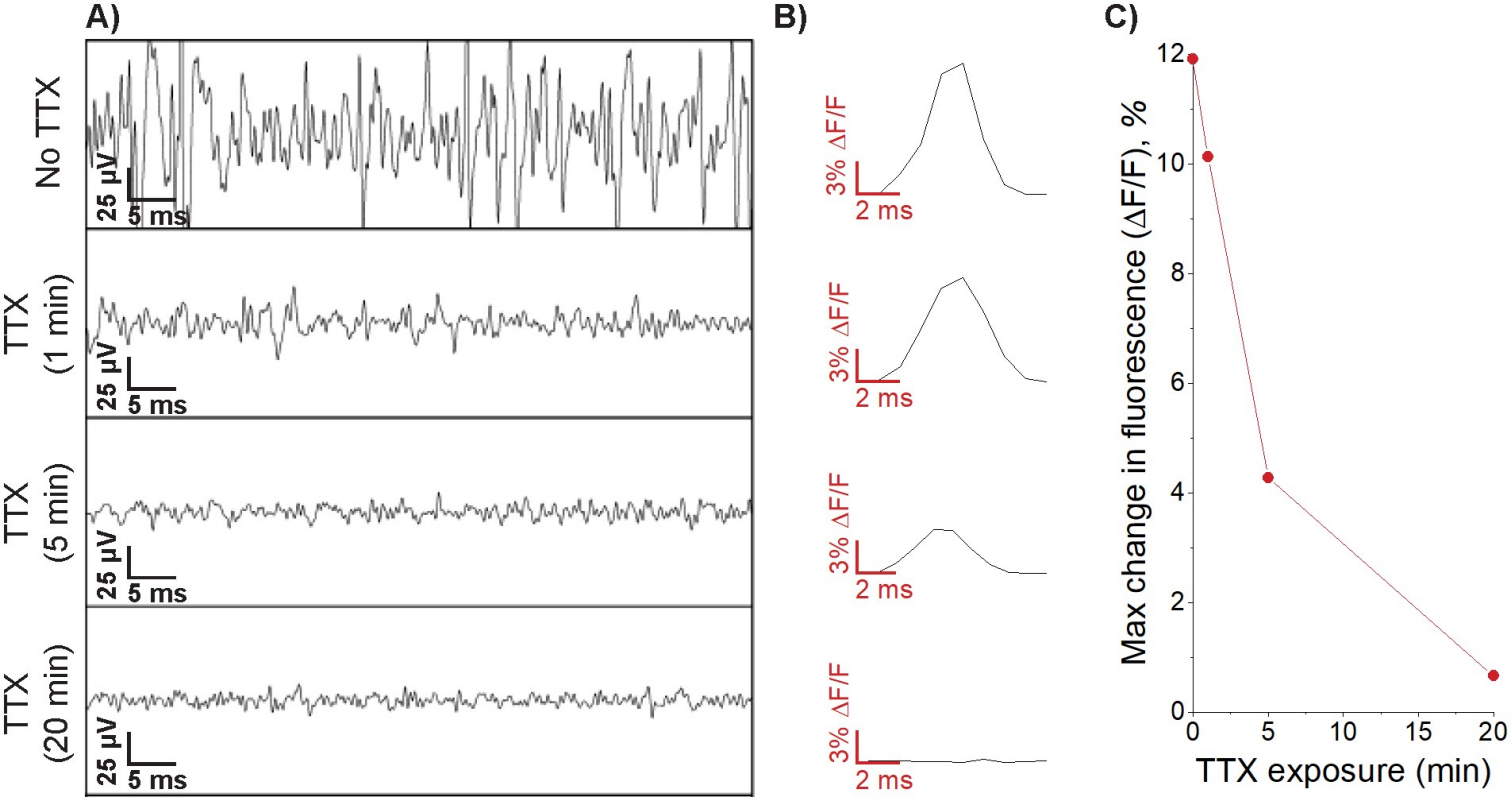
Electrical and optical signals were confirmed as neuronal action potentials due to antagonism following tetrodotoxin (TTX) addition. A) Representative electrical waveforms before TTX addition and 1 min, 5 min and 20 min post TTX addition, a clear reduction in electrical activity is shown. B) Representative change in fluorescence intensity waveforms for a single neuronal action potential prior to TTX addition and 1 min, 5 min and 20 min post TTX addition where a clear reduction in the waveform shape is observed. The ROI used to calculate fluorescence waveforms is identical to that used in Fig 4A to ensure consistency as the same neuron is being analysed. C) Demonstrates the relationship between the maximum change in fluorescence intensity and time following TTX exposure.

The recorded electrical signals were confirmed to originate from neurons through use of TTX, an inhibitor of neuronal action potentials through binding of voltage-gated sodium channels (Fig 5A). TTX (1 *μ*M) was added to the bath and recordings were taken at 1 minute, 5 minutes and 20 minutes to assess the effects of the toxin. The optical measurements displayed in Fig 5B indicate representative changes in fluorescence intensity waveforms of one action potential before and after TTX addition. After 1 minute a significant reduction in electrical activity was noticed, optical imaging appeared to suffer from a slight loss in fluorescence near the electrode. After 5 minutes, almost no action potentials could be sensed through the microelectrodes, and optical signals have started to show clear signs of diminishing activity. 20 minutes post TTX addition revealed diminished optical signals where only activity within the cell body could be sensed which is most likely due to subthreshold activity [36]. The antagonism of neuronal activity in both optical and electrical recordings confirms neuronal signals in both these methods. These data also demonstrate the higher sensitivity of optical recording methods for subthreshold neuronal activity, where optical signals within the cell body are still prominent following TTX addition, whereas electrical recordings diminish significantly making identification of subthreshold events difficult.

**Fig 6:**
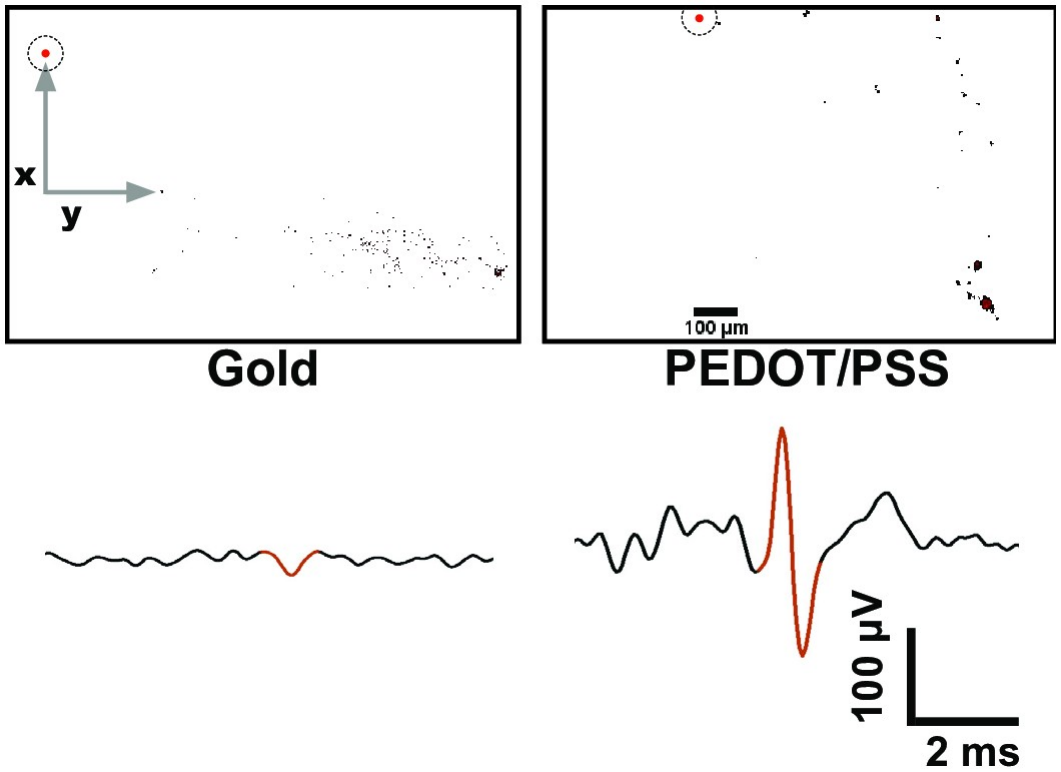
Optical image of gold (left) and PEDOT/PSS (right) microelectrode (displayed as red dot) with neuronal activity. The corresponding electrical recording for the optical image is shown below each image and represented as a black trace. The portion of the electrical recording which correlates with neuronal firing in the optical image is highlighted in red.

Following the successful validation of this combined optical/electrical system, it was used to characterise the recording performance of gold and PEDOT/PSS microelectrodes. More specifically, a traditional SNR metric was attributed to each electrode material, followed by its assessment through our own performance factor measurement which takes into account the distance of the signal source. Fig 6 shows two images with neuronal activity next to a gold or PEDOT/PSS microelectrode. The distance between neuron and electrode is clear and more robust calculations regarding electrode recording performance can be made through application of Eq 2 [37–39]. Eq 2 can be used to estimate the voltage at the microelectrode tip (*V*) at position *x, y, z* following an action potential, modeled as a transmembrane current source (*I*), at position *x’, y’, z’* (assuming an infinite volume conductor with homogenous extracellular electrical conductivity (*σ*)).

$$\begin{array}{c} V\left(x,y,z\right)=\frac{I}{4\pi \sigma \sqrt{{\left(x-x^{{}^{\prime }}\right)}^{2}+{\left(y-y^{{}^{\prime }}\right)}^{2}+{\left(z-z^{{}^{\prime }}\right)}^{2}}} \end{array}$$ (2)

The expected potential at the electrode site can then be divided by the actual potential recorded and a ‘performance factor’ (i.e. recorded potential/expected potential) can be associated with the microelectrode used. The performance factors of gold and PEDOT/PSS electrodes were calculated to be 0.02 and 0.06, respectively (Table 1). This calculation was performed using only the x and y plane, where *x,y* were microelectrode coordinates and *x’,y’* were coordinates of the neural process closest to the microelectrode. Solution conductivity was assumed to be 1.45 S m^−1^ [40], and a transmembrane current of 10 nA was used to model a neuronal action potential based on data from intracellular patch recordings—this value may differ and a more accurate calculation can be made by obtaining the real value for *I*.

**Table 1:** Calculation of microelectrode performance utilising the volume conduction theory to calculate an expected potential.

Electrode	Expected potential (*μ*V)	Recorded potential (*μ*V)	Performance factor
**Gold**	1189	20	0.02
**PEDOT/PSS**	3465	207	0.06

These results show that PEDOT/PSS microelectrodes are three-fold more effective in transducing biological currents, as predicted through impedance measurements. When comparing this method to SNR values as calculated through Eq 1 a large discrepancy between the results is noticed. SNR values were calculated to be 2.5 and 20 for gold and PEDOT/PSS, respectively. Traditionally, this would be reported as PEDOT/PSS having a 8-fold improvement over gold whereas in actual fact, the neuron was just further away.

In addition to electrode performance characterisation, the proposed simultaneous optical/electrical recording system has the potential to yield a wide range of data from neuronal populations such as extracellular spiking as well as VSD recorded neuronal parameters such as ion concentration membrane potential, subthreshold synaptic events and secondary messenger release [25]. The system could be further utilised to validate spike sorting algorithms through correlation of signal source and recorded microelectrode potential leading to the development of more robust detection methods.

## Conclusions

A system to record from neurons both electrically, through multielectrode arrays, and optically via VSDs was developed. This system consisted of (i) MEA slides which were modified with PEDOT/PSS to reduce impedance and improve transduction properties, (ii) a custom built amplfication system which was capable of amplifying neuronal potentials with low intrinsic noise and (iii) an optical recording system which successfully visualised neuronal membrane potential changes through the use of a high speed camera and VSDs. Limitations of the described system are present within the microelectrode array used, the specifications of the high speed camera and the use of an older generation of VSD, Di-4-ANEPPS. Utilisation of a MEA with microelectrodes separated by 50 *μ*m would allow for the visualisation of many electrodes within one frame, improving the statistical power of the analysis. Furthermore, high speed cameras with faster exposure times (<1 ms) and a new generation of VSD would both facilitate the temporal resolution and image quality of the method. Electrically recorded action potentials were correlated with optical images and neuronal origin was confirmed via TTX. TTX addition also demonstrated the ability of optical imaging techniques to visualise subthreshold neuronal activity. The application of this system to microelectrode characterisation has highlighted discrepancies in recording performance when compared to traditional SNR calculation methods. The presented method allows for an incorporation of signal distance from the microelectrode tips, making quantification of recording performance more reliable. This system will allow for more in-depth studies on *in-vitro* neuronal populations through MEAs and will pave the path for validation of electrode performance and spike sorting techniques.

## Supporting information

S1 File PCB schematic for amplifier. (ZIP)

S2 File LabVIEW code. (ZIP)

S3 File Manuscript Data. (ZIP)
