# Supplementary material for: A simultaneous optical and electrical in-vitro neuronal recording system to evaluate microelectrode performance
Source: PLoS One. 2020 Aug 20;15(8):e0237709. doi: 10.1371/journal.pone.0237709 (PMC7440637; doi:10.1371/journal.pone.0237709)
Supplement: S2 File — (ZIP) [file pone.0237709.s002.zip › MEA DAQ/MEA DAQ/documentation/Continuous Measurement and Logging (NI-DAQmx) Documentation.html]

Continuous Measurement and Logging (NI-DAQmx).html


# Continuous Measurement and Logging (NI-DAQmx)

The Continuous Measurement and Logging (NI-DAQmx) sample project acquires measurements continuously and logs them to disk. It executes five loops in parallel:

- Event handling (Main.vi)—The Event Handling Loop that produces messages based on front panel events, such as the user clicking **Start** or **Settings**.
- User interface messaging (Main.vi)—A Message Handling Loop that receives messages from the Event Handling Loop and responds by sending messages to the other Message Handling Loops.
- Acquiring data (Acquisition.lvlib:Acquisition Message Loop.vi)—A Message Handling Loop that continuously acquires data.
- Logging data (Logging.lvlib:Logging Message Loop.vi)—A Message Handling Loop that continuously logs acquired data.
- Displaying data (Main.vi)—A While Loop that updates the waveform chart with acquired data.

This sample project also features a **Settings** dialog box (Settings.lvlib) you can use to configure the application.

This sample project is based on the Queued Message Handler template. Refer to the Queued Message Handler template and its documentation, available from the Create Project dialog box, for information about how this template works.

## Developer Walkthrough

Refer to ni.com for a developer walkthrough of the sample projects.

## System Requirements

## Requirements

LabVIEW Base Package and NI-DAQmx driver software.

## Project Diagram

This sample project uses the NI-DAQmx API to configure and acquire a measurement with hardware I/O.

## Use Cases

The Continuous Measurement and Logging (NI-DAQmx) sample project is designed for a continuous measurement application that requires a responsive user interface; that is, users should be able to click buttons even while the application is executing another command.

## Running this Sample Project

1. In the **Project Explorer** window, open and run Main.vi.
2. Click **Start**. The program begins acquiring waveform data.
3. Click the other front panel buttons to explore the sample project.

## Customizing this Sample Project

If the default logging behavior does not meet the needs of your application, you can modify this sample project in the following ways:

- To specify where data is logged, run Main.vi, click **Settings**, and use the **Log File Path** control. By default, this template logs data to LabVIEW Data\Logged Data.tdms, where LabVIEW Data is the LabVIEW Data folder.
- To specify a trigger for logging data, run Main.vi, click **Settings**, and use the **Log When Triggered** control. You can specify a time or threshold value to trigger logging. You can also manually trigger logging by using the **Force Trigger** control on Main.vi.
- To change the data logging mechanism, modify Logging.lvlib:Logging Message Loop.vi. For example, you could modify this VI to stream acquired data across a network or to disk. By default, this template uses notifiers to control when data is logged.
- To change the code that writes data to disk, modify Logging.lvlib:Log Data.vi. For example, you could use the Export Waveforms to Spreadsheet File or Write to Spreadsheet File VIs.
- To change or add path and file refnums that are needed for data logging, modify Logging.lvlib:Logging Configuration.ctl.
- To change the type of data this acquisition acquires, open Configure Hardware.vi, and select a different instance of the DAQmx Create Virtual Channel VI.

If the default logging behavior is too slow for your application, you can use the DAQmx Start New File and DAQmx Configure Logging VIs. Refer to the VI documentation for more information.

## LabVIEW Features and Concepts Used

- Case structures
- Clusters
- Control refnums
- NI-DAQmx VIs
- Enums
- Error clusters
- Event structures
- LabVIEW Schema VIs and Functions
- Notifiers
- Parallelism
- Producer/Consumer design pattern
- Project libraries
- Queues
- Shift registers
- State machines
- Typedefs
- Value change events
- User events
- While Loops

---

## Important Information

**Copyright**

© 2013 National Instruments. All rights reserved.

Under the copyright laws, this publication may not be reproduced or transmitted in any form, electronic or mechanical, including photocopying, recording, storing in an information retrieval system, or translating, in whole or in part, without the prior written consent of National Instruments Corporation.

National Instruments respects the intellectual property of others, and we ask our users to do the same. NI software is protected by copyright and other intellectual property laws. Where NI software may be used to reproduce software or other materials belonging to others, you may use NI software only to reproduce materials that you may reproduce in accordance with the terms of any applicable license or other legal restriction.

**End-User License Agreements and Third-Party Legal Notices**

You can find end-user license agreements (EULAs) and third-party legal notices in the following locations:

- Notices are located in the <National Instruments>\\_Legal Information and <National Instruments> directories.
- EULAs are located in the <National Instruments>\Shared\MDF\Legal\license directory.
- Review <National Instruments>\\_Legal Information.txt for information on including legal information in installers built with NI products.

**Trademarks**

LabVIEW, National Instruments, NI, ni.com, the National Instruments corporate logo, and the Eagle logo are trademarks of National Instruments Corporation. Refer to the *Trademark Information* at ni.com/trademarks for other National Instruments trademarks.

Other product and company names mentioned herein are trademarks or trade names of their respective companies.

**Patents**

For patents covering the National Instruments products/technology, refer to the appropriate location: **Help»Patents** in your software, the patents.txt file on your media, or the National Instruments Patent Notice at ni.com/patents.
