# Supplementary figures and images for: A simultaneous optical and electrical in-vitro neuronal recording system to evaluate microelectrode performance (part 1 of 2)
Source: PLoS One. 2020 Aug 20;15(8):e0237709. doi: 10.1371/journal.pone.0237709 (PMC7440637; doi:10.1371/journal.pone.0237709)

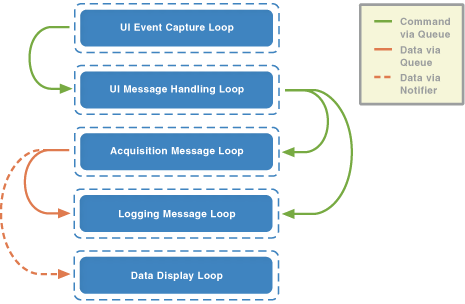

Supplement: S2 File — (ZIP) [file pone.0237709.s002.zip › MEA DAQ/MEA DAQ/documentation/loc_cont_meas_daqmx.gif]

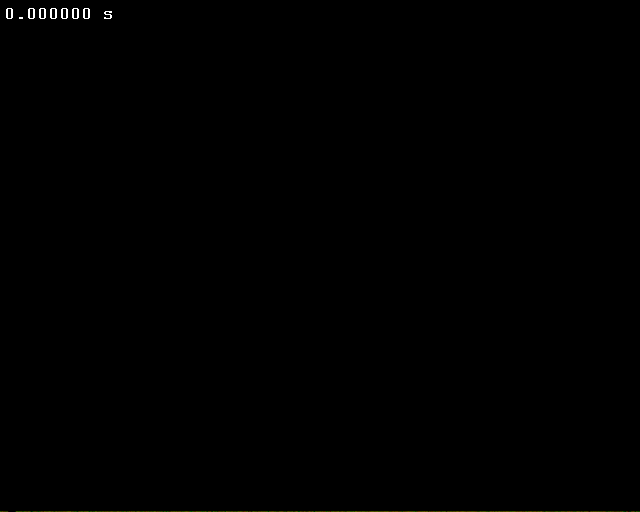

Supplement: S3 File — (ZIP) [file pone.0237709.s003.zip › PEDOT Electrode Recording/Position000000.tif]

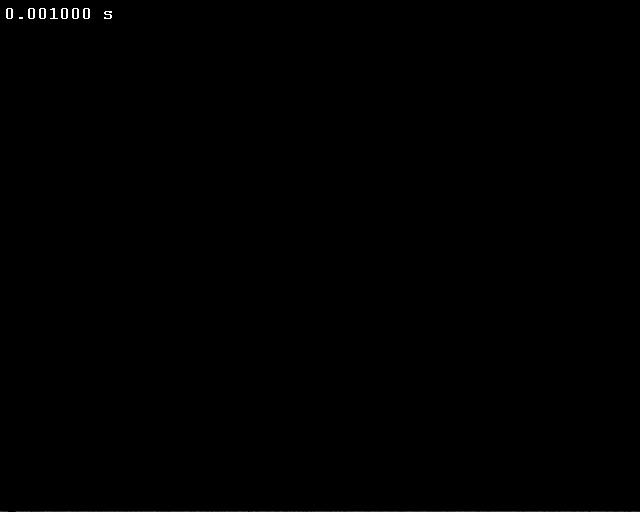

Supplement: S3 File — (ZIP) [file pone.0237709.s003.zip › PEDOT Electrode Recording/Position000001.tif]

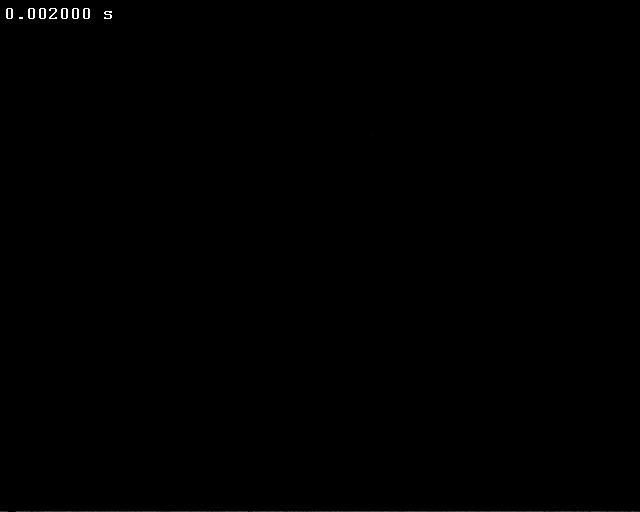

Supplement: S3 File — (ZIP) [file pone.0237709.s003.zip › PEDOT Electrode Recording/Position000002.tif]

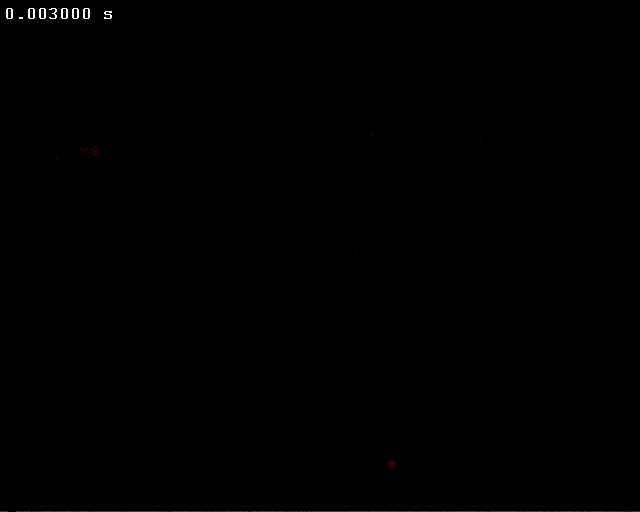

Supplement: S3 File — (ZIP) [file pone.0237709.s003.zip › PEDOT Electrode Recording/Position000003.tif]

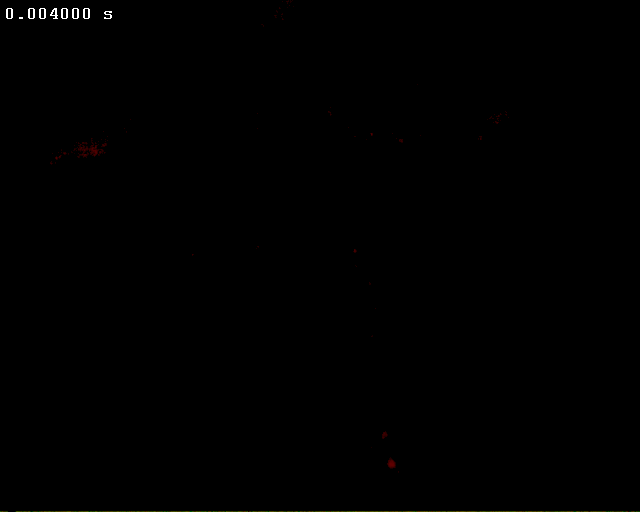

Supplement: S3 File — (ZIP) [file pone.0237709.s003.zip › PEDOT Electrode Recording/Position000004.tif]

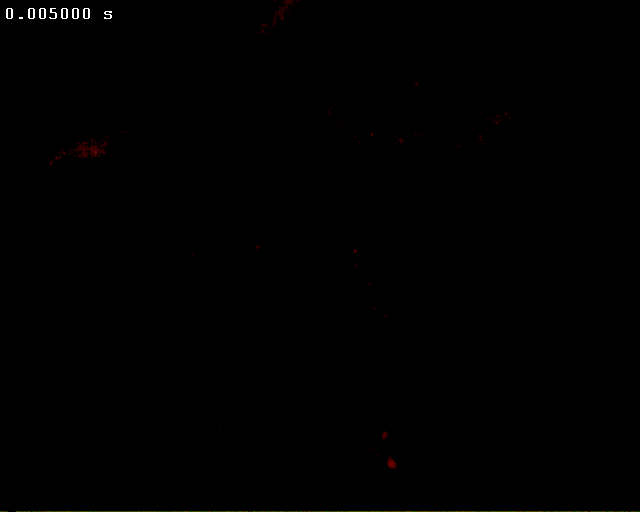

Supplement: S3 File — (ZIP) [file pone.0237709.s003.zip › PEDOT Electrode Recording/Position000005.tif]

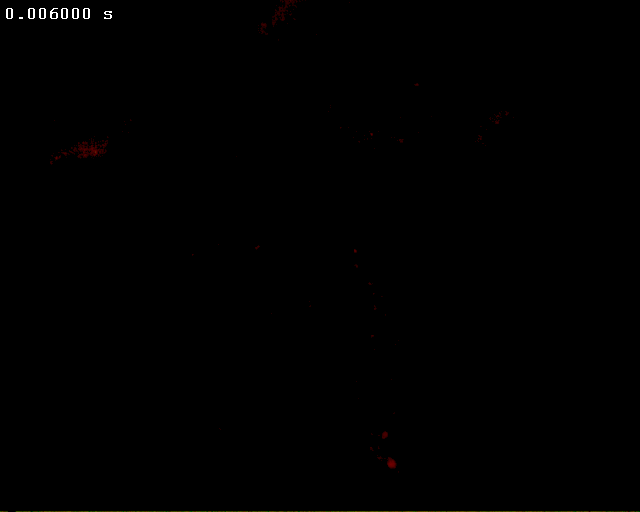

Supplement: S3 File — (ZIP) [file pone.0237709.s003.zip › PEDOT Electrode Recording/Position000006.tif]

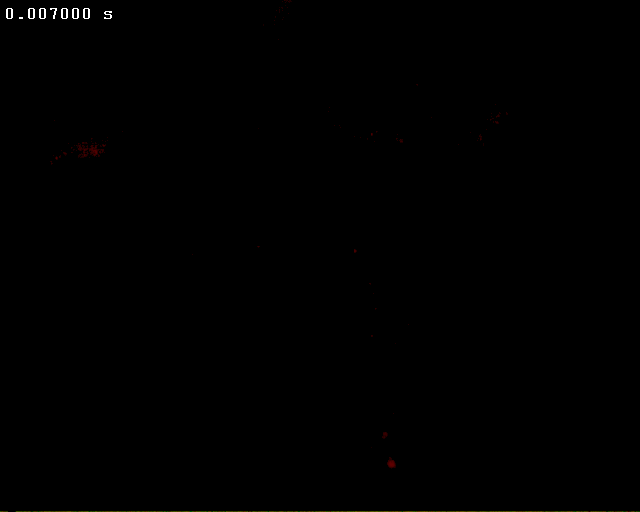

Supplement: S3 File — (ZIP) [file pone.0237709.s003.zip › PEDOT Electrode Recording/Position000007.tif]

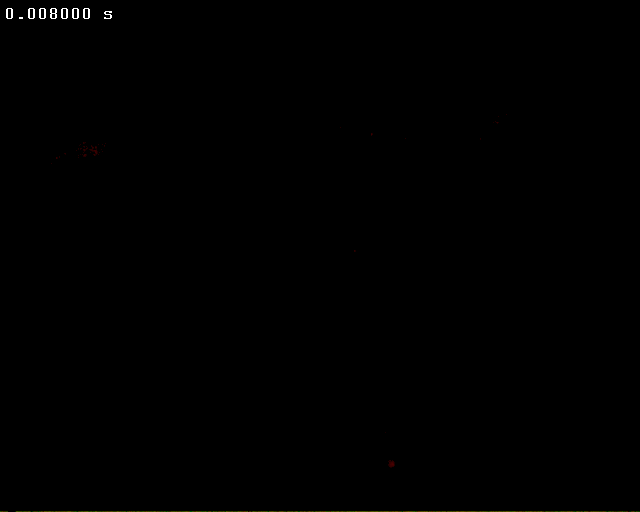

Supplement: S3 File — (ZIP) [file pone.0237709.s003.zip › PEDOT Electrode Recording/Position000008.tif]

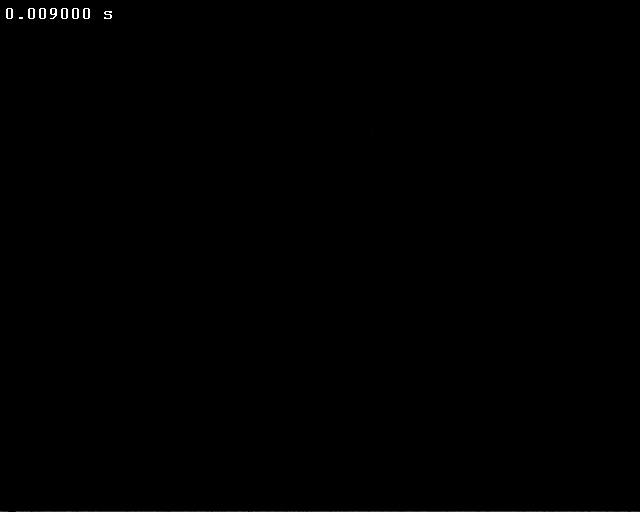

Supplement: S3 File — (ZIP) [file pone.0237709.s003.zip › PEDOT Electrode Recording/Position000009.tif]

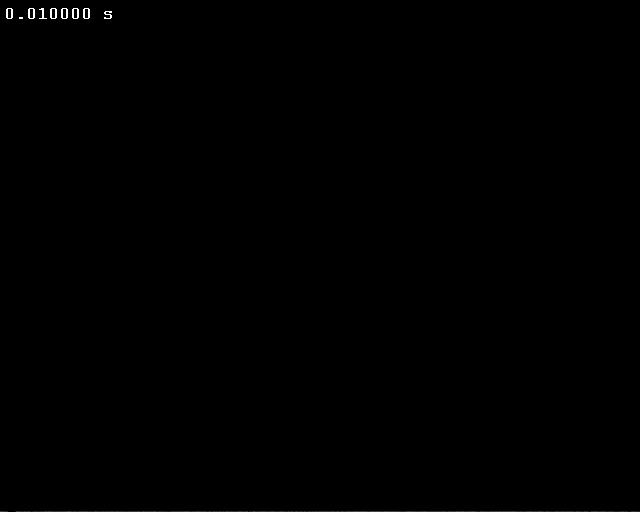

Supplement: S3 File — (ZIP) [file pone.0237709.s003.zip › PEDOT Electrode Recording/Position000010.tif]

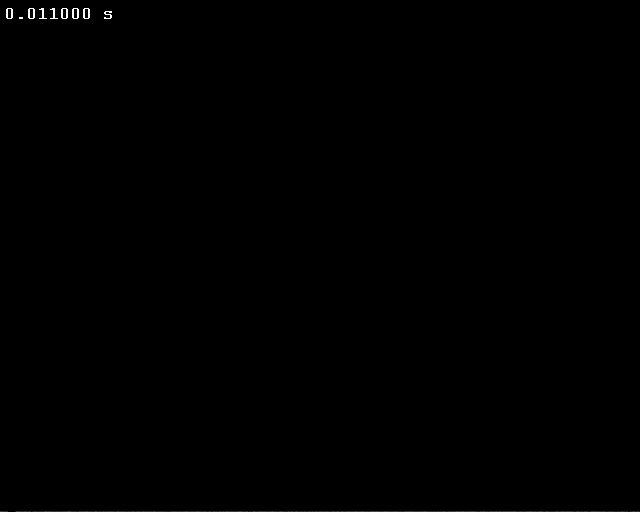

Supplement: S3 File — (ZIP) [file pone.0237709.s003.zip › PEDOT Electrode Recording/Position000011.tif]

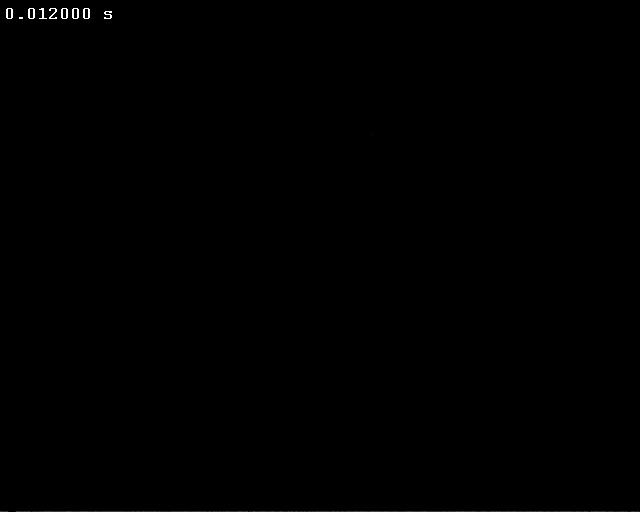

Supplement: S3 File — (ZIP) [file pone.0237709.s003.zip › PEDOT Electrode Recording/Position000012.tif]

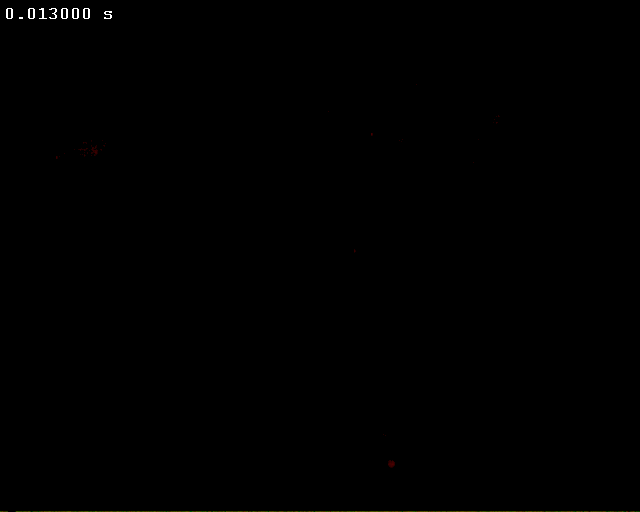

Supplement: S3 File — (ZIP) [file pone.0237709.s003.zip › PEDOT Electrode Recording/Position000013.tif]

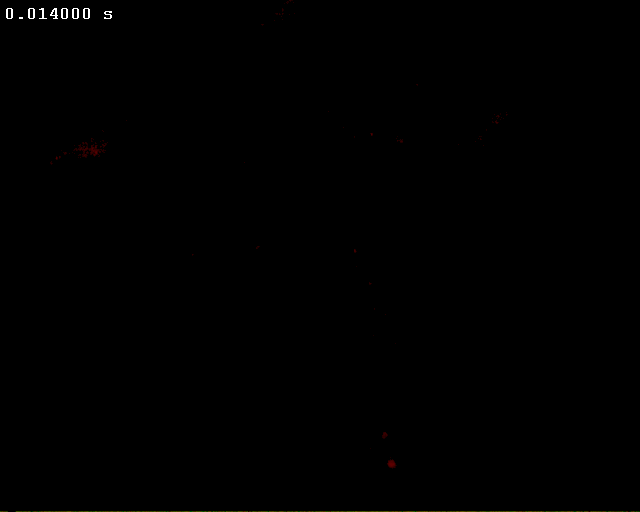

Supplement: S3 File — (ZIP) [file pone.0237709.s003.zip › PEDOT Electrode Recording/Position000014.tif]

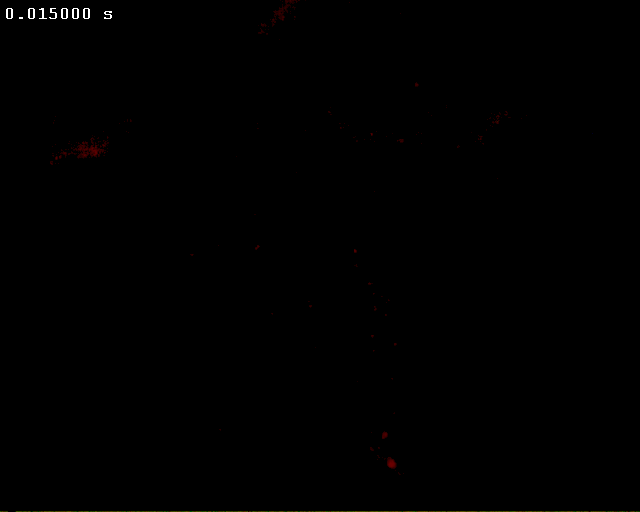

Supplement: S3 File — (ZIP) [file pone.0237709.s003.zip › PEDOT Electrode Recording/Position000015.tif]

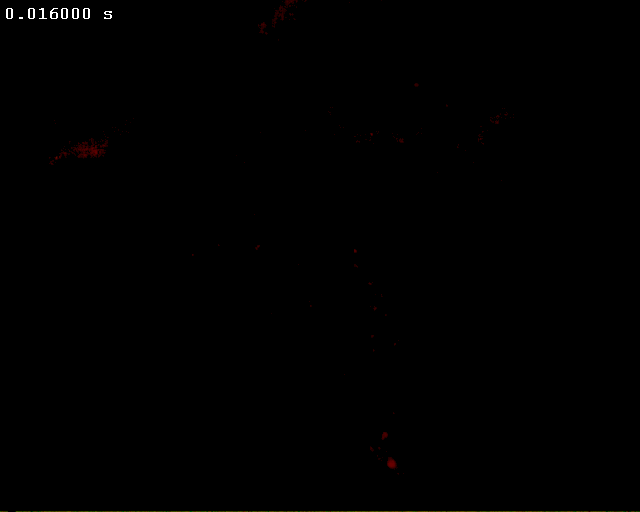

Supplement: S3 File — (ZIP) [file pone.0237709.s003.zip › PEDOT Electrode Recording/Position000016.tif]

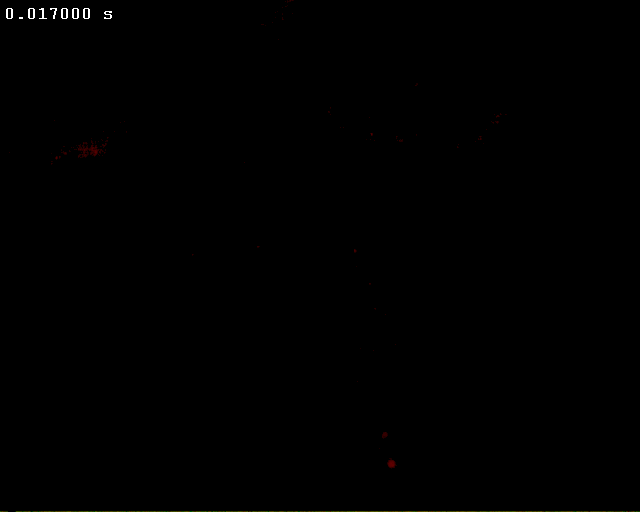

Supplement: S3 File — (ZIP) [file pone.0237709.s003.zip › PEDOT Electrode Recording/Position000017.tif]

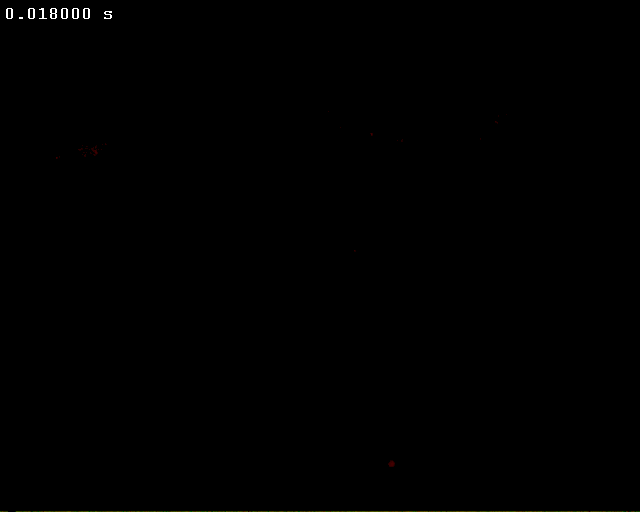

Supplement: S3 File — (ZIP) [file pone.0237709.s003.zip › PEDOT Electrode Recording/Position000018.tif]

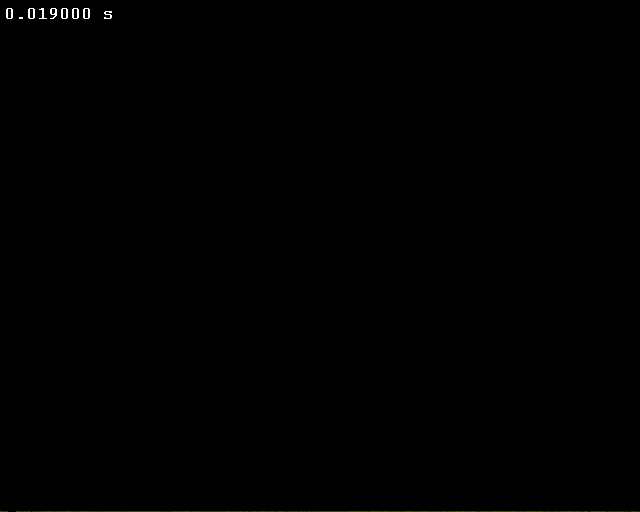

Supplement: S3 File — (ZIP) [file pone.0237709.s003.zip › PEDOT Electrode Recording/Position000019.tif]

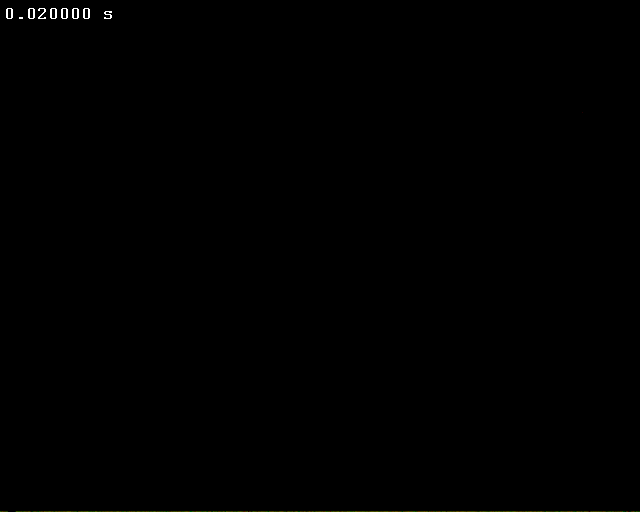

Supplement: S3 File — (ZIP) [file pone.0237709.s003.zip › PEDOT Electrode Recording/Position000020.tif]

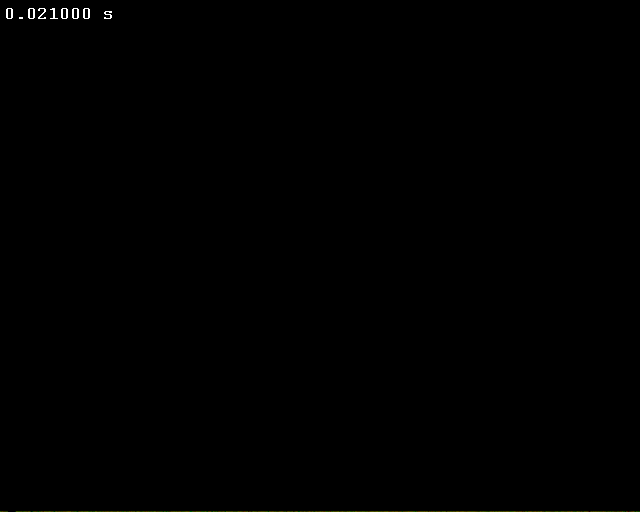

Supplement: S3 File — (ZIP) [file pone.0237709.s003.zip › PEDOT Electrode Recording/Position000021.tif]

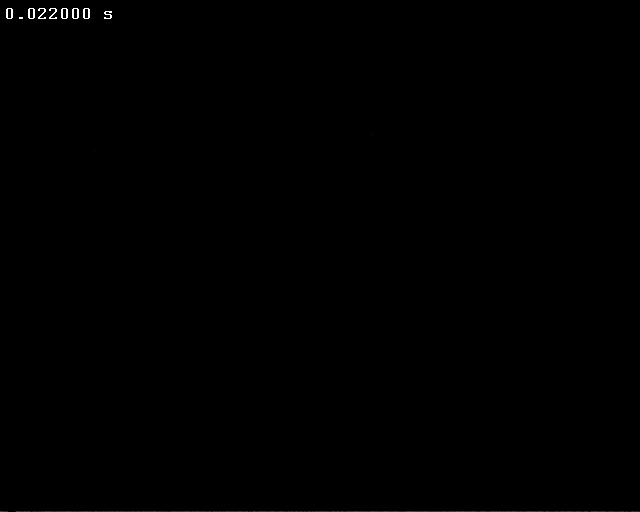

Supplement: S3 File — (ZIP) [file pone.0237709.s003.zip › PEDOT Electrode Recording/Position000022.tif]

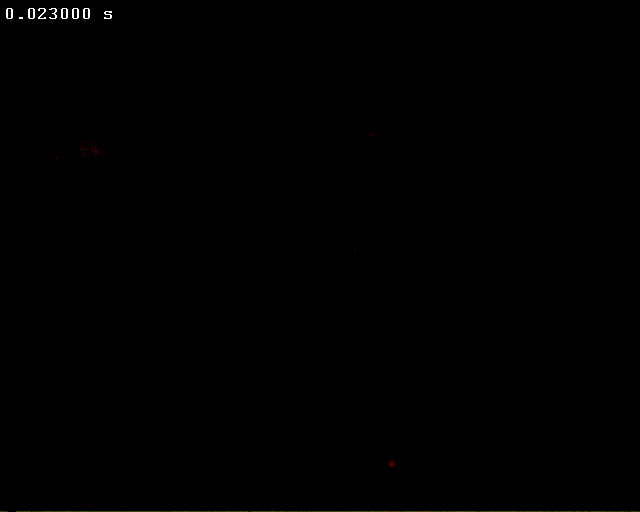

Supplement: S3 File — (ZIP) [file pone.0237709.s003.zip › PEDOT Electrode Recording/Position000023.tif]

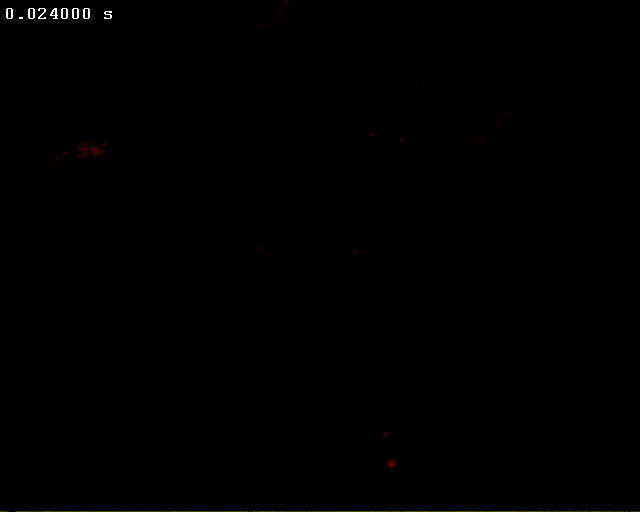

Supplement: S3 File — (ZIP) [file pone.0237709.s003.zip › PEDOT Electrode Recording/Position000024.tif]

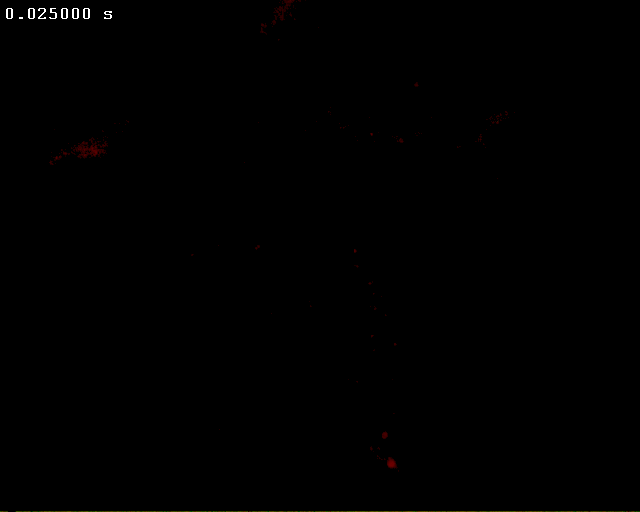

Supplement: S3 File — (ZIP) [file pone.0237709.s003.zip › PEDOT Electrode Recording/Position000025.tif]

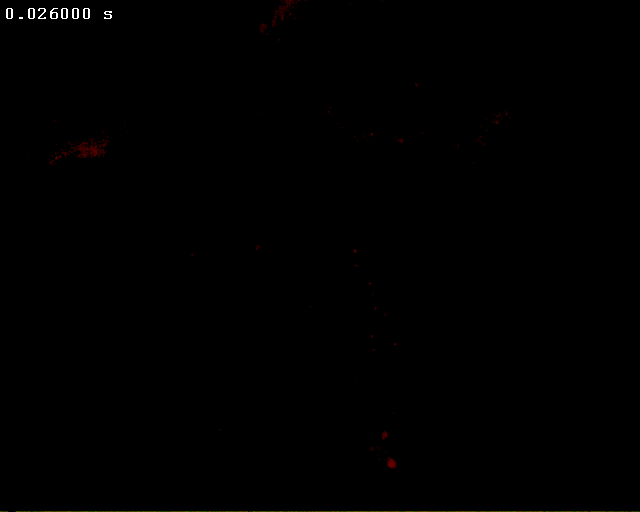

Supplement: S3 File — (ZIP) [file pone.0237709.s003.zip › PEDOT Electrode Recording/Position000026.tif]

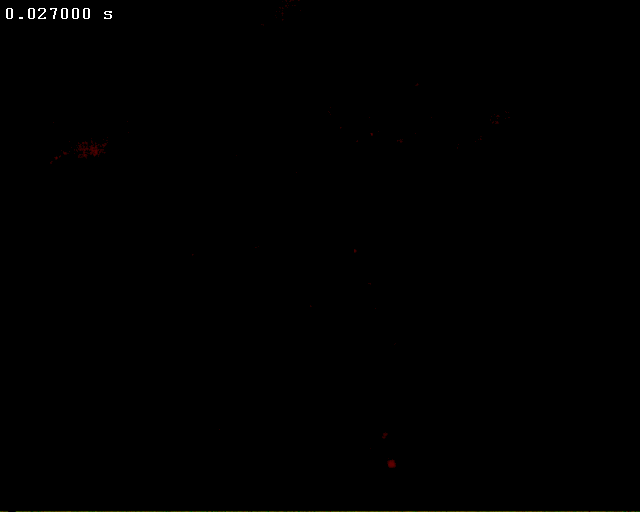

Supplement: S3 File — (ZIP) [file pone.0237709.s003.zip › PEDOT Electrode Recording/Position000027.tif]

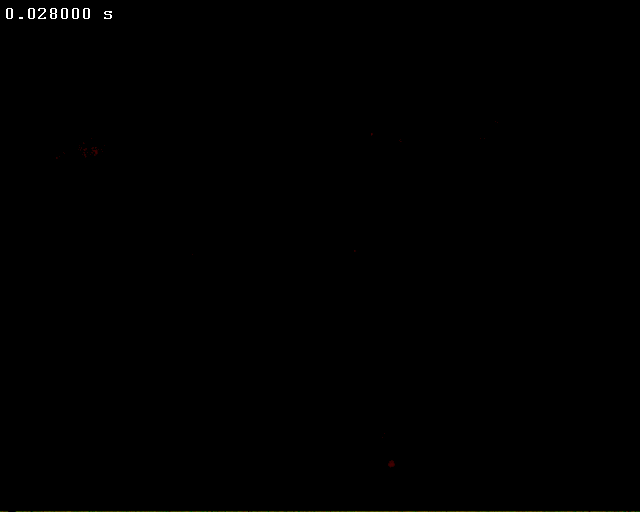

Supplement: S3 File — (ZIP) [file pone.0237709.s003.zip › PEDOT Electrode Recording/Position000028.tif]

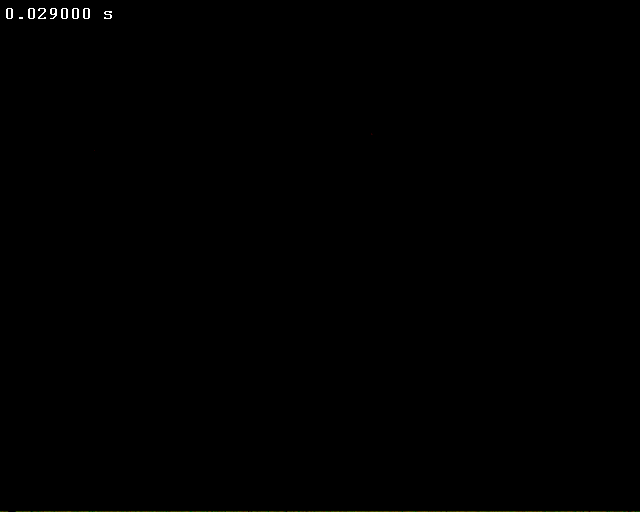

Supplement: S3 File — (ZIP) [file pone.0237709.s003.zip › PEDOT Electrode Recording/Position000029.tif]

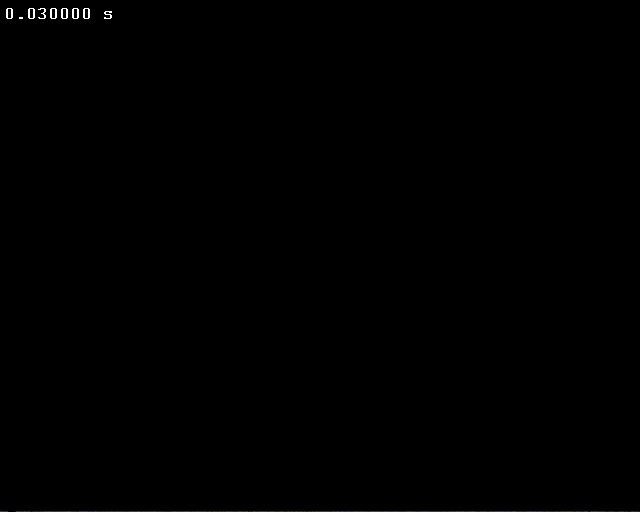

Supplement: S3 File — (ZIP) [file pone.0237709.s003.zip › PEDOT Electrode Recording/Position000030.tif]

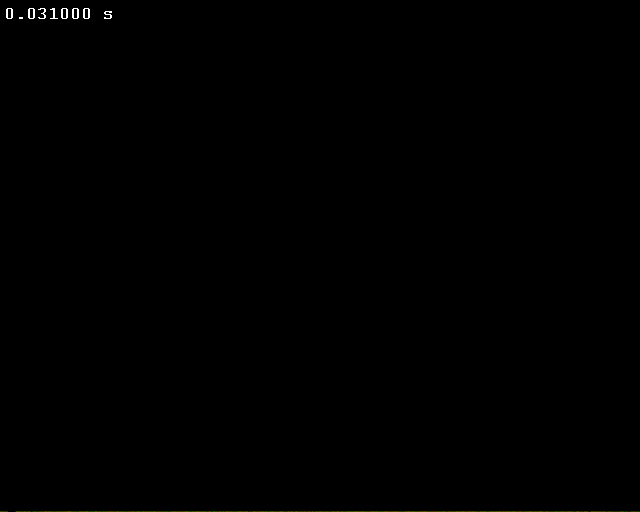

Supplement: S3 File — (ZIP) [file pone.0237709.s003.zip › PEDOT Electrode Recording/Position000031.tif]

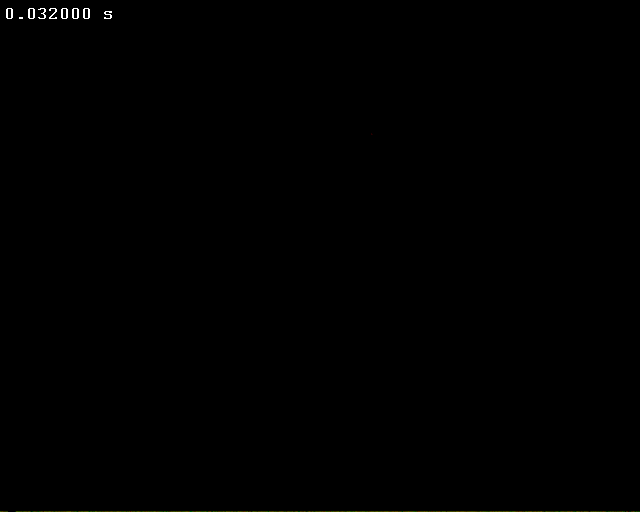

Supplement: S3 File — (ZIP) [file pone.0237709.s003.zip › PEDOT Electrode Recording/Position000032.tif]

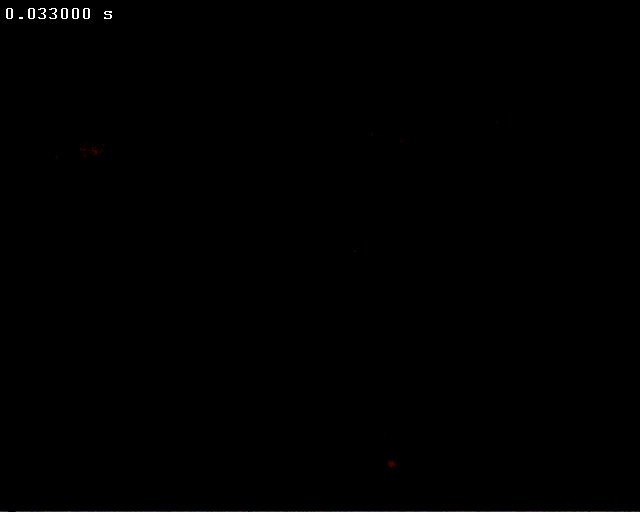

Supplement: S3 File — (ZIP) [file pone.0237709.s003.zip › PEDOT Electrode Recording/Position000033.tif]

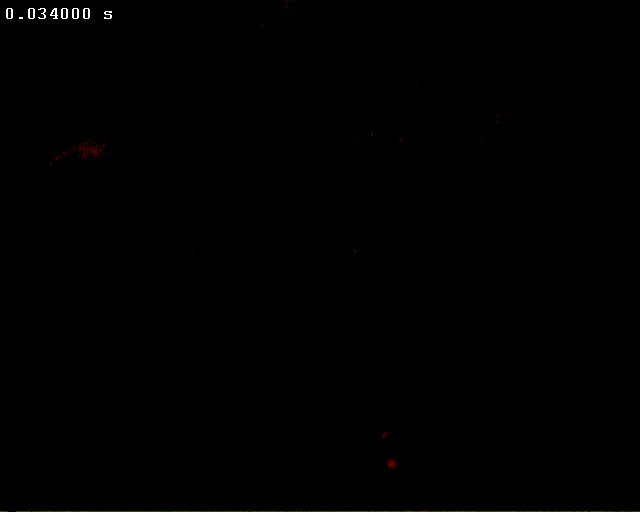

Supplement: S3 File — (ZIP) [file pone.0237709.s003.zip › PEDOT Electrode Recording/Position000034.tif]

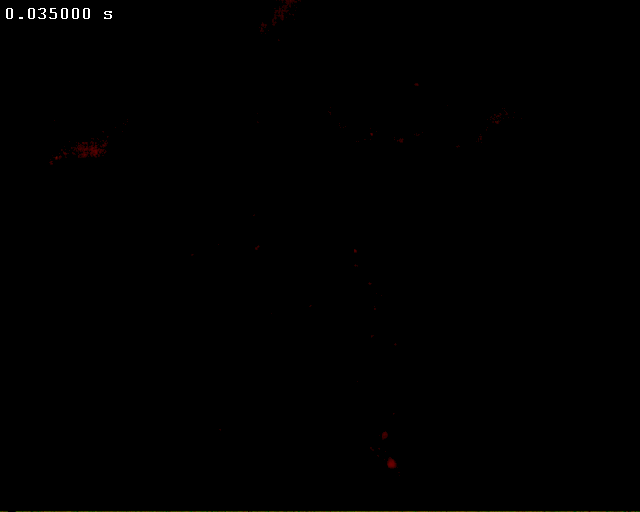

Supplement: S3 File — (ZIP) [file pone.0237709.s003.zip › PEDOT Electrode Recording/Position000035.tif]

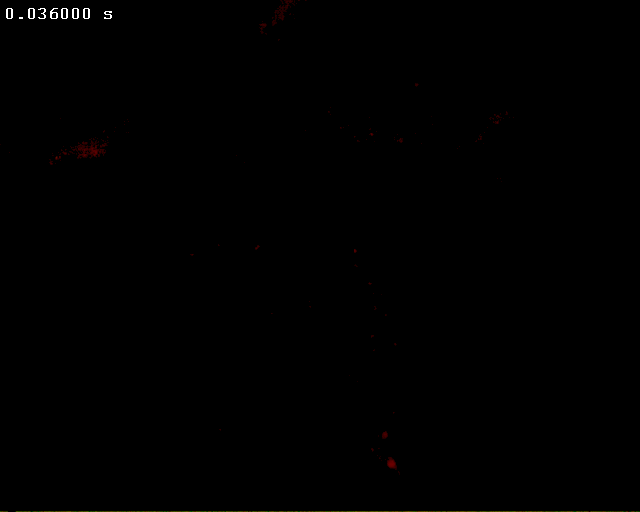

Supplement: S3 File — (ZIP) [file pone.0237709.s003.zip › PEDOT Electrode Recording/Position000036.tif]

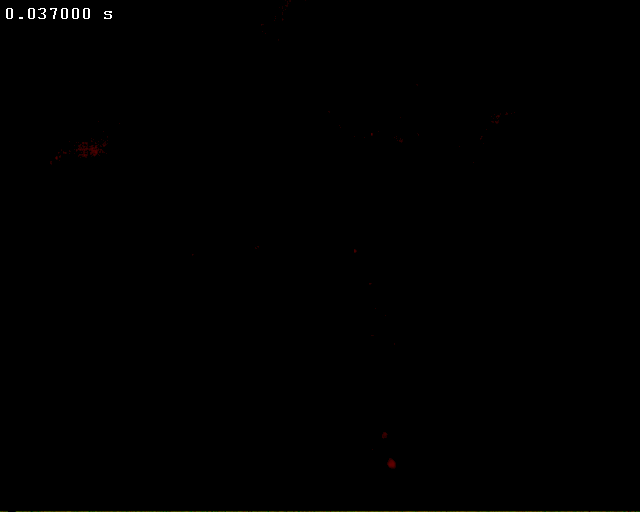

Supplement: S3 File — (ZIP) [file pone.0237709.s003.zip › PEDOT Electrode Recording/Position000037.tif]

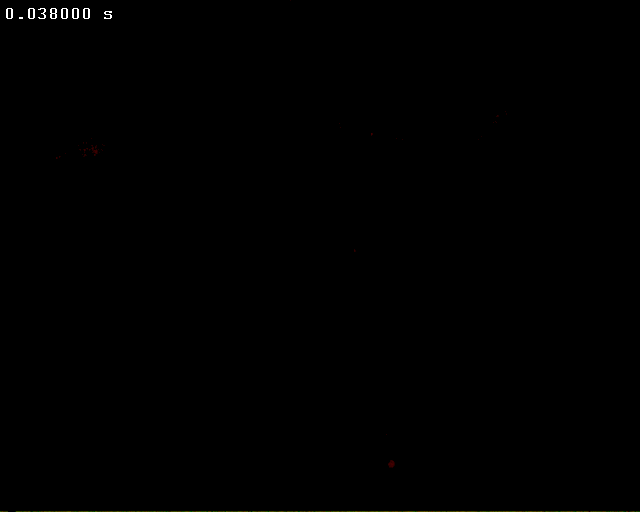

Supplement: S3 File — (ZIP) [file pone.0237709.s003.zip › PEDOT Electrode Recording/Position000038.tif]

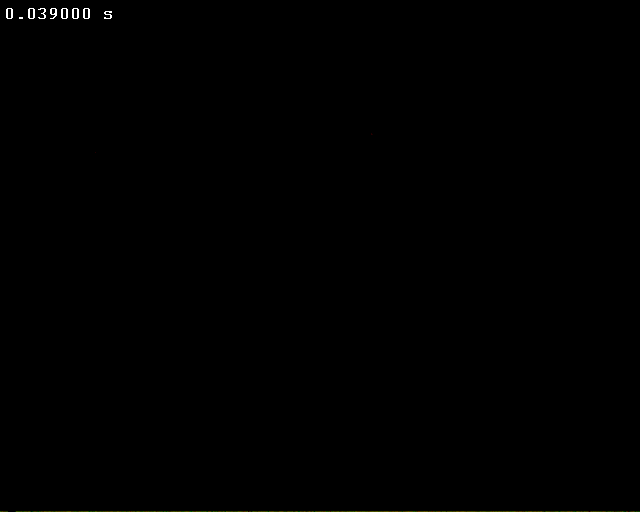

Supplement: S3 File — (ZIP) [file pone.0237709.s003.zip › PEDOT Electrode Recording/Position000039.tif]

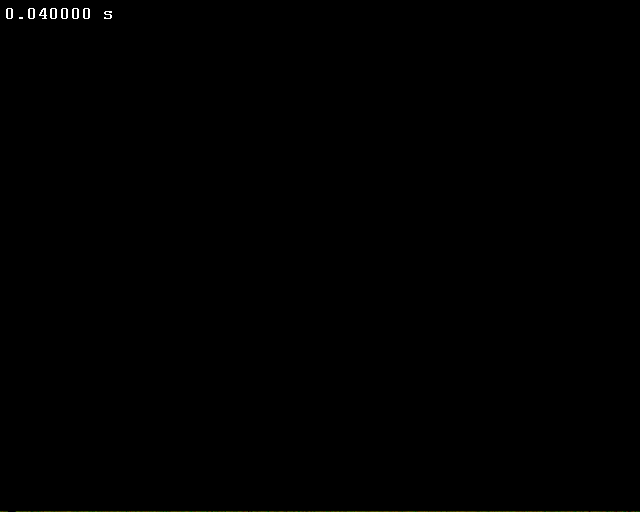

Supplement: S3 File — (ZIP) [file pone.0237709.s003.zip › PEDOT Electrode Recording/Position000040.tif]

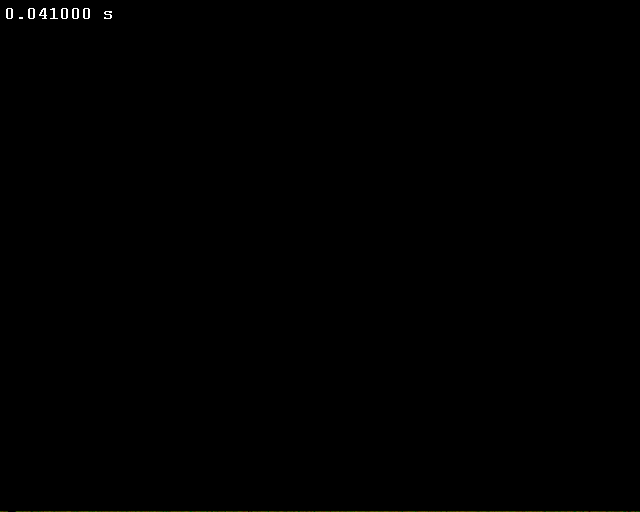

Supplement: S3 File — (ZIP) [file pone.0237709.s003.zip › PEDOT Electrode Recording/Position000041.tif]

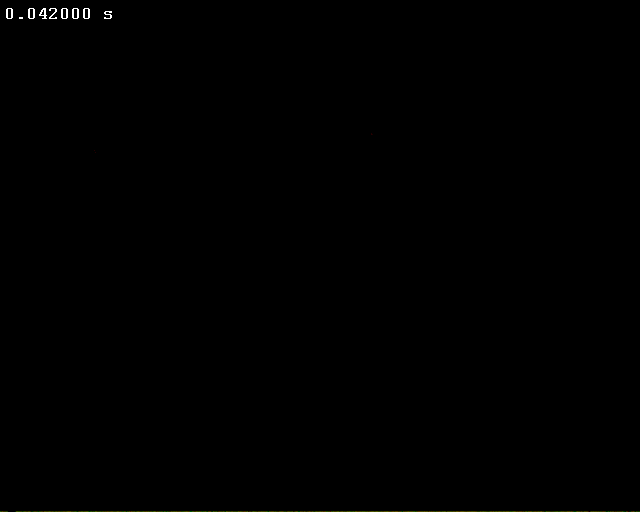

Supplement: S3 File — (ZIP) [file pone.0237709.s003.zip › PEDOT Electrode Recording/Position000042.tif]

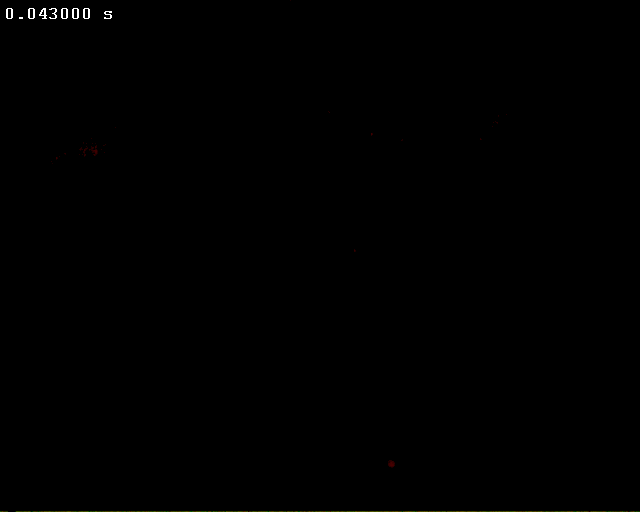

Supplement: S3 File — (ZIP) [file pone.0237709.s003.zip › PEDOT Electrode Recording/Position000043.tif]

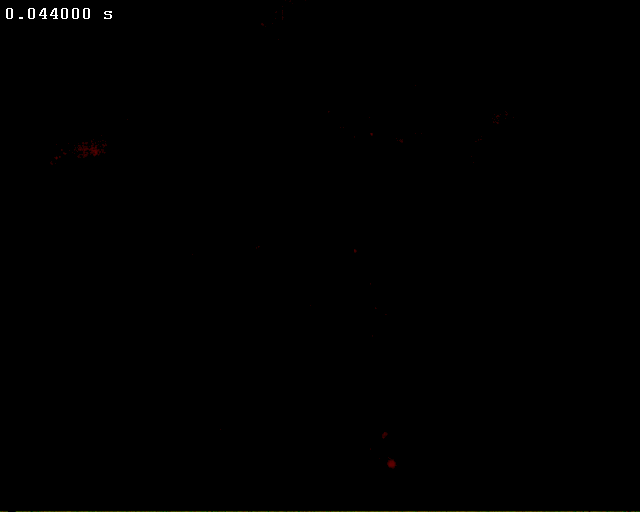

Supplement: S3 File — (ZIP) [file pone.0237709.s003.zip › PEDOT Electrode Recording/Position000044.tif]

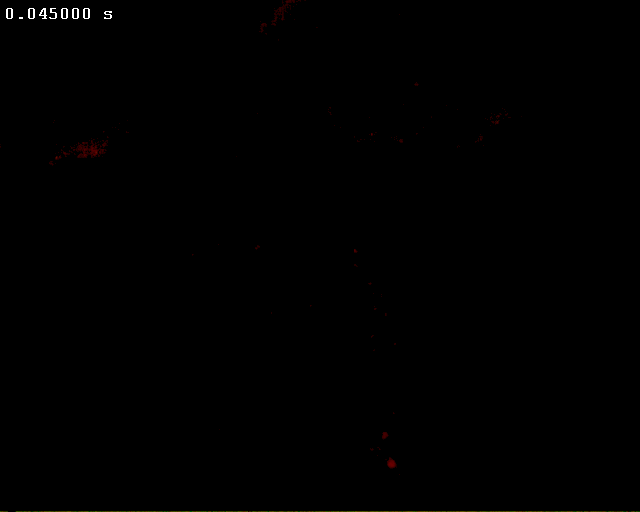

Supplement: S3 File — (ZIP) [file pone.0237709.s003.zip › PEDOT Electrode Recording/Position000045.tif]

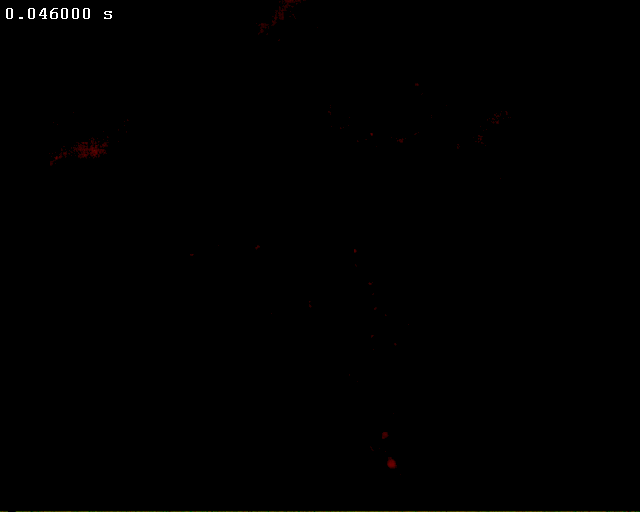

Supplement: S3 File — (ZIP) [file pone.0237709.s003.zip › PEDOT Electrode Recording/Position000046.tif]

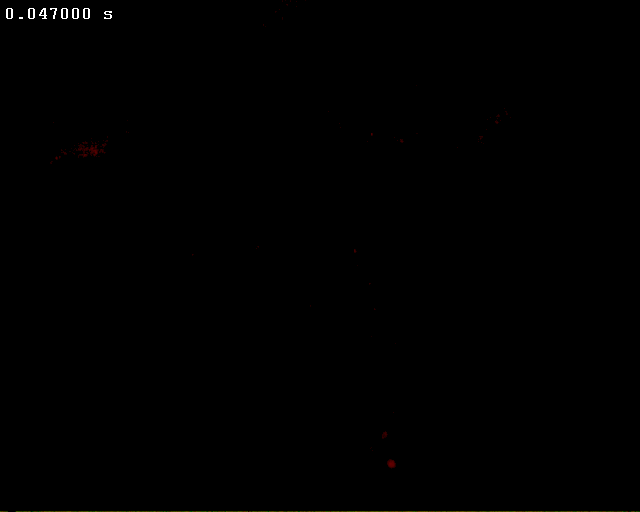

Supplement: S3 File — (ZIP) [file pone.0237709.s003.zip › PEDOT Electrode Recording/Position000047.tif]

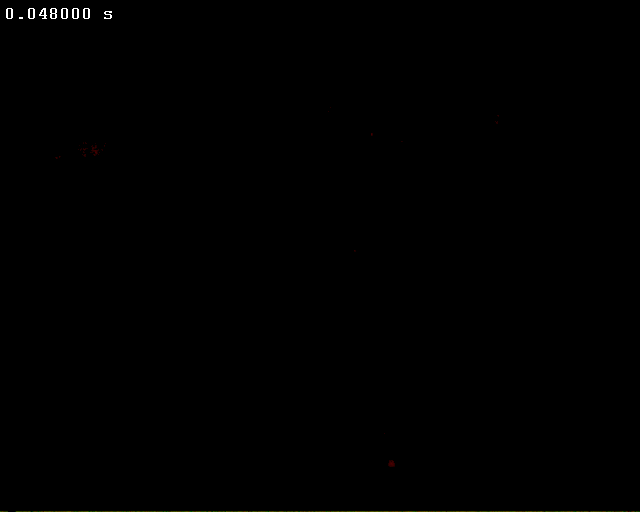

Supplement: S3 File — (ZIP) [file pone.0237709.s003.zip › PEDOT Electrode Recording/Position000048.tif]

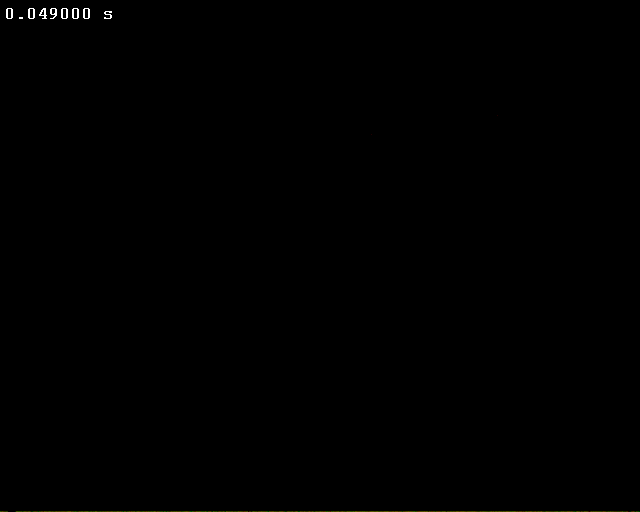

Supplement: S3 File — (ZIP) [file pone.0237709.s003.zip › PEDOT Electrode Recording/Position000049.tif]

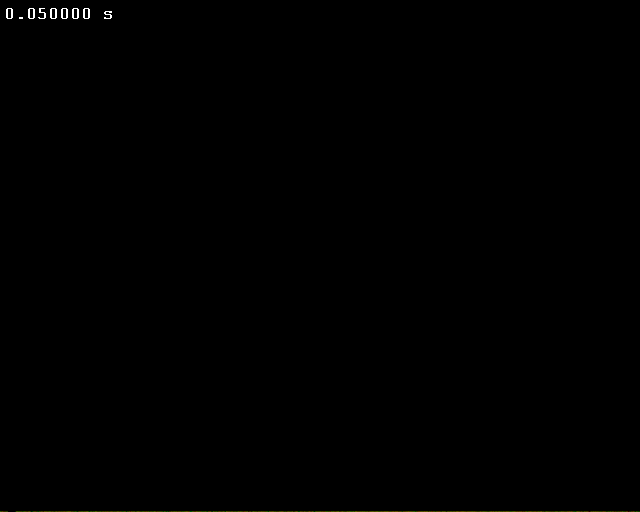

Supplement: S3 File — (ZIP) [file pone.0237709.s003.zip › PEDOT Electrode Recording/Position000050.tif]

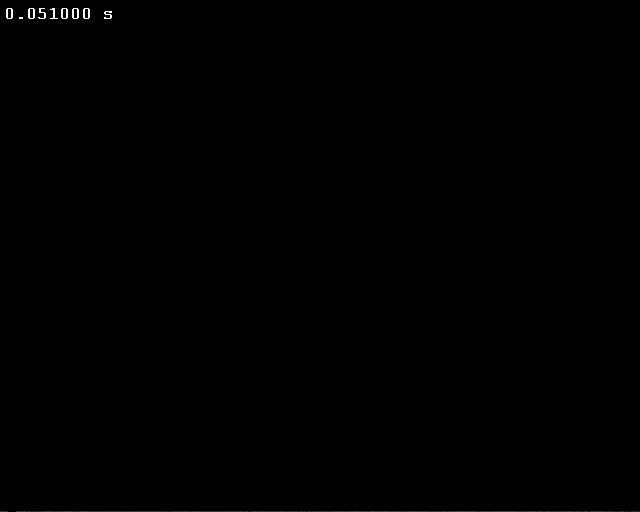

Supplement: S3 File — (ZIP) [file pone.0237709.s003.zip › PEDOT Electrode Recording/Position000051.tif]

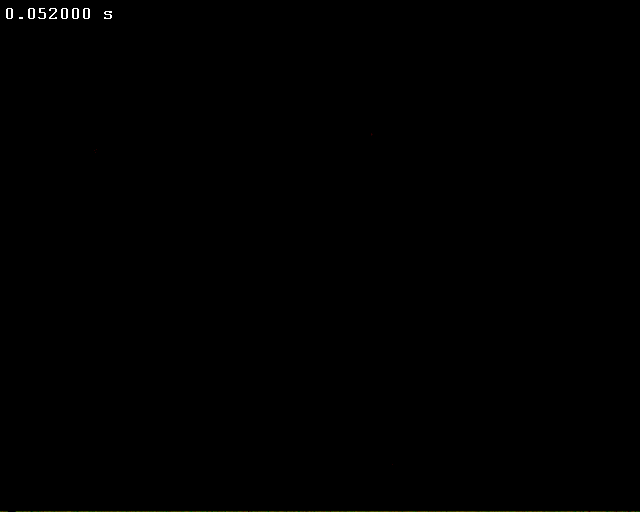

Supplement: S3 File — (ZIP) [file pone.0237709.s003.zip › PEDOT Electrode Recording/Position000052.tif]

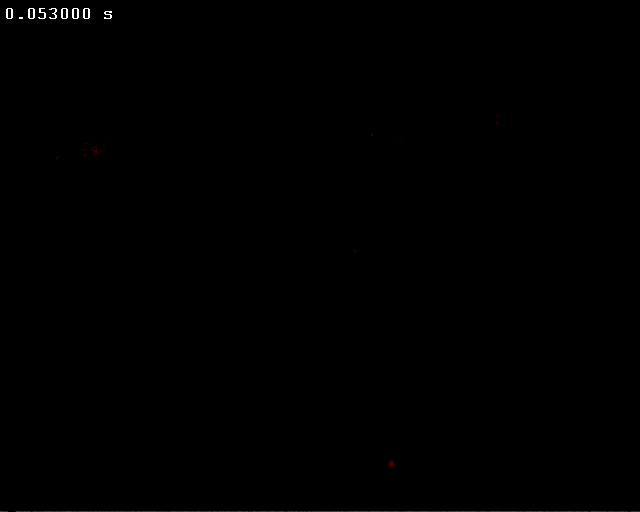

Supplement: S3 File — (ZIP) [file pone.0237709.s003.zip › PEDOT Electrode Recording/Position000053.tif]

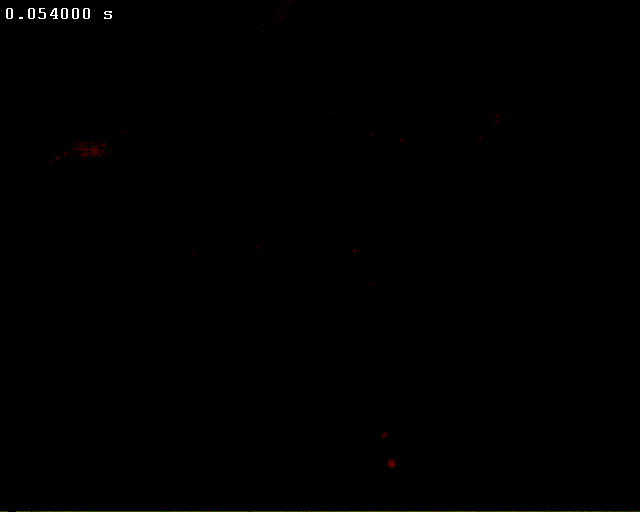

Supplement: S3 File — (ZIP) [file pone.0237709.s003.zip › PEDOT Electrode Recording/Position000054.tif]

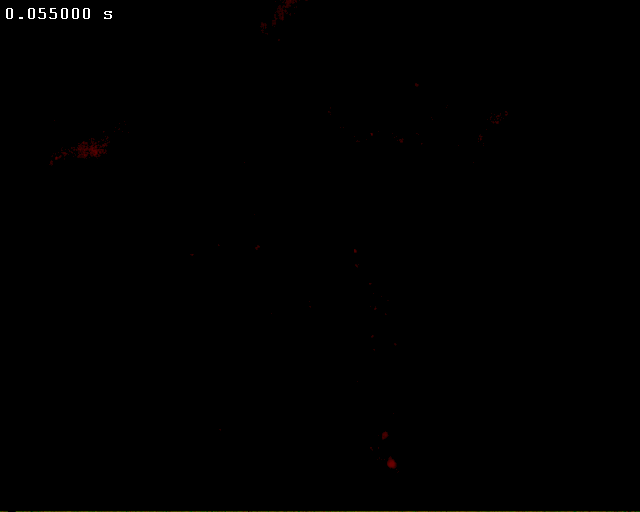

Supplement: S3 File — (ZIP) [file pone.0237709.s003.zip › PEDOT Electrode Recording/Position000055.tif]

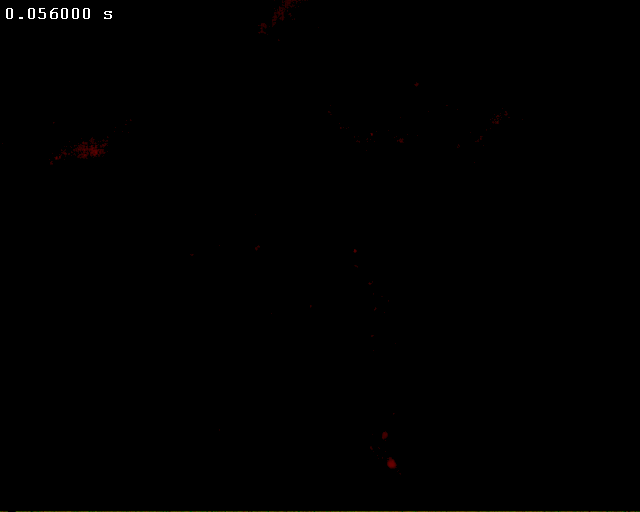

Supplement: S3 File — (ZIP) [file pone.0237709.s003.zip › PEDOT Electrode Recording/Position000056.tif]

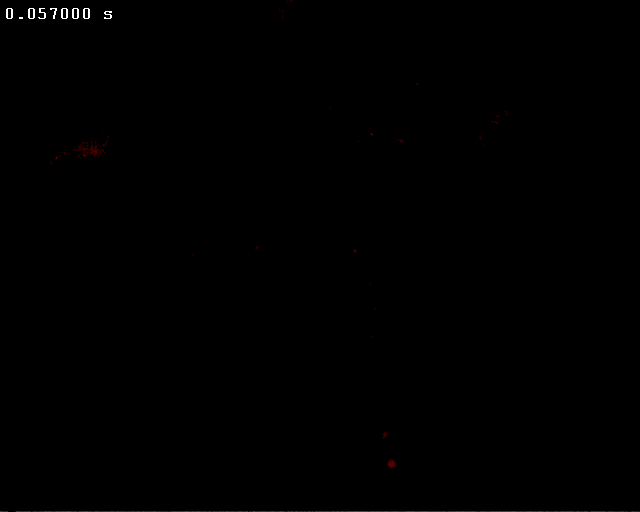

Supplement: S3 File — (ZIP) [file pone.0237709.s003.zip › PEDOT Electrode Recording/Position000057.tif]

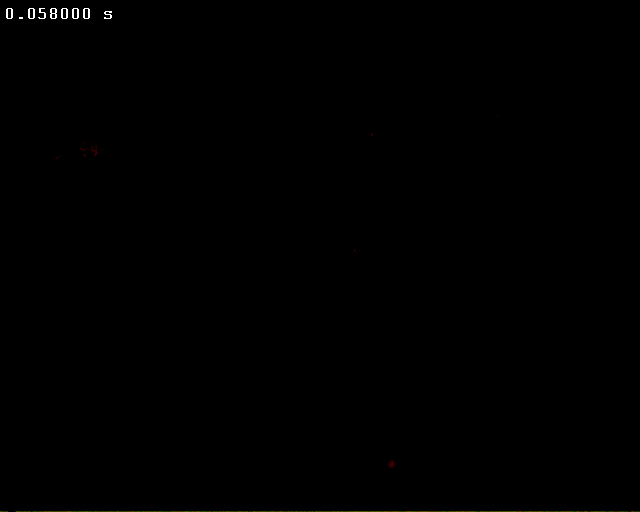

Supplement: S3 File — (ZIP) [file pone.0237709.s003.zip › PEDOT Electrode Recording/Position000058.tif]

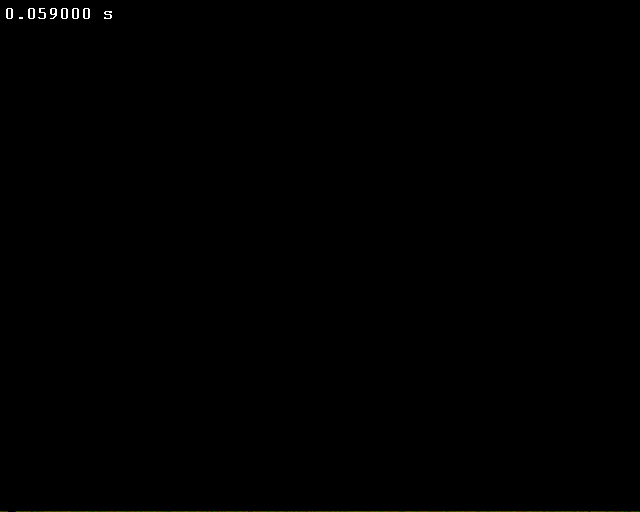

Supplement: S3 File — (ZIP) [file pone.0237709.s003.zip › PEDOT Electrode Recording/Position000059.tif]

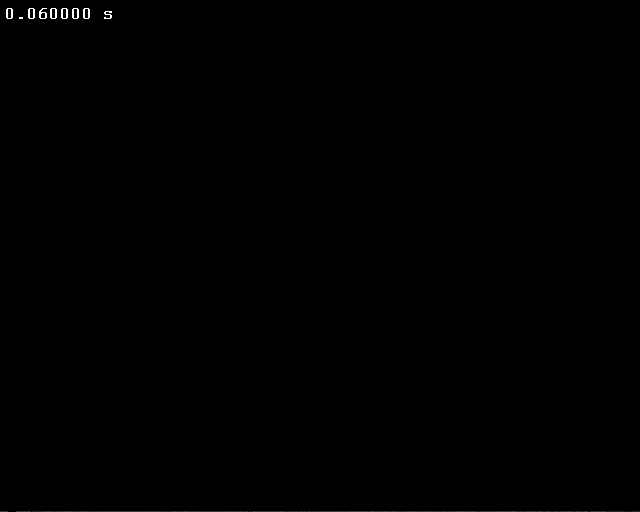

Supplement: S3 File — (ZIP) [file pone.0237709.s003.zip › PEDOT Electrode Recording/Position000060.tif]

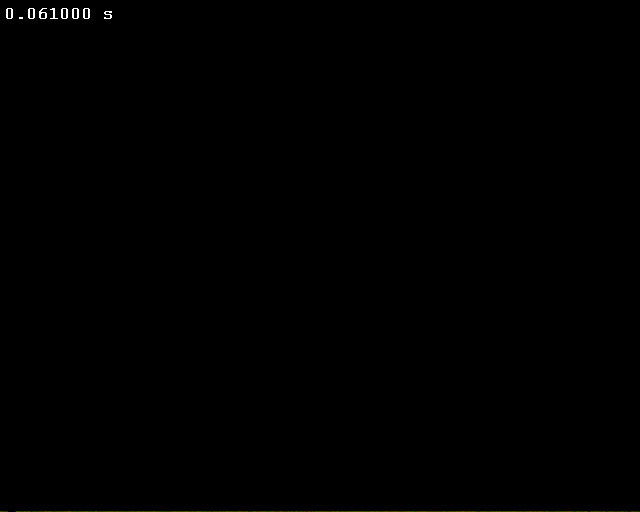

Supplement: S3 File — (ZIP) [file pone.0237709.s003.zip › PEDOT Electrode Recording/Position000061.tif]

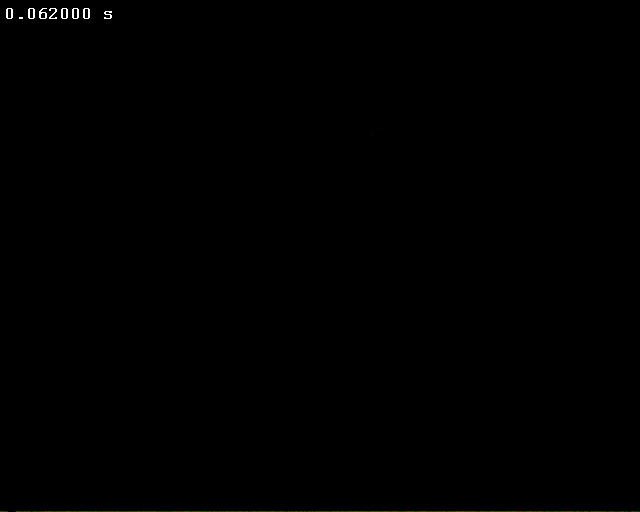

Supplement: S3 File — (ZIP) [file pone.0237709.s003.zip › PEDOT Electrode Recording/Position000062.tif]

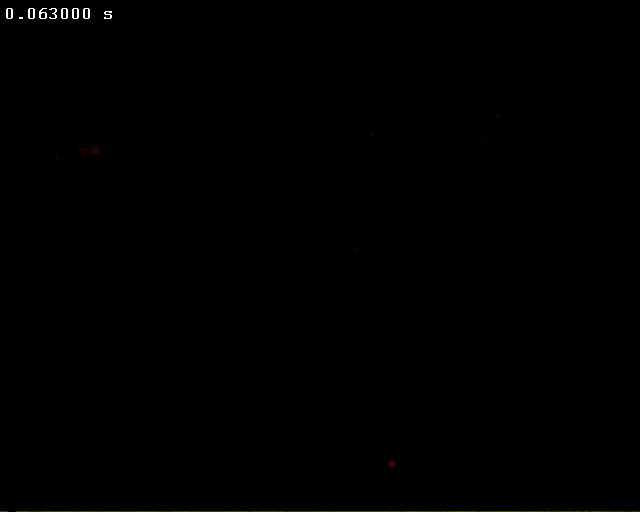

Supplement: S3 File — (ZIP) [file pone.0237709.s003.zip › PEDOT Electrode Recording/Position000063.tif]

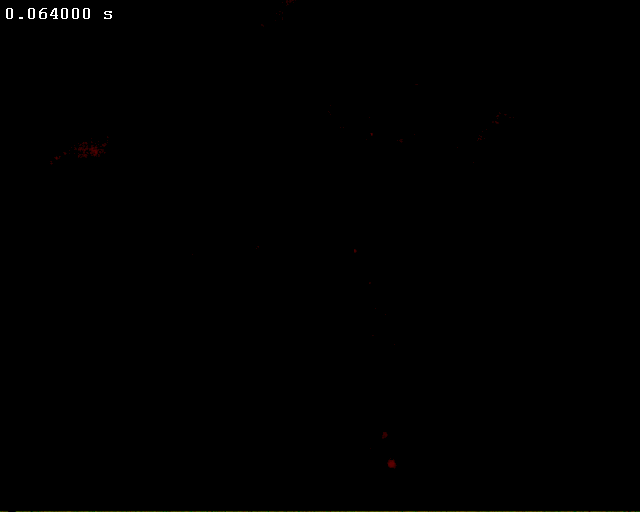

Supplement: S3 File — (ZIP) [file pone.0237709.s003.zip › PEDOT Electrode Recording/Position000064.tif]

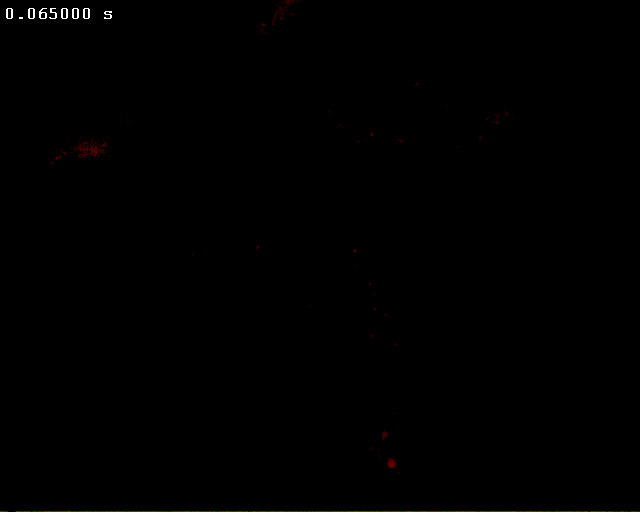

Supplement: S3 File — (ZIP) [file pone.0237709.s003.zip › PEDOT Electrode Recording/Position000065.tif]

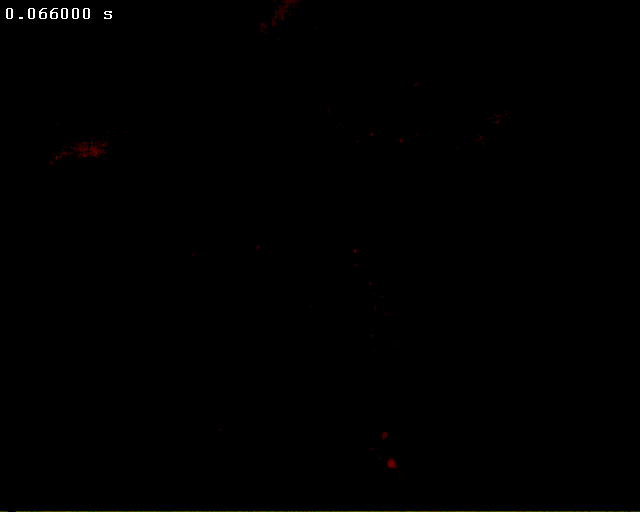

Supplement: S3 File — (ZIP) [file pone.0237709.s003.zip › PEDOT Electrode Recording/Position000066.tif]

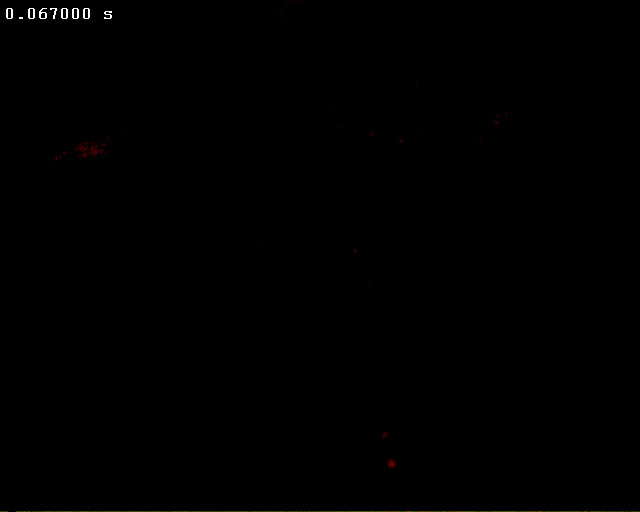

Supplement: S3 File — (ZIP) [file pone.0237709.s003.zip › PEDOT Electrode Recording/Position000067.tif]

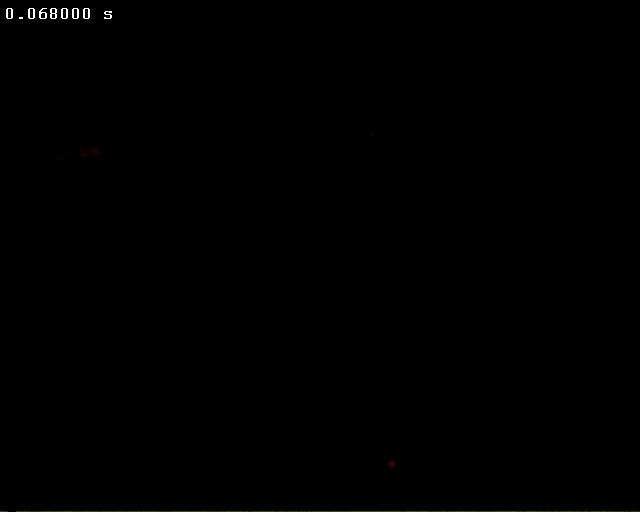

Supplement: S3 File — (ZIP) [file pone.0237709.s003.zip › PEDOT Electrode Recording/Position000068.tif]

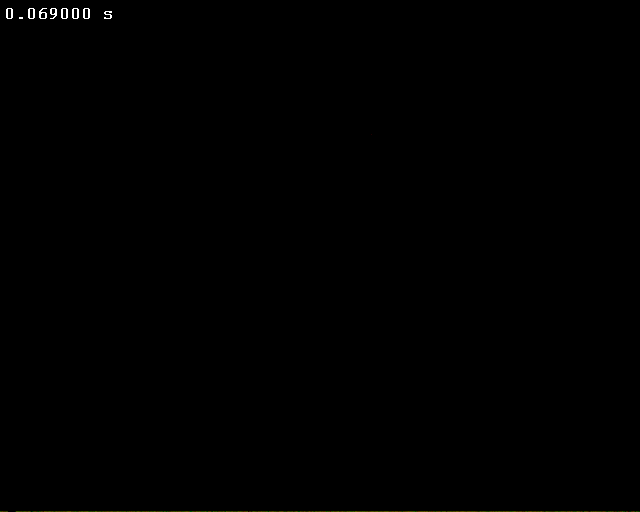

Supplement: S3 File — (ZIP) [file pone.0237709.s003.zip › PEDOT Electrode Recording/Position000069.tif]

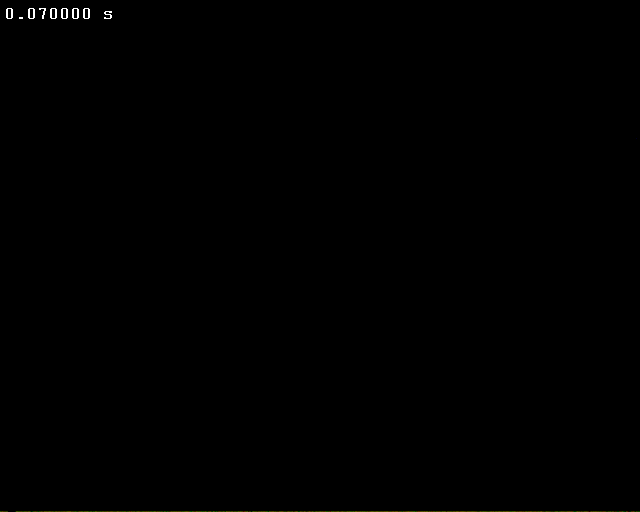

Supplement: S3 File — (ZIP) [file pone.0237709.s003.zip › PEDOT Electrode Recording/Position000070.tif]

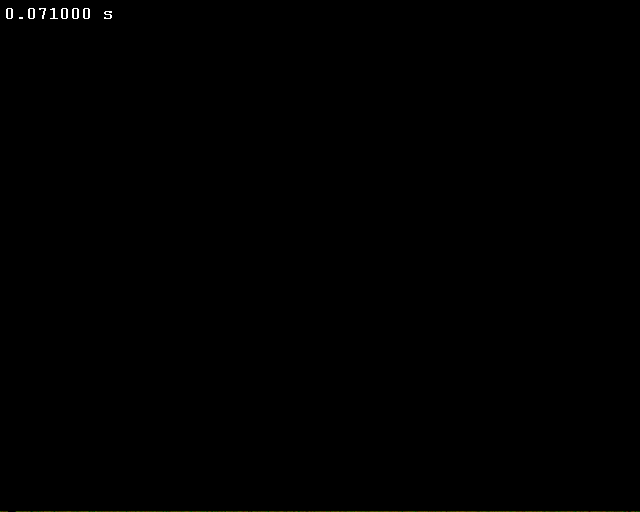

Supplement: S3 File — (ZIP) [file pone.0237709.s003.zip › PEDOT Electrode Recording/Position000071.tif]

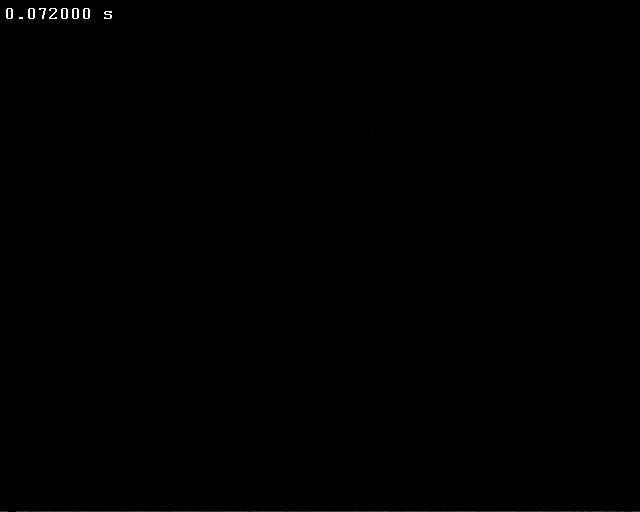

Supplement: S3 File — (ZIP) [file pone.0237709.s003.zip › PEDOT Electrode Recording/Position000072.tif]

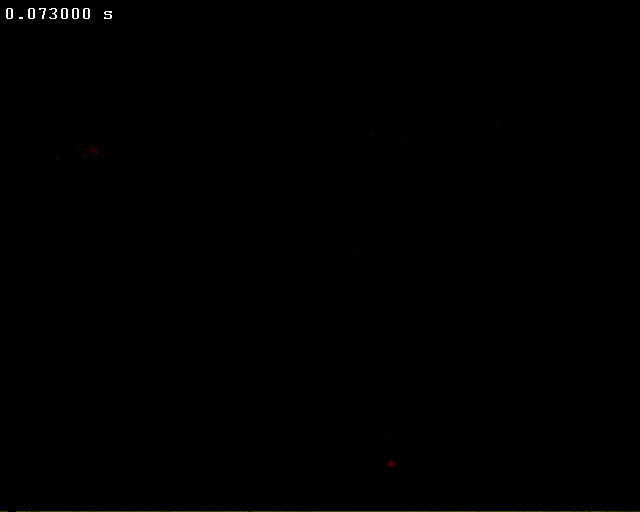

Supplement: S3 File — (ZIP) [file pone.0237709.s003.zip › PEDOT Electrode Recording/Position000073.tif]

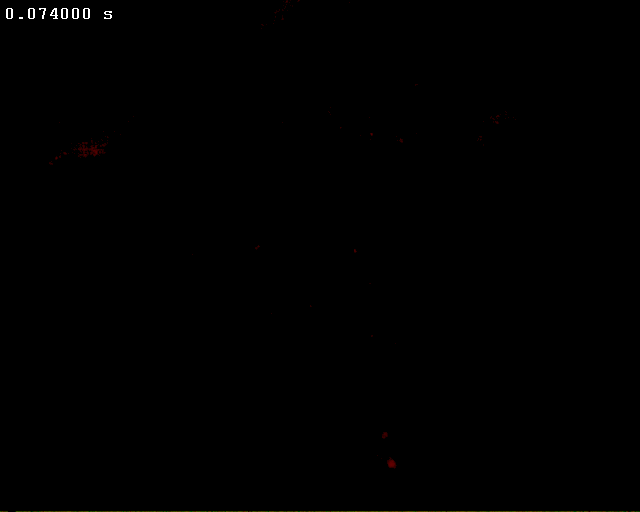

Supplement: S3 File — (ZIP) [file pone.0237709.s003.zip › PEDOT Electrode Recording/Position000074.tif]

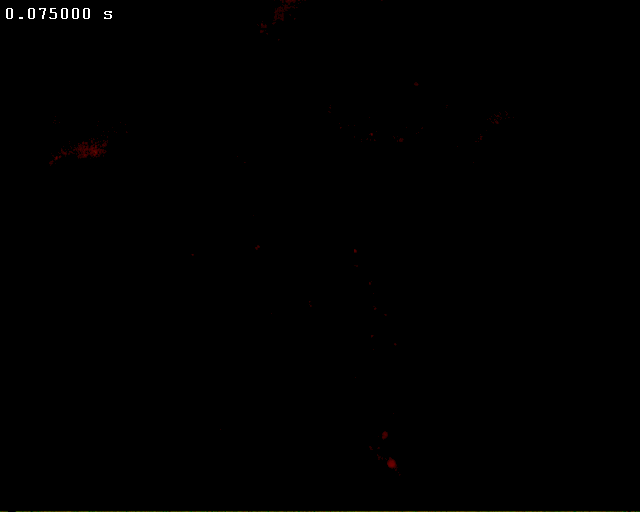

Supplement: S3 File — (ZIP) [file pone.0237709.s003.zip › PEDOT Electrode Recording/Position000075.tif]

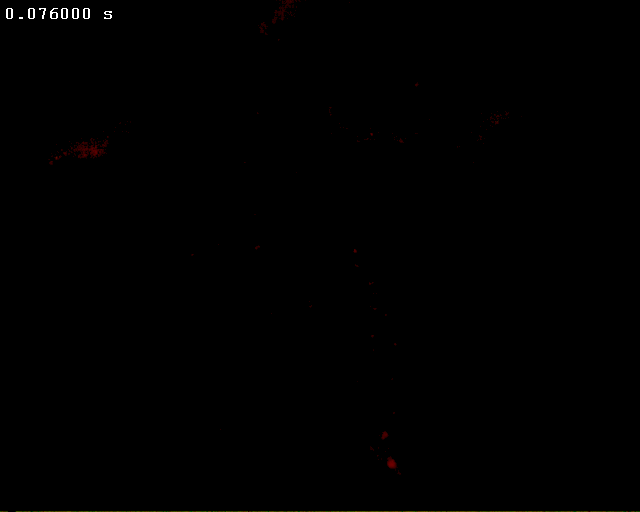

Supplement: S3 File — (ZIP) [file pone.0237709.s003.zip › PEDOT Electrode Recording/Position000076.tif]

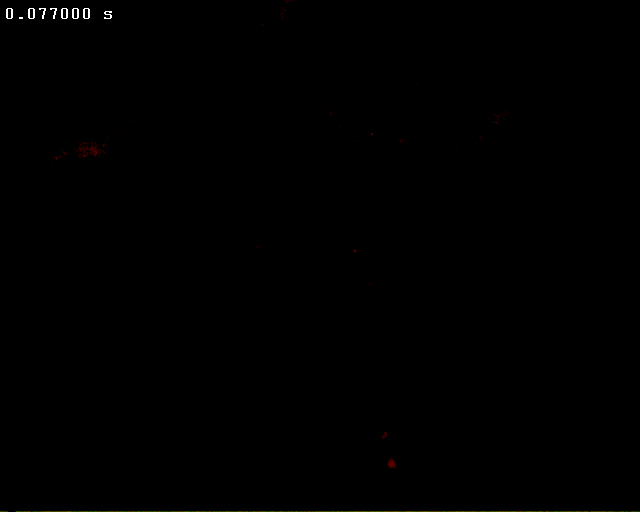

Supplement: S3 File — (ZIP) [file pone.0237709.s003.zip › PEDOT Electrode Recording/Position000077.tif]

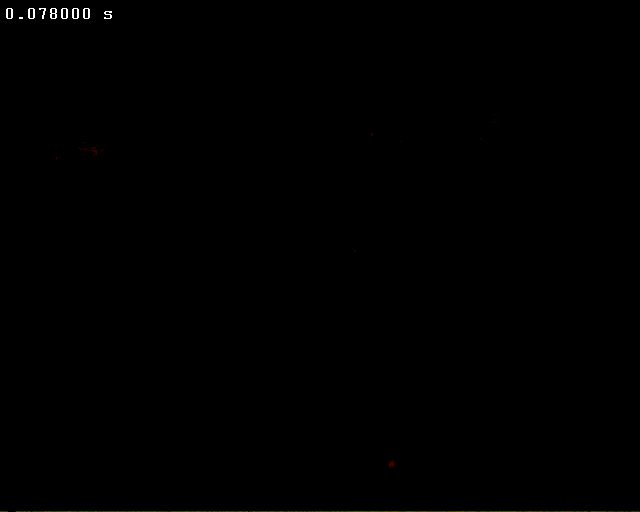

Supplement: S3 File — (ZIP) [file pone.0237709.s003.zip › PEDOT Electrode Recording/Position000078.tif]

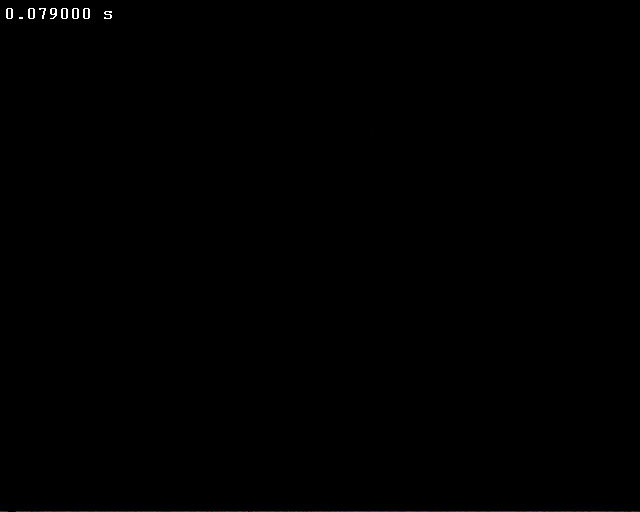

Supplement: S3 File — (ZIP) [file pone.0237709.s003.zip › PEDOT Electrode Recording/Position000079.tif]

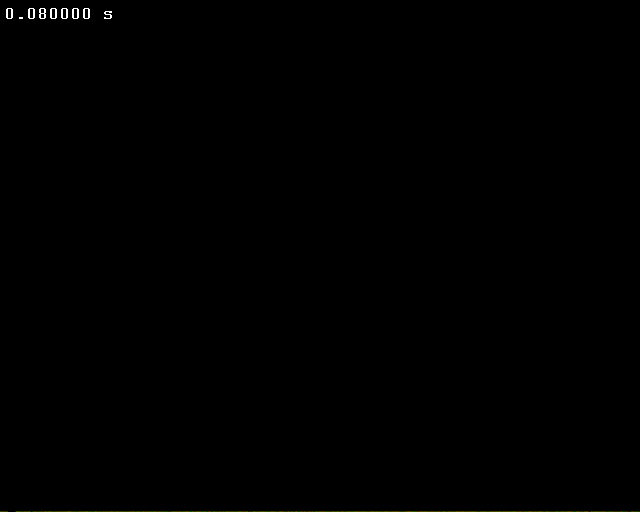

Supplement: S3 File — (ZIP) [file pone.0237709.s003.zip › PEDOT Electrode Recording/Position000080.tif]

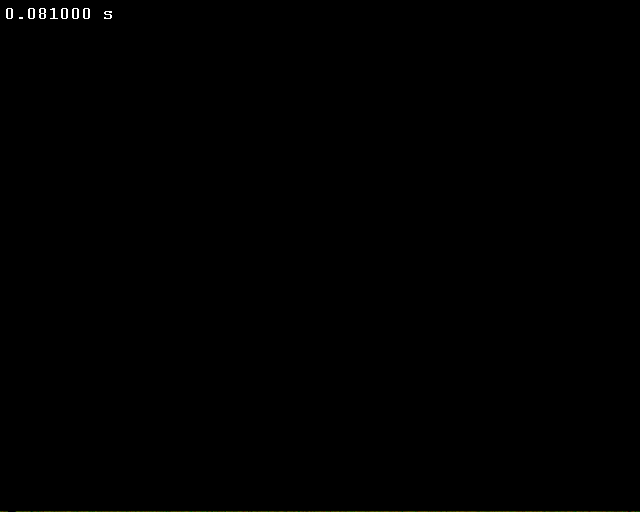

Supplement: S3 File — (ZIP) [file pone.0237709.s003.zip › PEDOT Electrode Recording/Position000081.tif]

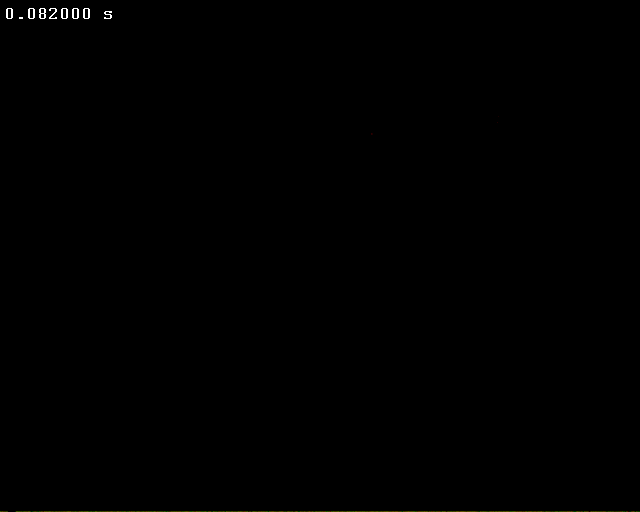

Supplement: S3 File — (ZIP) [file pone.0237709.s003.zip › PEDOT Electrode Recording/Position000082.tif]

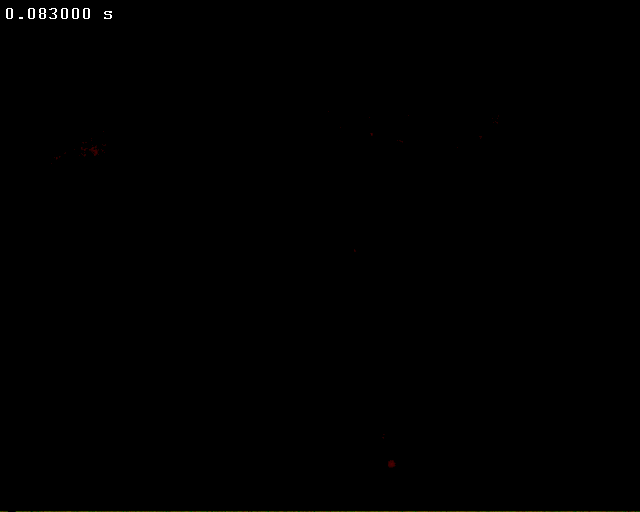

Supplement: S3 File — (ZIP) [file pone.0237709.s003.zip › PEDOT Electrode Recording/Position000083.tif]

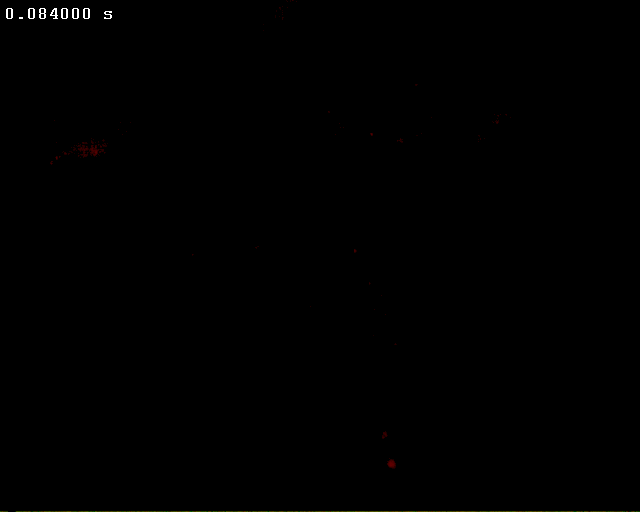

Supplement: S3 File — (ZIP) [file pone.0237709.s003.zip › PEDOT Electrode Recording/Position000084.tif]

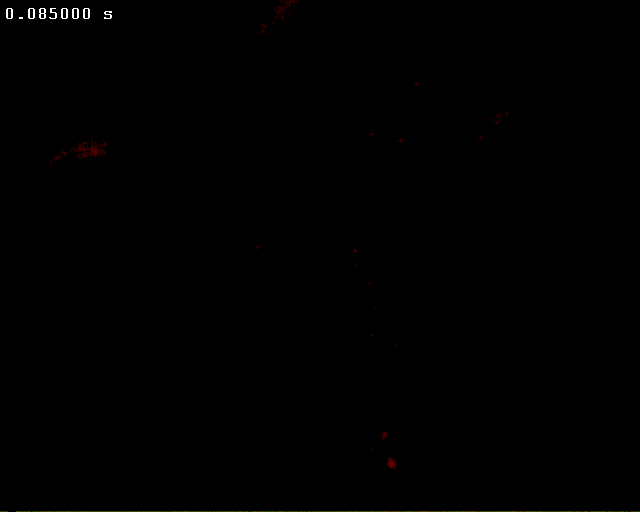

Supplement: S3 File — (ZIP) [file pone.0237709.s003.zip › PEDOT Electrode Recording/Position000085.tif]

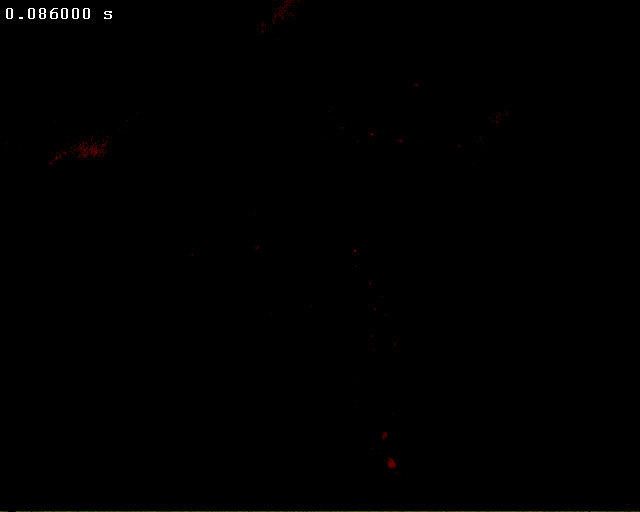

Supplement: S3 File — (ZIP) [file pone.0237709.s003.zip › PEDOT Electrode Recording/Position000086.tif]

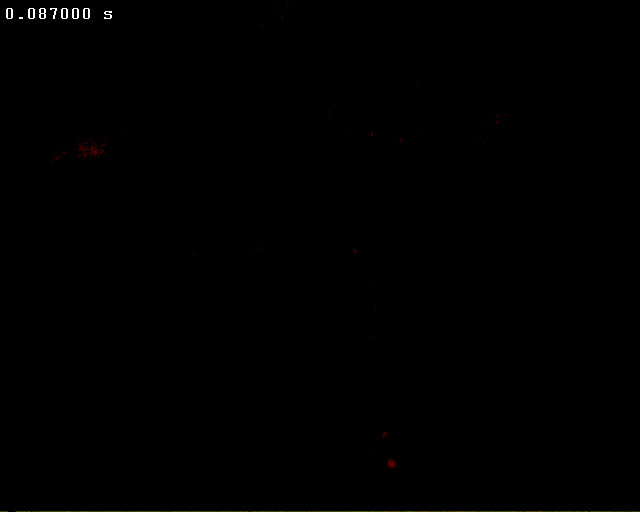

Supplement: S3 File — (ZIP) [file pone.0237709.s003.zip › PEDOT Electrode Recording/Position000087.tif]

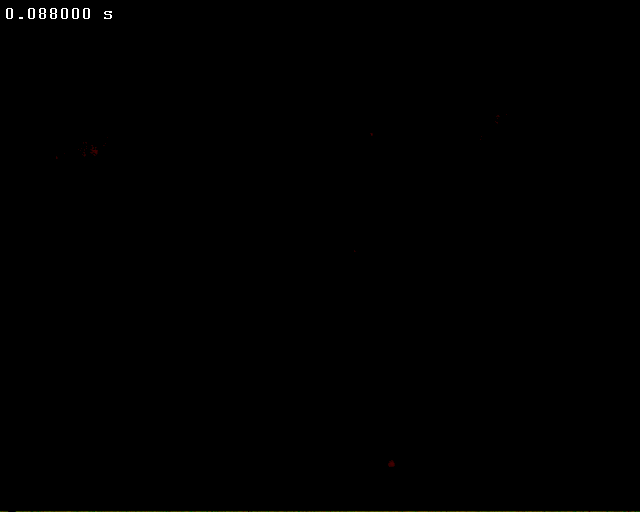

Supplement: S3 File — (ZIP) [file pone.0237709.s003.zip › PEDOT Electrode Recording/Position000088.tif]

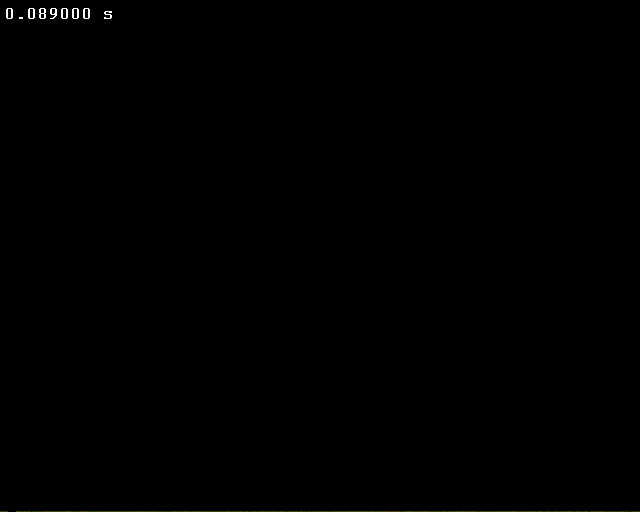

Supplement: S3 File — (ZIP) [file pone.0237709.s003.zip › PEDOT Electrode Recording/Position000089.tif]

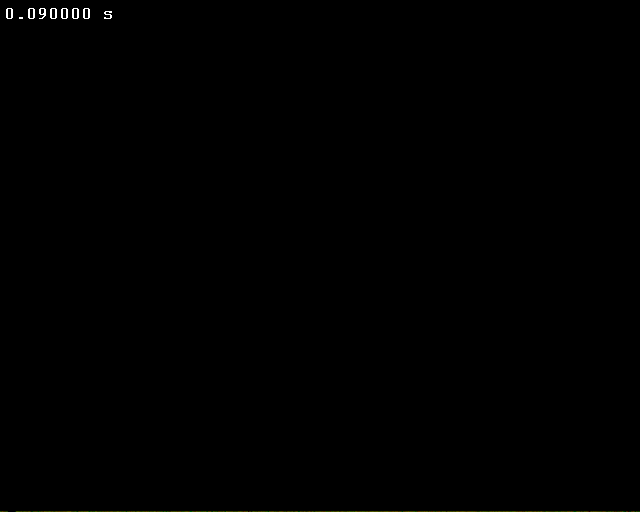

Supplement: S3 File — (ZIP) [file pone.0237709.s003.zip › PEDOT Electrode Recording/Position000090.tif]

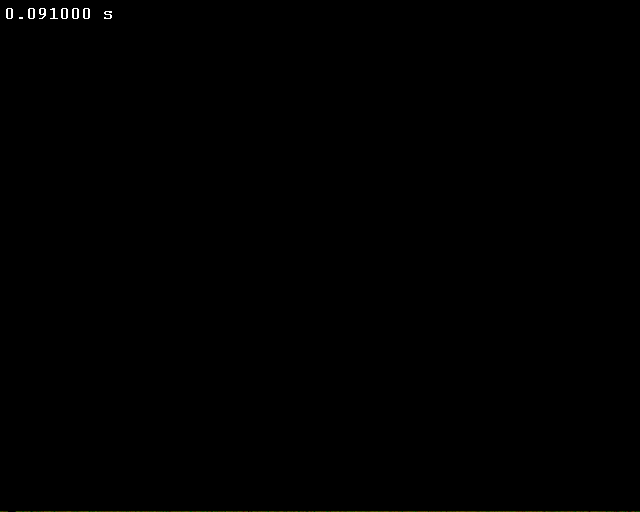

Supplement: S3 File — (ZIP) [file pone.0237709.s003.zip › PEDOT Electrode Recording/Position000091.tif]

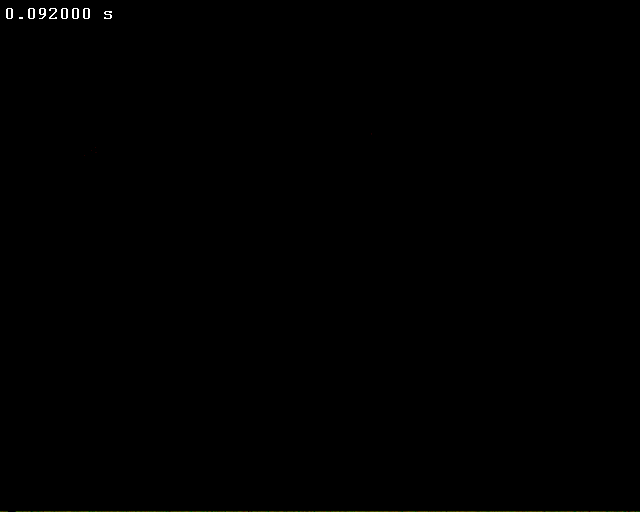

Supplement: S3 File — (ZIP) [file pone.0237709.s003.zip › PEDOT Electrode Recording/Position000092.tif]

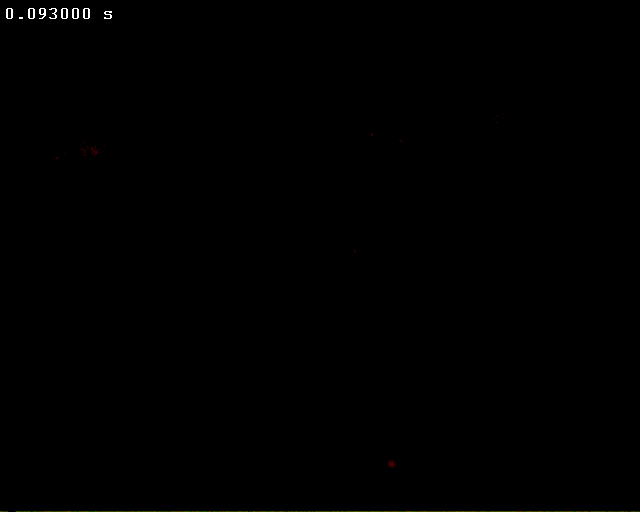

Supplement: S3 File — (ZIP) [file pone.0237709.s003.zip › PEDOT Electrode Recording/Position000093.tif]

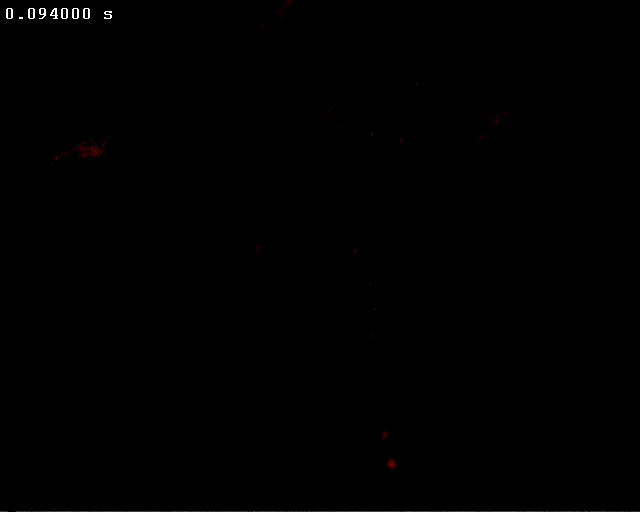

Supplement: S3 File — (ZIP) [file pone.0237709.s003.zip › PEDOT Electrode Recording/Position000094.tif]

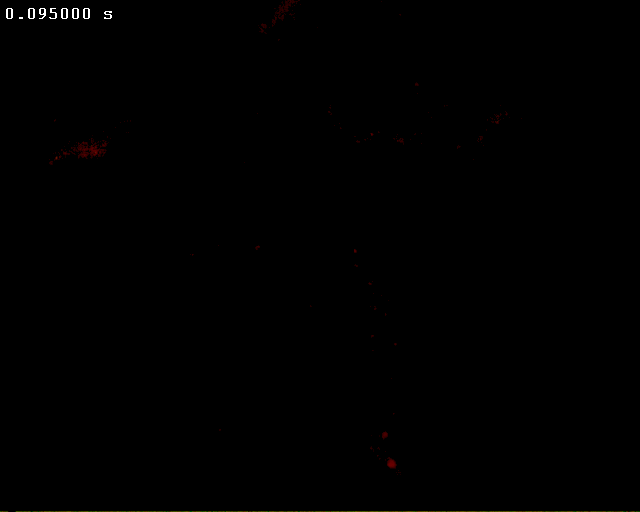

Supplement: S3 File — (ZIP) [file pone.0237709.s003.zip › PEDOT Electrode Recording/Position000095.tif]

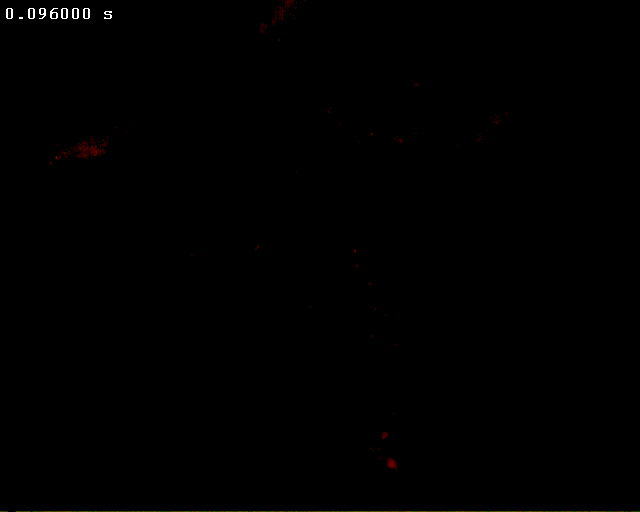

Supplement: S3 File — (ZIP) [file pone.0237709.s003.zip › PEDOT Electrode Recording/Position000096.tif]

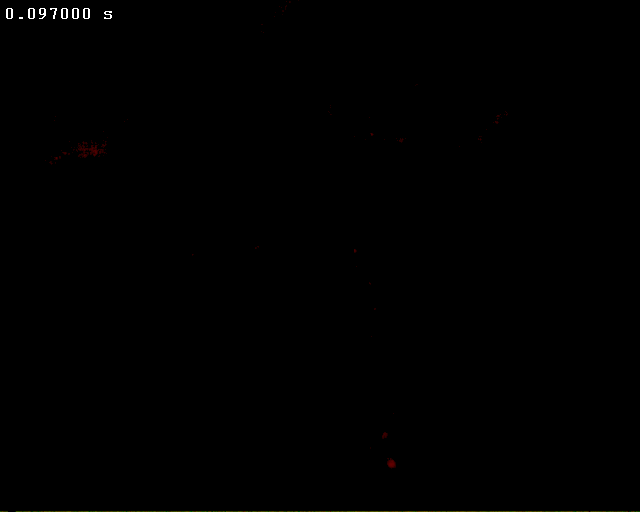

Supplement: S3 File — (ZIP) [file pone.0237709.s003.zip › PEDOT Electrode Recording/Position000097.tif]

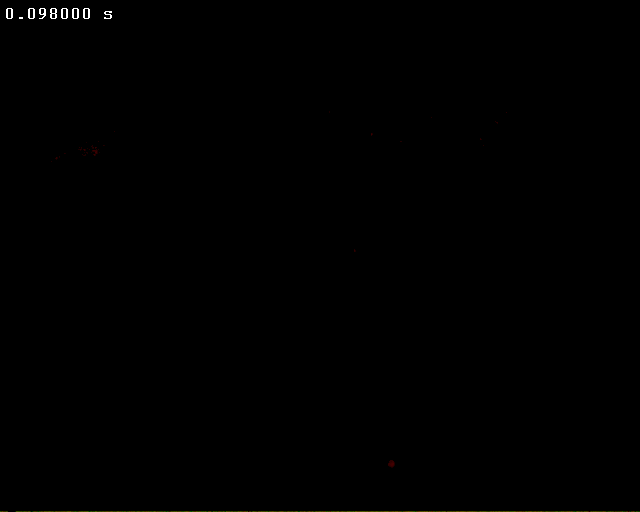

Supplement: S3 File — (ZIP) [file pone.0237709.s003.zip › PEDOT Electrode Recording/Position000098.tif]
