# Supplementary figures and images for: A simultaneous optical and electrical in-vitro neuronal recording system to evaluate microelectrode performance (part 2 of 2)
Source: PLoS One. 2020 Aug 20;15(8):e0237709. doi: 10.1371/journal.pone.0237709 (PMC7440637; doi:10.1371/journal.pone.0237709)

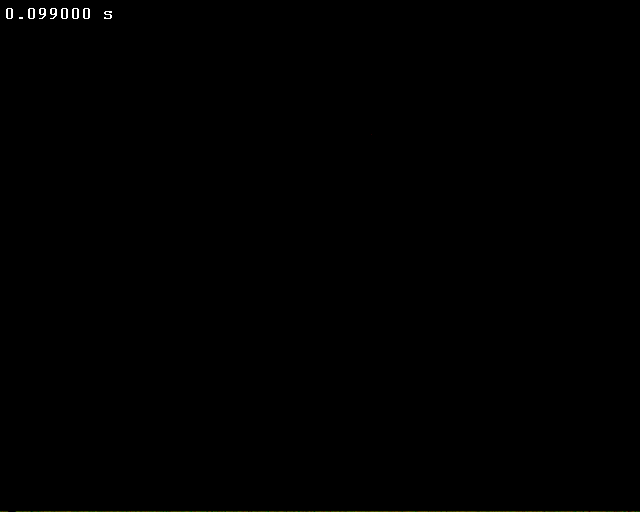

Supplement: S3 File — (ZIP) [file pone.0237709.s003.zip › PEDOT Electrode Recording/Position000099.tif]

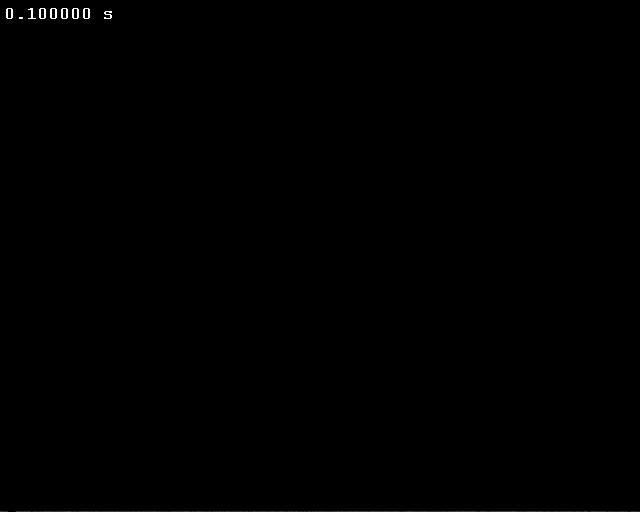

Supplement: S3 File — (ZIP) [file pone.0237709.s003.zip › PEDOT Electrode Recording/Position000100.tif]

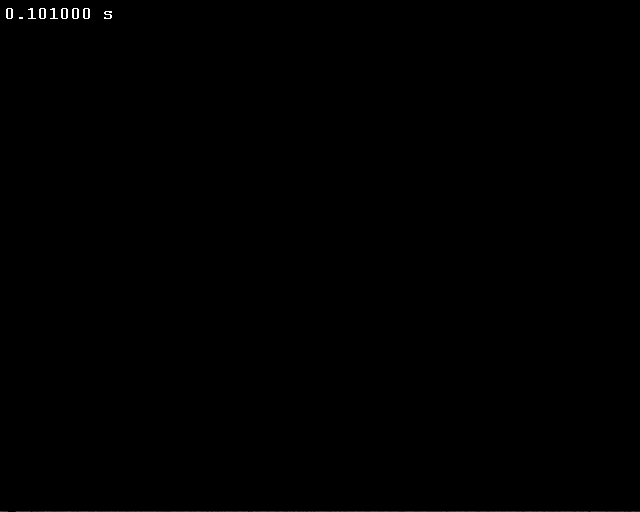

Supplement: S3 File — (ZIP) [file pone.0237709.s003.zip › PEDOT Electrode Recording/Position000101.tif]

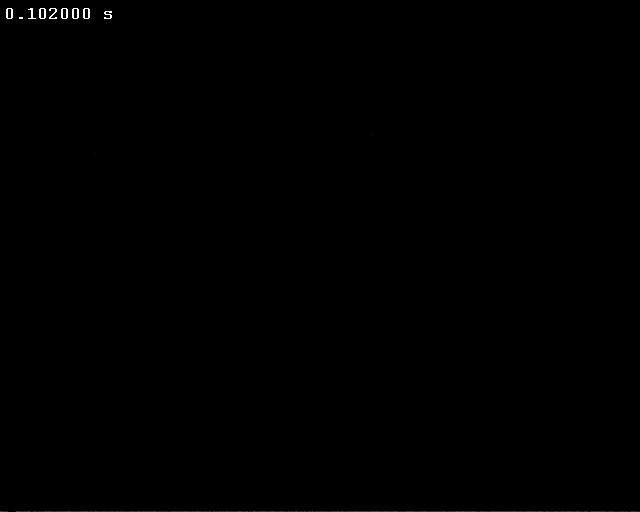

Supplement: S3 File — (ZIP) [file pone.0237709.s003.zip › PEDOT Electrode Recording/Position000102.tif]

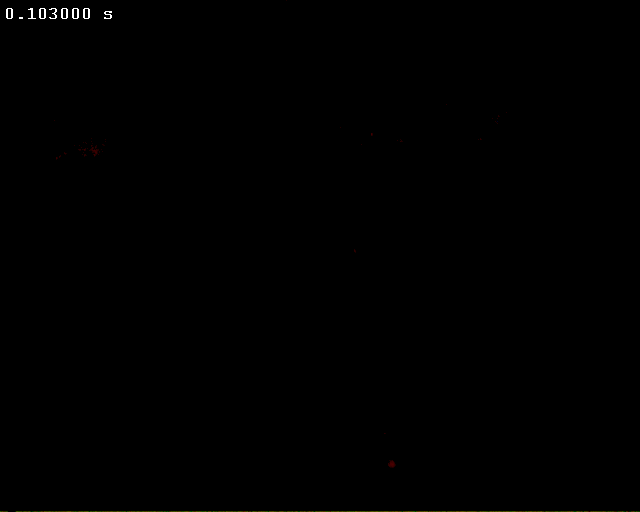

Supplement: S3 File — (ZIP) [file pone.0237709.s003.zip › PEDOT Electrode Recording/Position000103.tif]

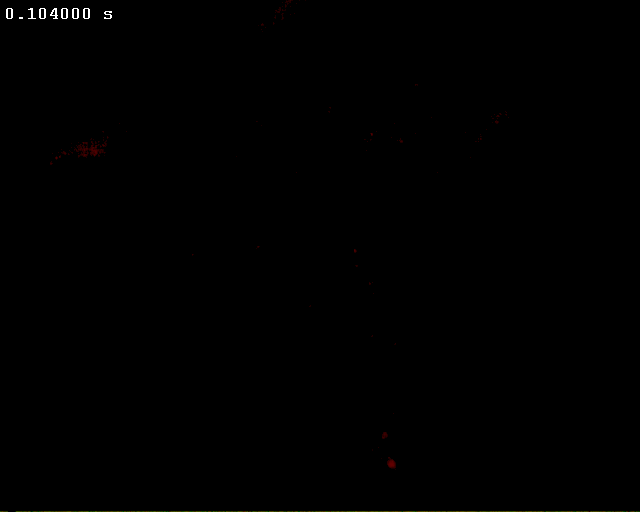

Supplement: S3 File — (ZIP) [file pone.0237709.s003.zip › PEDOT Electrode Recording/Position000104.tif]

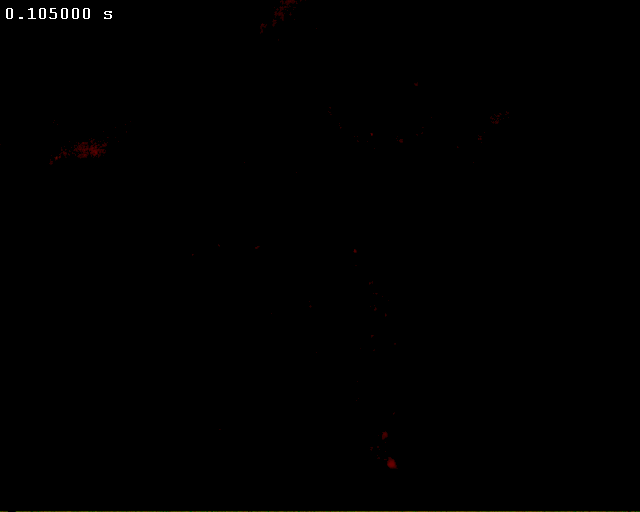

Supplement: S3 File — (ZIP) [file pone.0237709.s003.zip › PEDOT Electrode Recording/Position000105.tif]

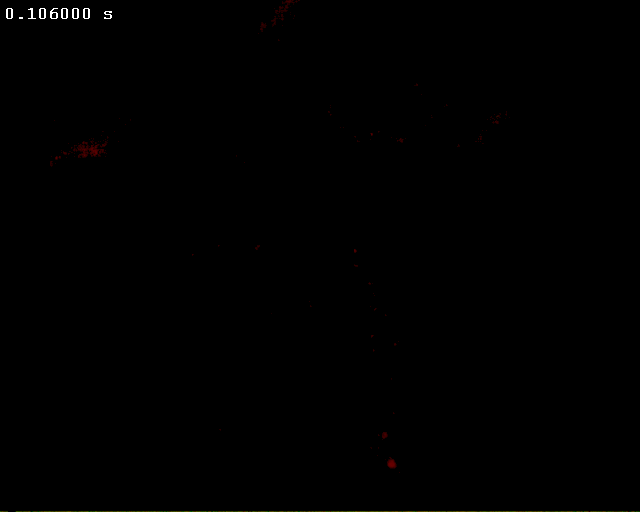

Supplement: S3 File — (ZIP) [file pone.0237709.s003.zip › PEDOT Electrode Recording/Position000106.tif]

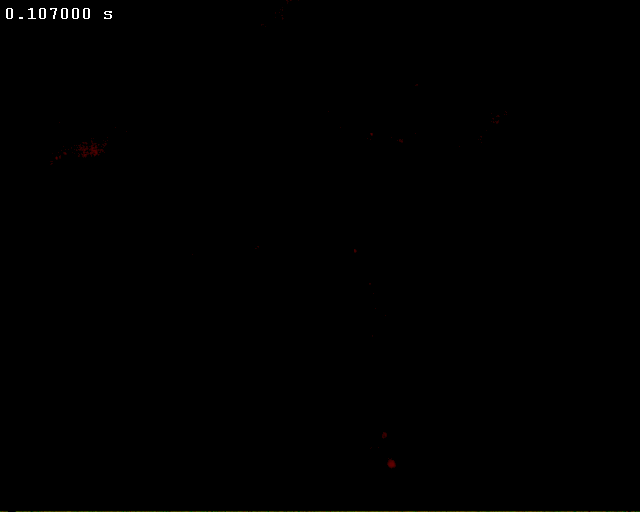

Supplement: S3 File — (ZIP) [file pone.0237709.s003.zip › PEDOT Electrode Recording/Position000107.tif]

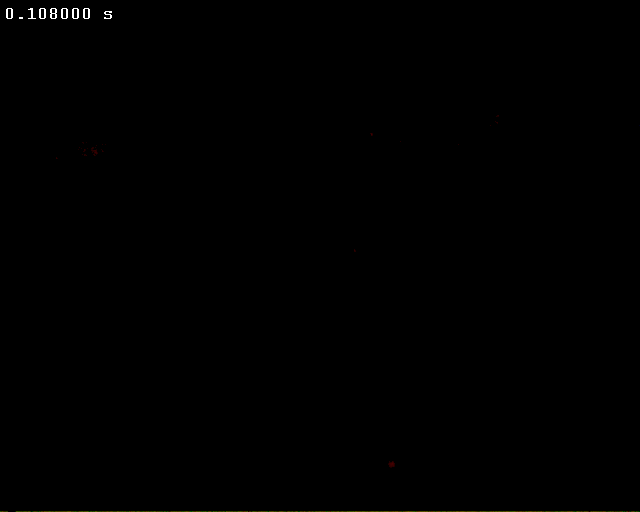

Supplement: S3 File — (ZIP) [file pone.0237709.s003.zip › PEDOT Electrode Recording/Position000108.tif]

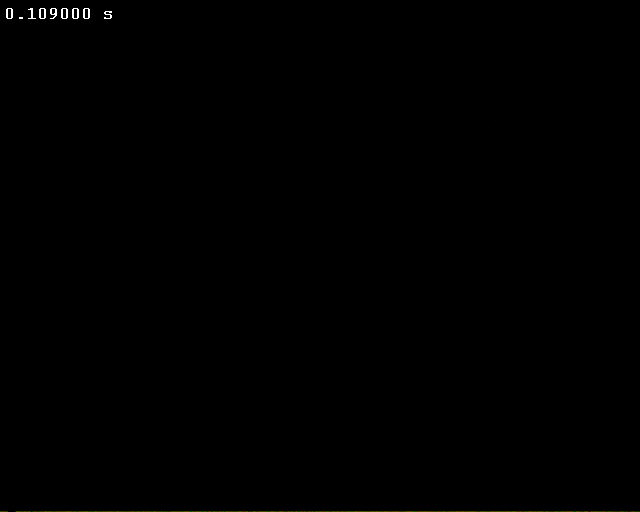

Supplement: S3 File — (ZIP) [file pone.0237709.s003.zip › PEDOT Electrode Recording/Position000109.tif]

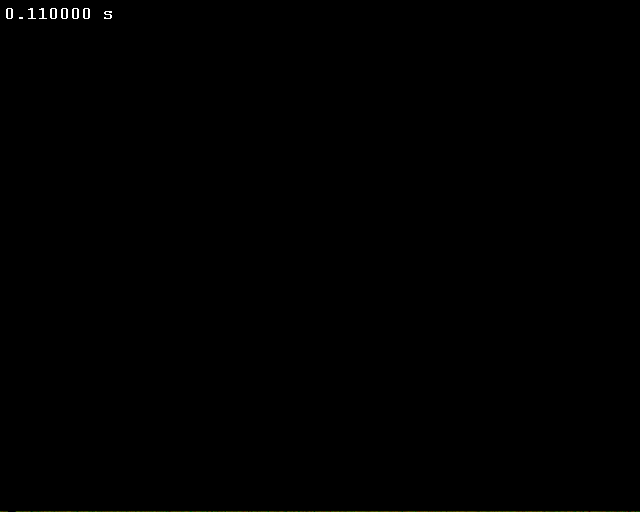

Supplement: S3 File — (ZIP) [file pone.0237709.s003.zip › PEDOT Electrode Recording/Position000110.tif]

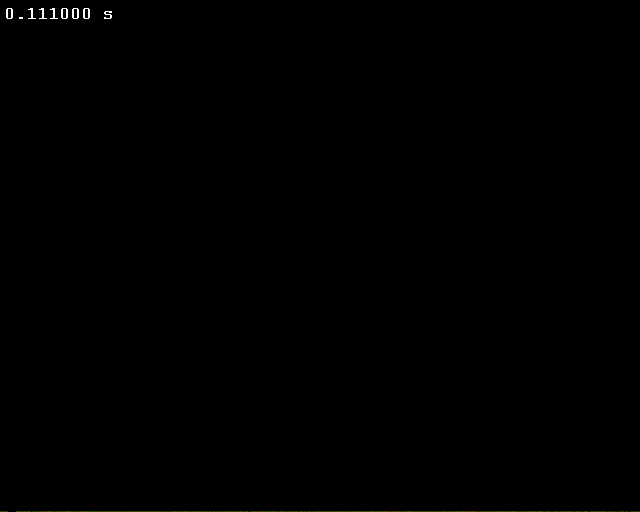

Supplement: S3 File — (ZIP) [file pone.0237709.s003.zip › PEDOT Electrode Recording/Position000111.tif]

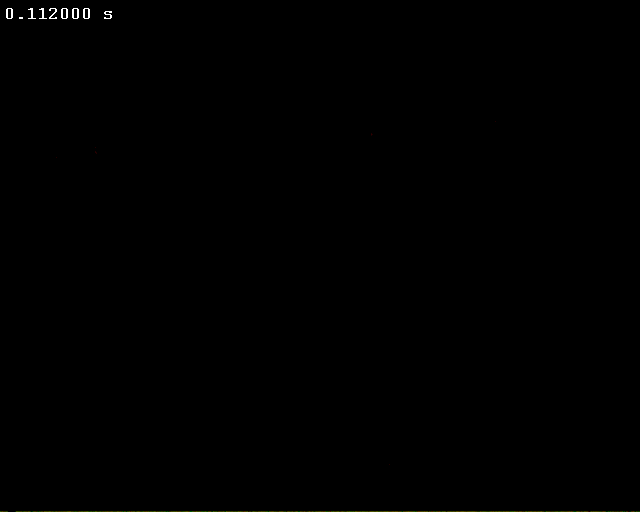

Supplement: S3 File — (ZIP) [file pone.0237709.s003.zip › PEDOT Electrode Recording/Position000112.tif]

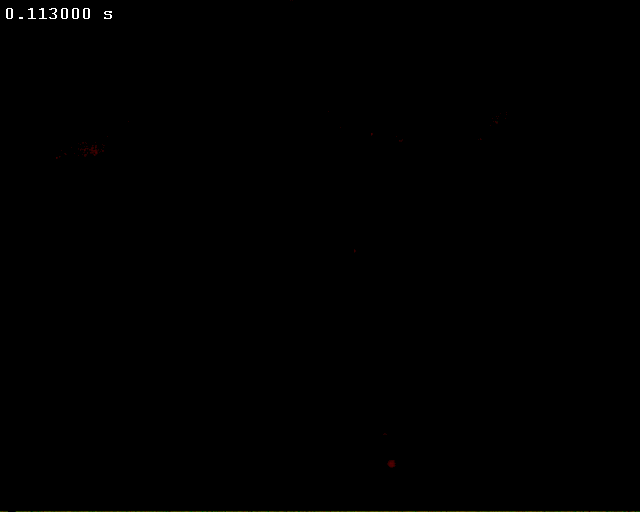

Supplement: S3 File — (ZIP) [file pone.0237709.s003.zip › PEDOT Electrode Recording/Position000113.tif]

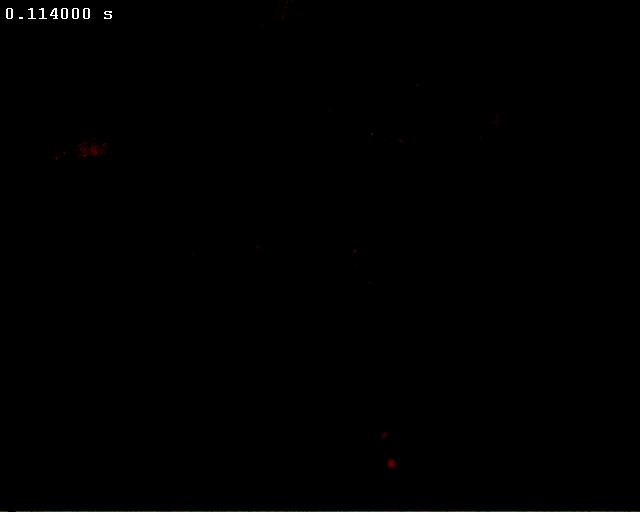

Supplement: S3 File — (ZIP) [file pone.0237709.s003.zip › PEDOT Electrode Recording/Position000114.tif]

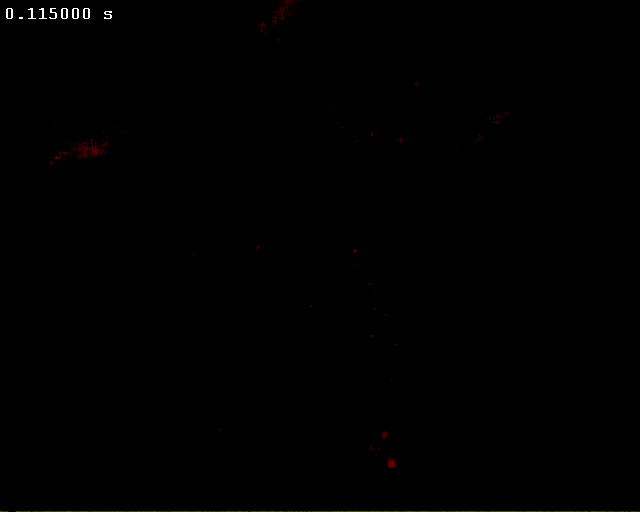

Supplement: S3 File — (ZIP) [file pone.0237709.s003.zip › PEDOT Electrode Recording/Position000115.tif]

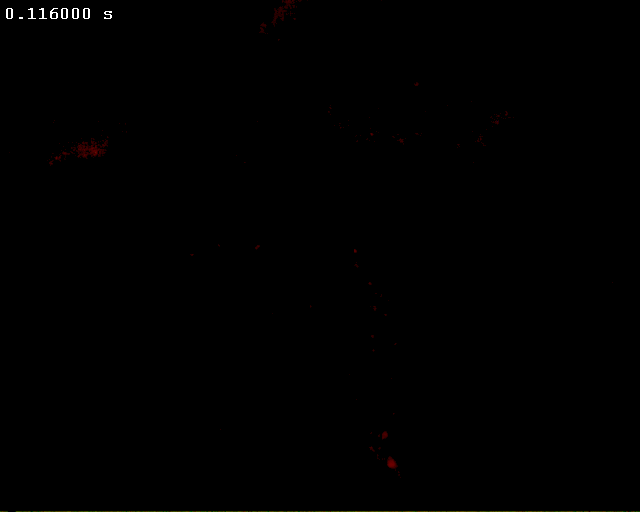

Supplement: S3 File — (ZIP) [file pone.0237709.s003.zip › PEDOT Electrode Recording/Position000116.tif]

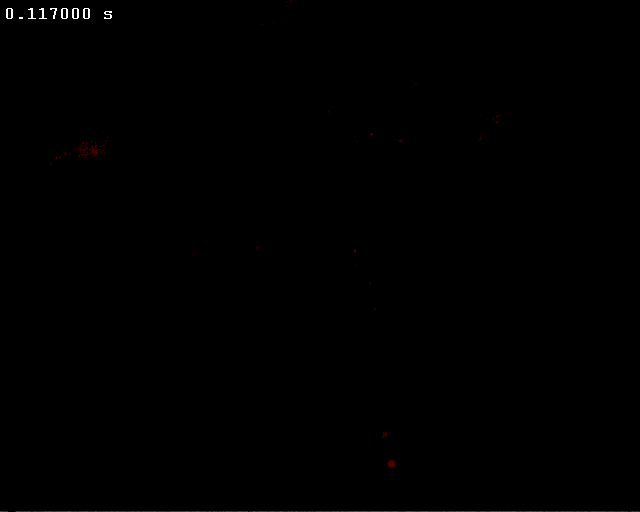

Supplement: S3 File — (ZIP) [file pone.0237709.s003.zip › PEDOT Electrode Recording/Position000117.tif]

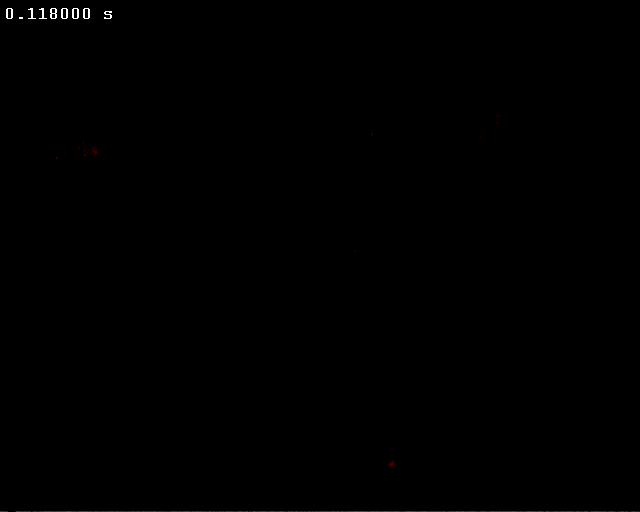

Supplement: S3 File — (ZIP) [file pone.0237709.s003.zip › PEDOT Electrode Recording/Position000118.tif]

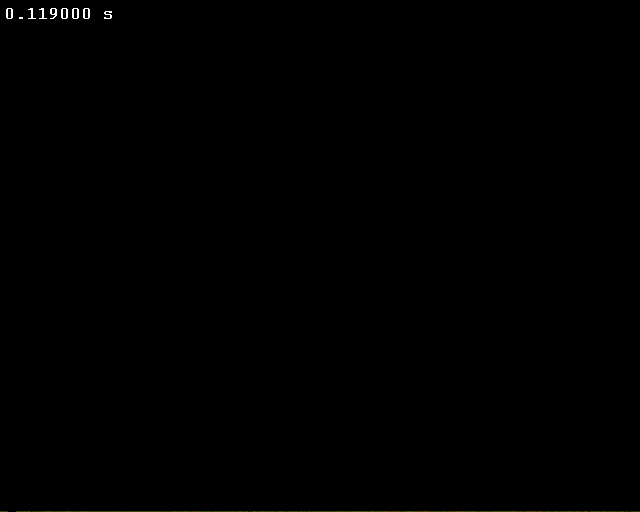

Supplement: S3 File — (ZIP) [file pone.0237709.s003.zip › PEDOT Electrode Recording/Position000119.tif]

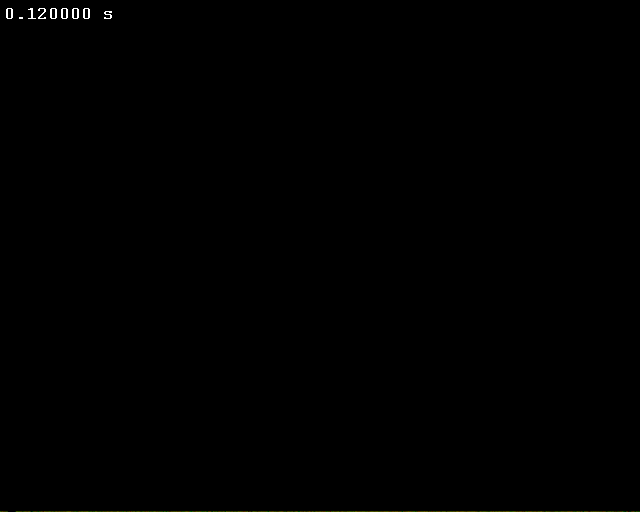

Supplement: S3 File — (ZIP) [file pone.0237709.s003.zip › PEDOT Electrode Recording/Position000120.tif]

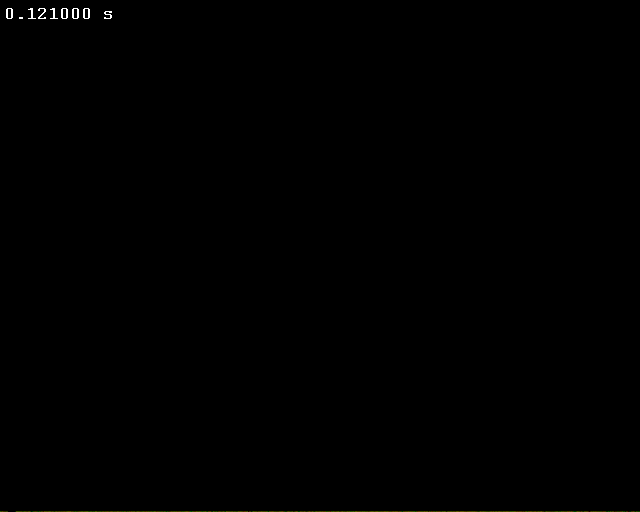

Supplement: S3 File — (ZIP) [file pone.0237709.s003.zip › PEDOT Electrode Recording/Position000121.tif]

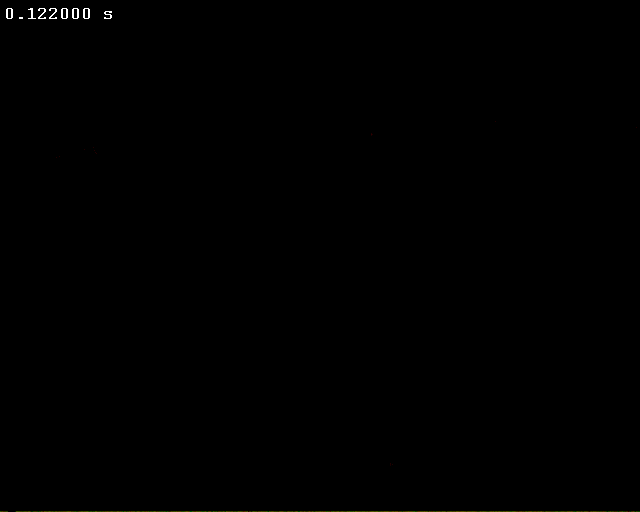

Supplement: S3 File — (ZIP) [file pone.0237709.s003.zip › PEDOT Electrode Recording/Position000122.tif]

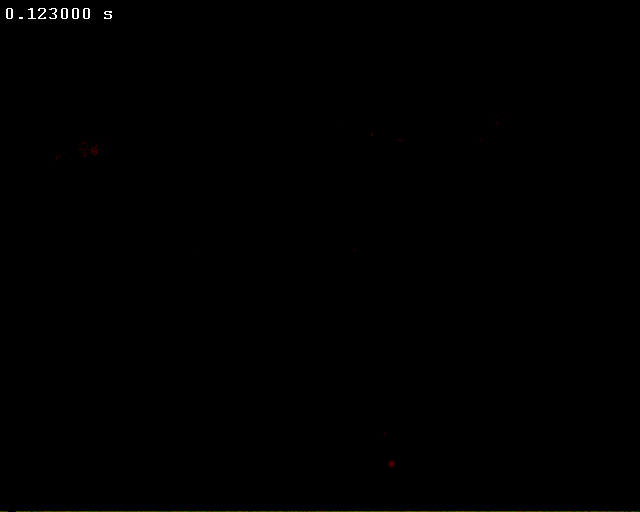

Supplement: S3 File — (ZIP) [file pone.0237709.s003.zip › PEDOT Electrode Recording/Position000123.tif]

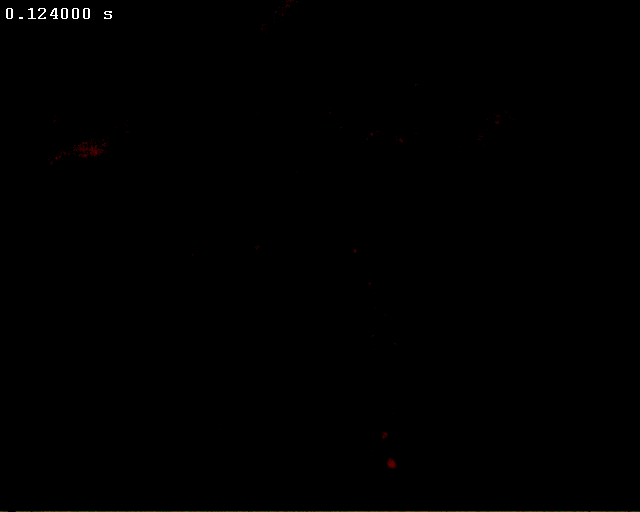

Supplement: S3 File — (ZIP) [file pone.0237709.s003.zip › PEDOT Electrode Recording/Position000124.tif]

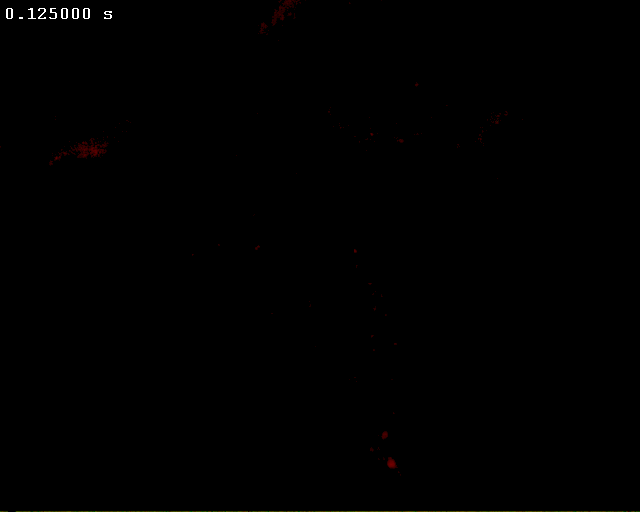

Supplement: S3 File — (ZIP) [file pone.0237709.s003.zip › PEDOT Electrode Recording/Position000125.tif]

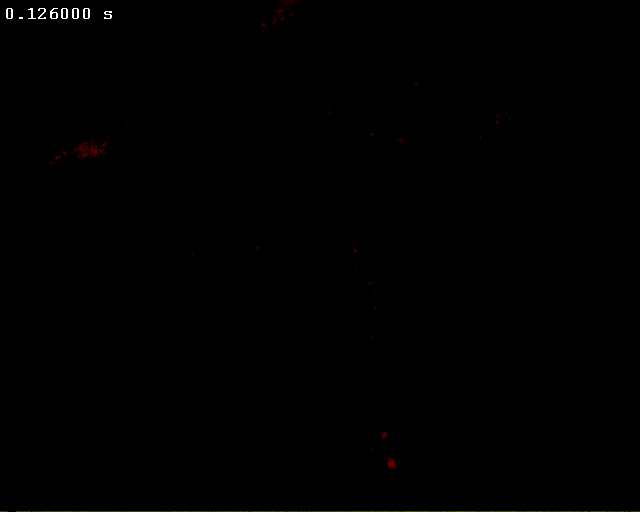

Supplement: S3 File — (ZIP) [file pone.0237709.s003.zip › PEDOT Electrode Recording/Position000126.tif]

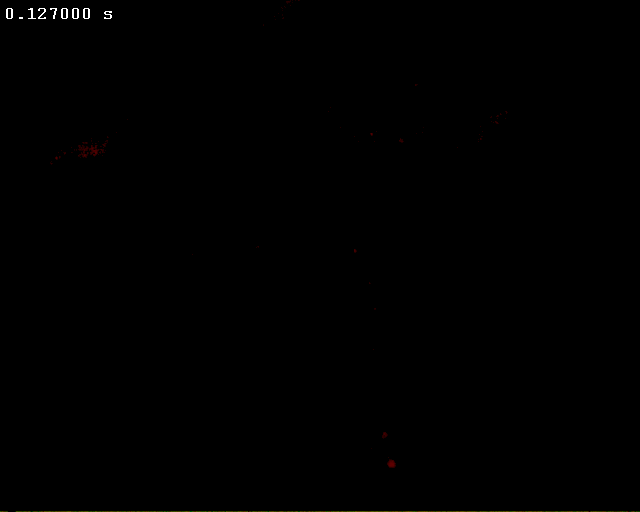

Supplement: S3 File — (ZIP) [file pone.0237709.s003.zip › PEDOT Electrode Recording/Position000127.tif]

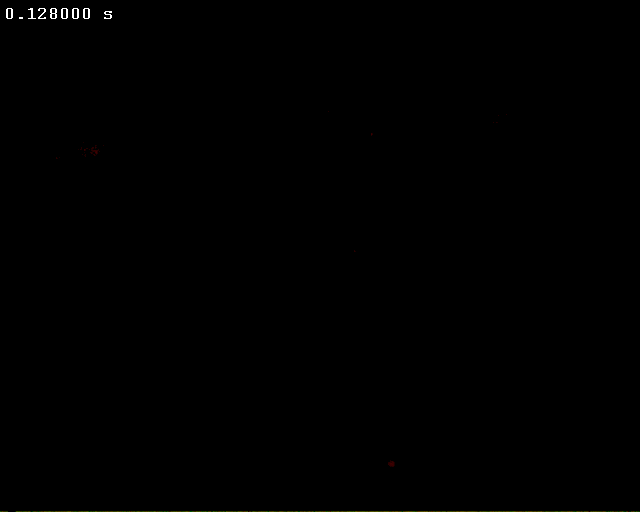

Supplement: S3 File — (ZIP) [file pone.0237709.s003.zip › PEDOT Electrode Recording/Position000128.tif]

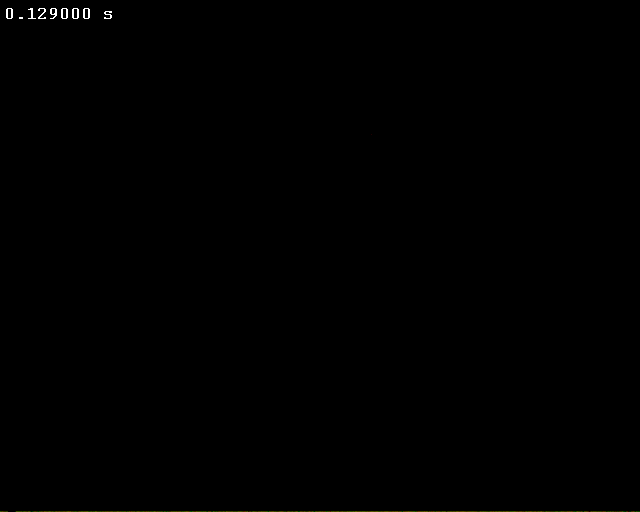

Supplement: S3 File — (ZIP) [file pone.0237709.s003.zip › PEDOT Electrode Recording/Position000129.tif]

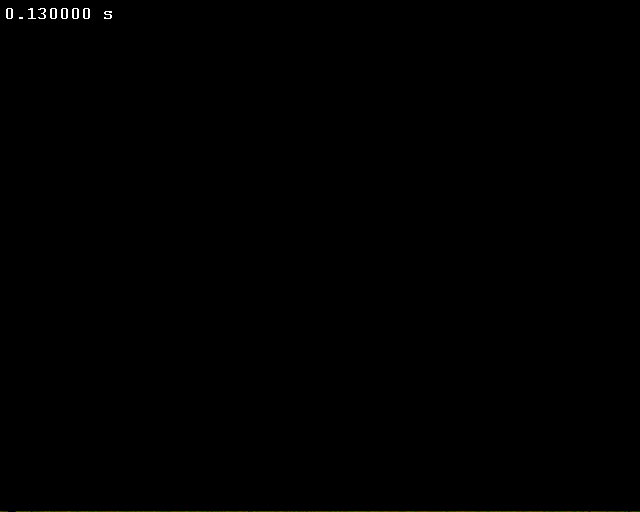

Supplement: S3 File — (ZIP) [file pone.0237709.s003.zip › PEDOT Electrode Recording/Position000130.tif]

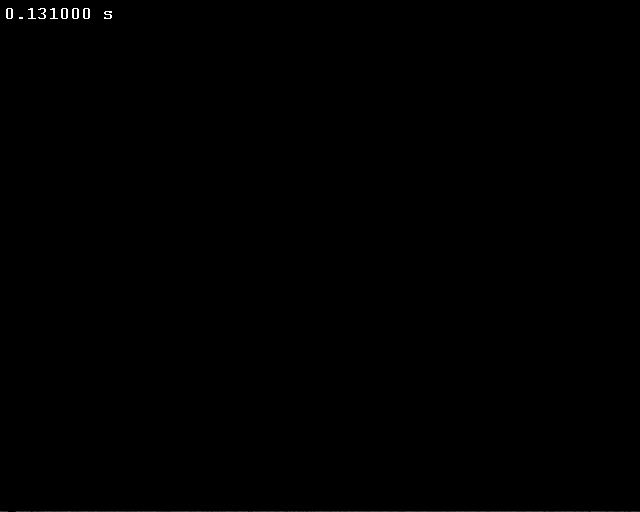

Supplement: S3 File — (ZIP) [file pone.0237709.s003.zip › PEDOT Electrode Recording/Position000131.tif]

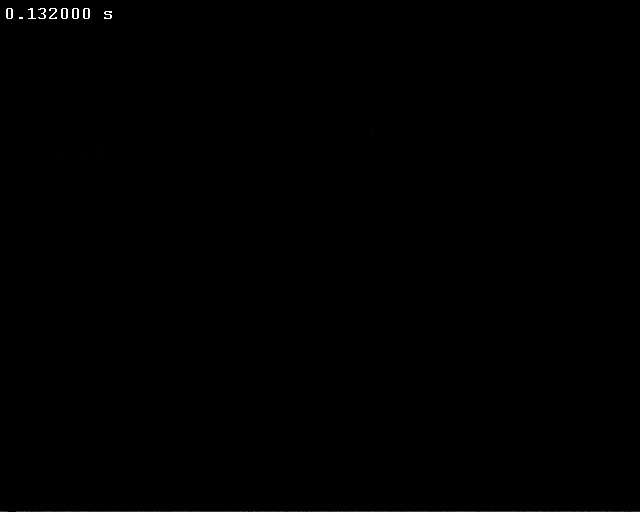

Supplement: S3 File — (ZIP) [file pone.0237709.s003.zip › PEDOT Electrode Recording/Position000132.tif]

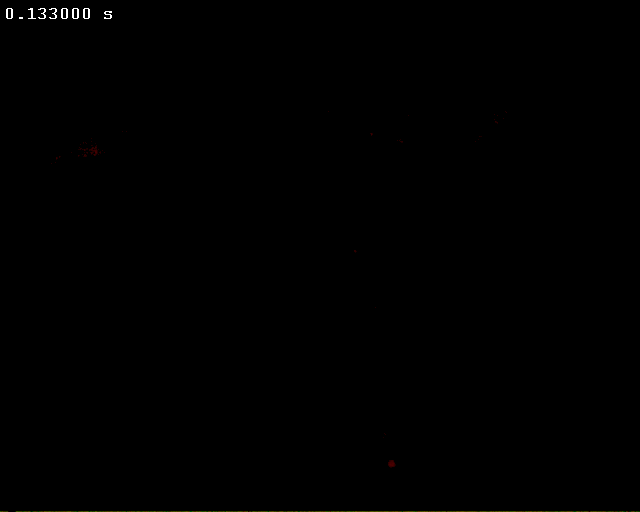

Supplement: S3 File — (ZIP) [file pone.0237709.s003.zip › PEDOT Electrode Recording/Position000133.tif]

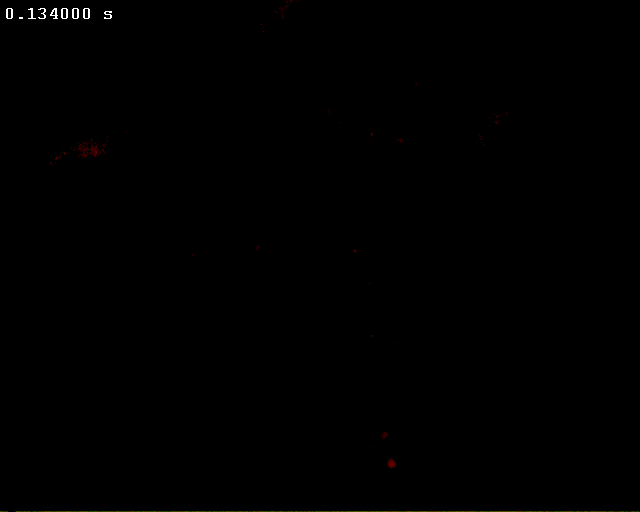

Supplement: S3 File — (ZIP) [file pone.0237709.s003.zip › PEDOT Electrode Recording/Position000134.tif]

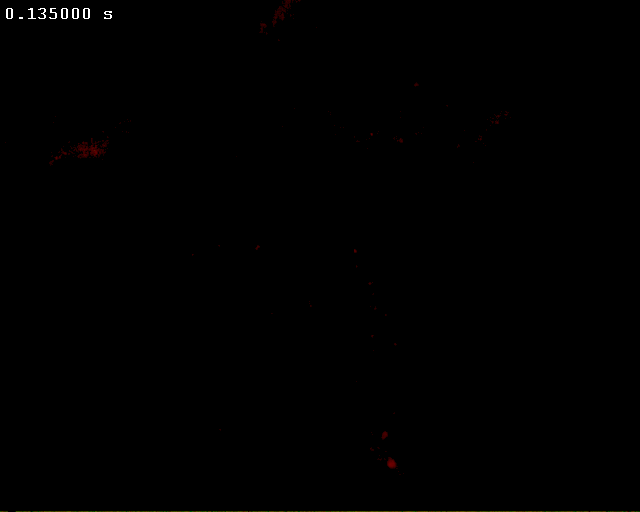

Supplement: S3 File — (ZIP) [file pone.0237709.s003.zip › PEDOT Electrode Recording/Position000135.tif]

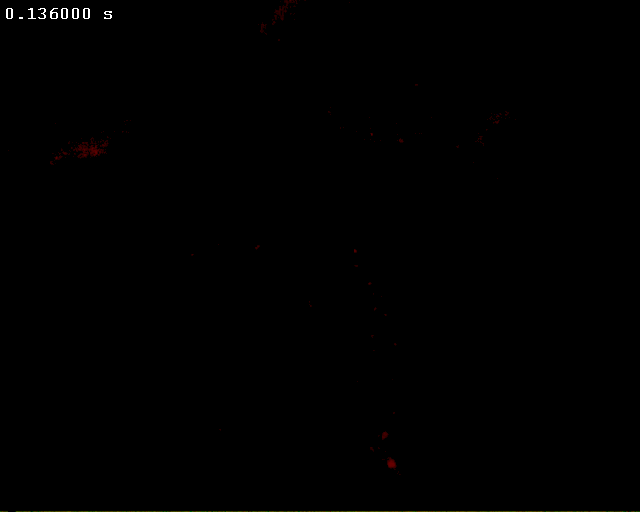

Supplement: S3 File — (ZIP) [file pone.0237709.s003.zip › PEDOT Electrode Recording/Position000136.tif]

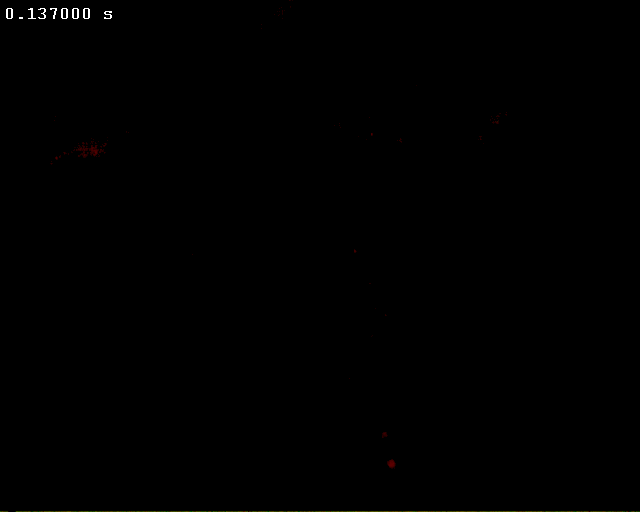

Supplement: S3 File — (ZIP) [file pone.0237709.s003.zip › PEDOT Electrode Recording/Position000137.tif]

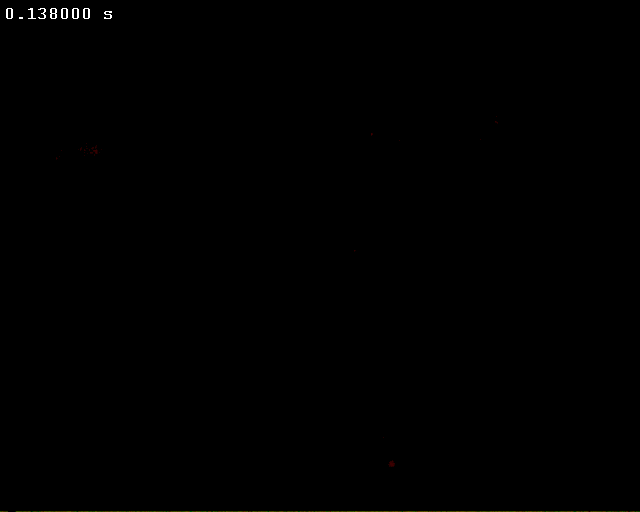

Supplement: S3 File — (ZIP) [file pone.0237709.s003.zip › PEDOT Electrode Recording/Position000138.tif]

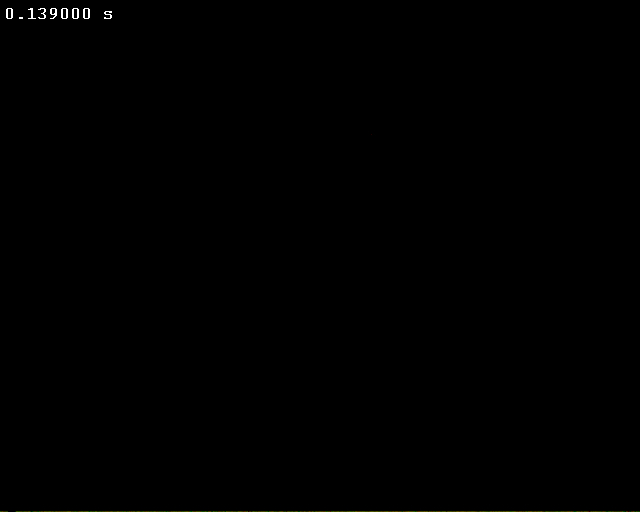

Supplement: S3 File — (ZIP) [file pone.0237709.s003.zip › PEDOT Electrode Recording/Position000139.tif]

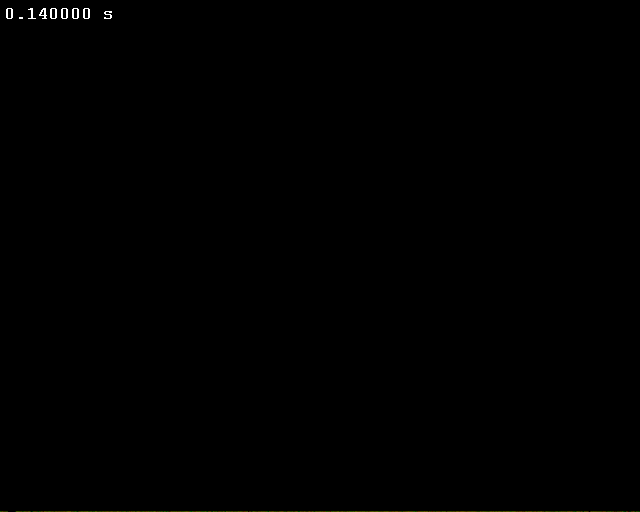

Supplement: S3 File — (ZIP) [file pone.0237709.s003.zip › PEDOT Electrode Recording/Position000140.tif]

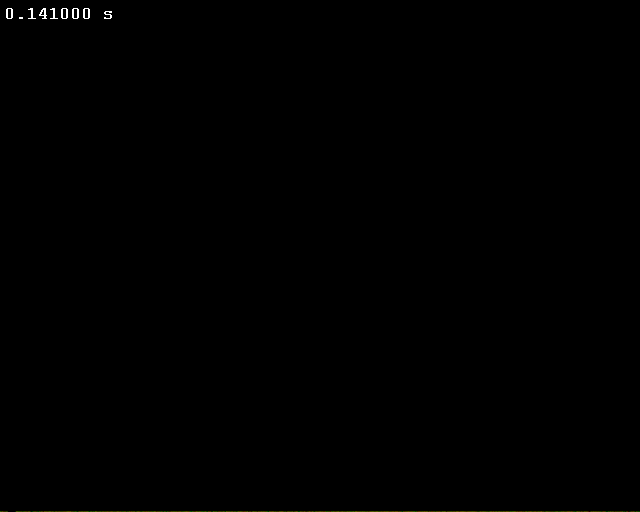

Supplement: S3 File — (ZIP) [file pone.0237709.s003.zip › PEDOT Electrode Recording/Position000141.tif]

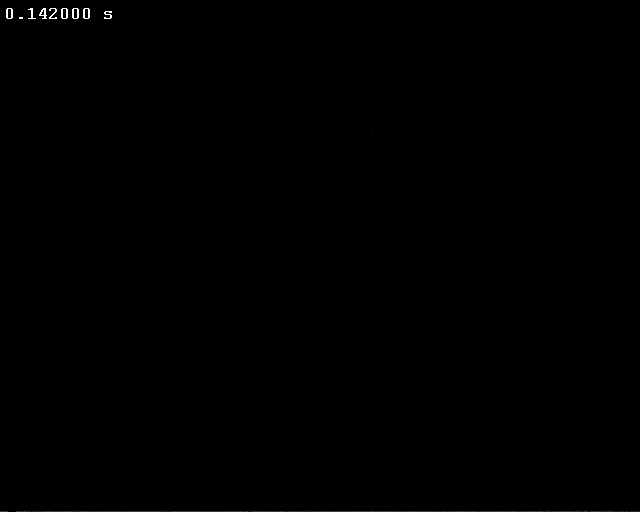

Supplement: S3 File — (ZIP) [file pone.0237709.s003.zip › PEDOT Electrode Recording/Position000142.tif]

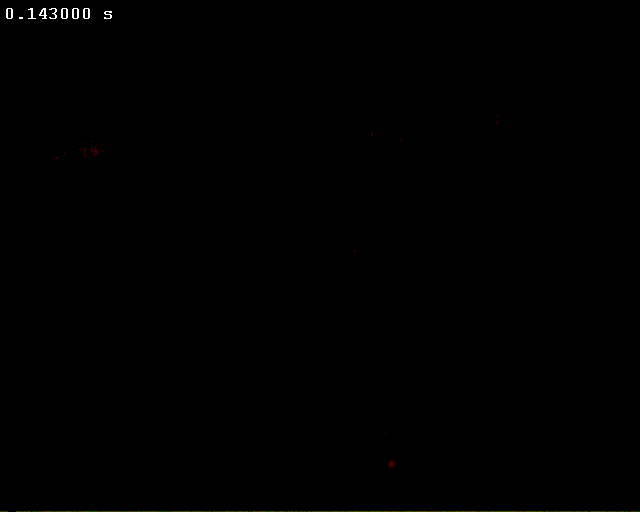

Supplement: S3 File — (ZIP) [file pone.0237709.s003.zip › PEDOT Electrode Recording/Position000143.tif]

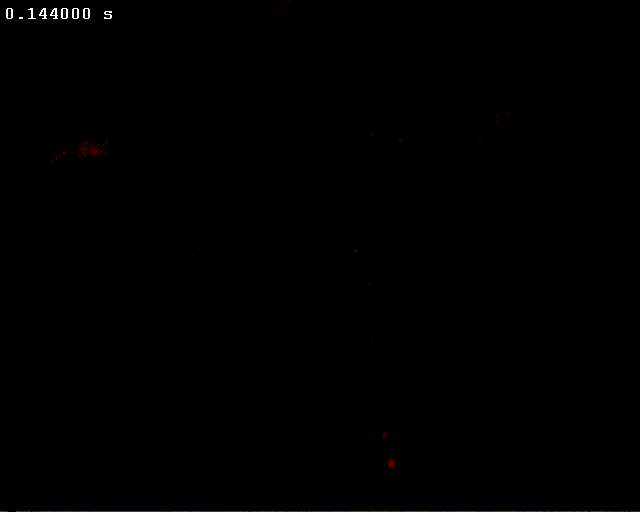

Supplement: S3 File — (ZIP) [file pone.0237709.s003.zip › PEDOT Electrode Recording/Position000144.tif]

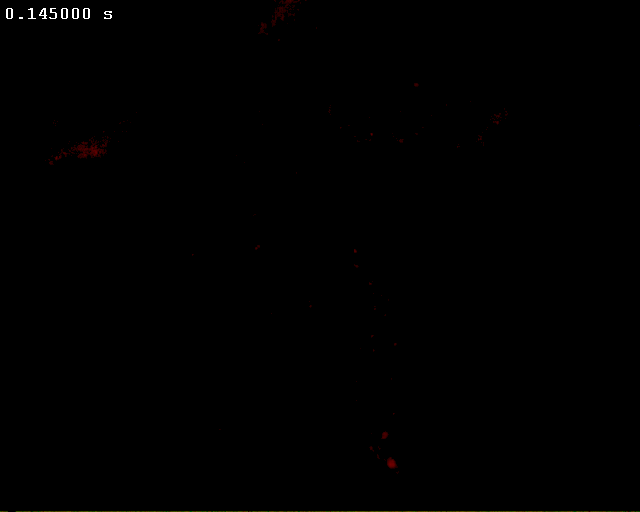

Supplement: S3 File — (ZIP) [file pone.0237709.s003.zip › PEDOT Electrode Recording/Position000145.tif]

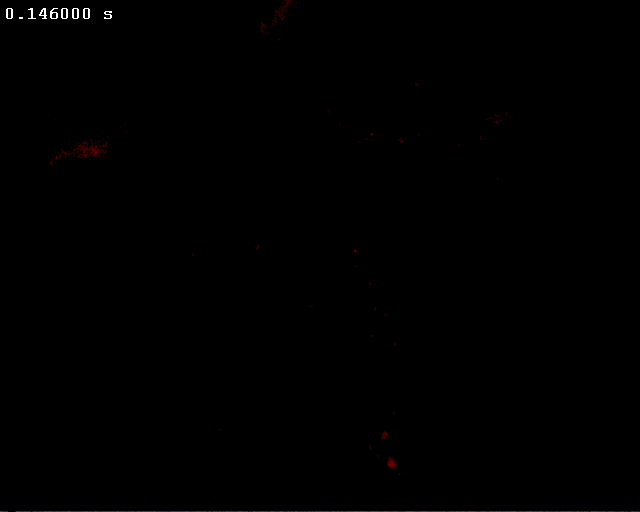

Supplement: S3 File — (ZIP) [file pone.0237709.s003.zip › PEDOT Electrode Recording/Position000146.tif]

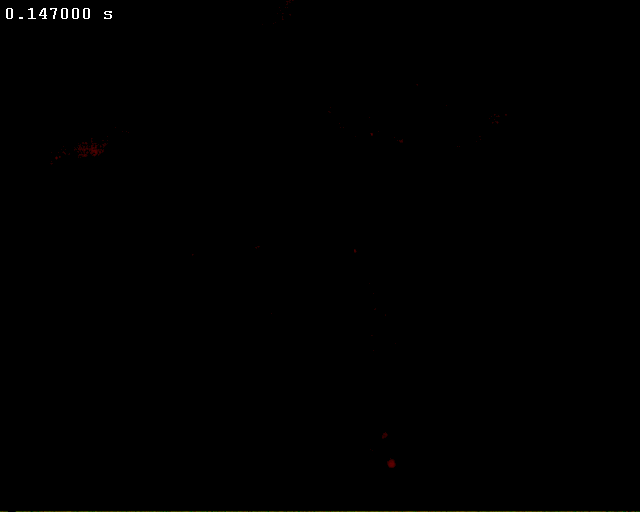

Supplement: S3 File — (ZIP) [file pone.0237709.s003.zip › PEDOT Electrode Recording/Position000147.tif]

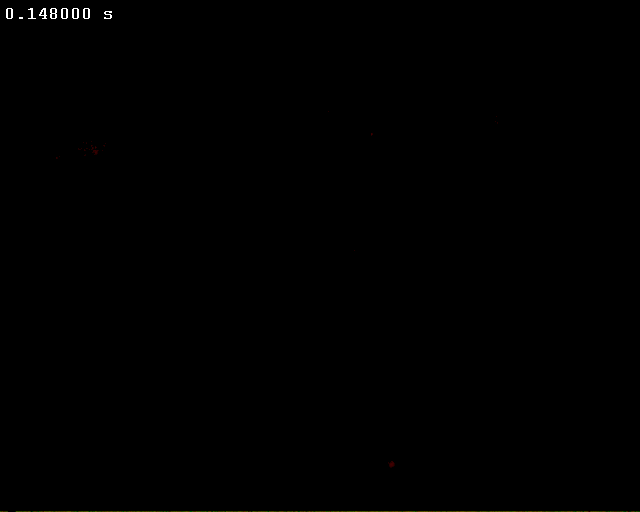

Supplement: S3 File — (ZIP) [file pone.0237709.s003.zip › PEDOT Electrode Recording/Position000148.tif]

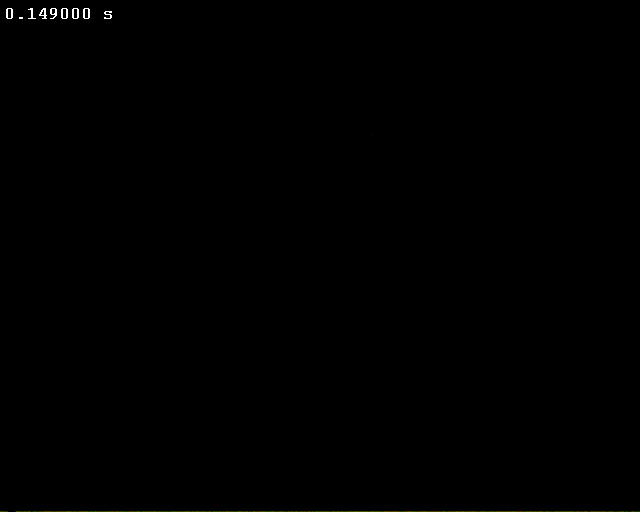

Supplement: S3 File — (ZIP) [file pone.0237709.s003.zip › PEDOT Electrode Recording/Position000149.tif]

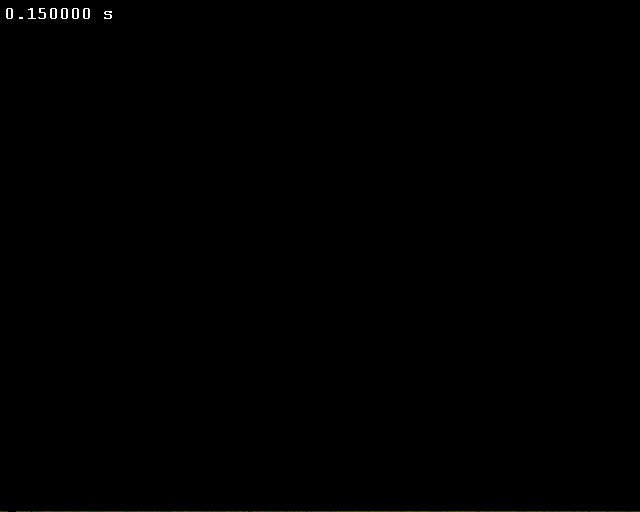

Supplement: S3 File — (ZIP) [file pone.0237709.s003.zip › PEDOT Electrode Recording/Position000150.tif]

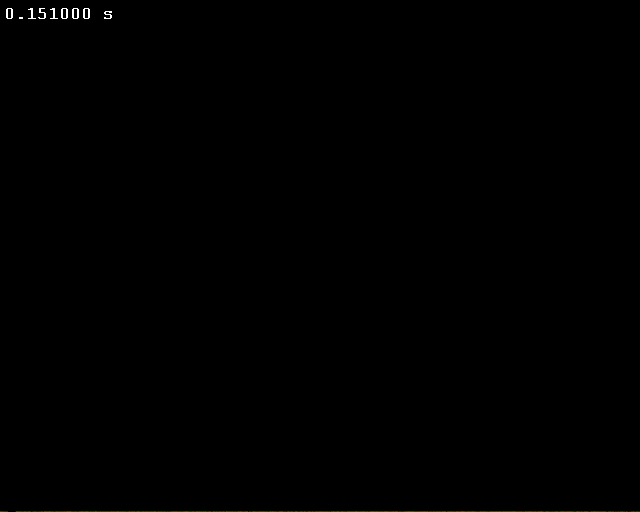

Supplement: S3 File — (ZIP) [file pone.0237709.s003.zip › PEDOT Electrode Recording/Position000151.tif]

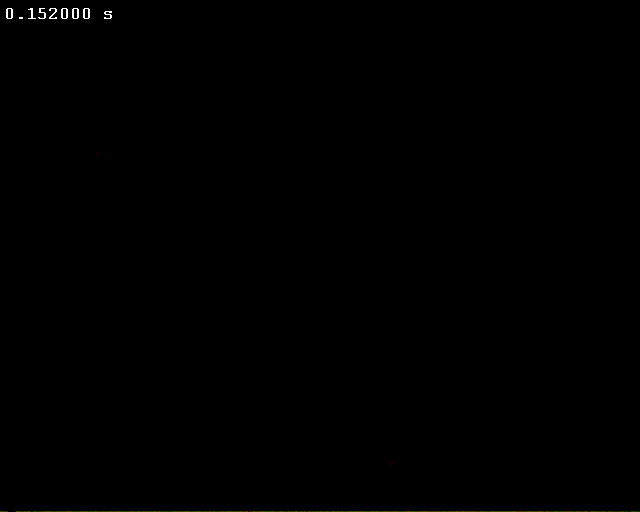

Supplement: S3 File — (ZIP) [file pone.0237709.s003.zip › PEDOT Electrode Recording/Position000152.tif]

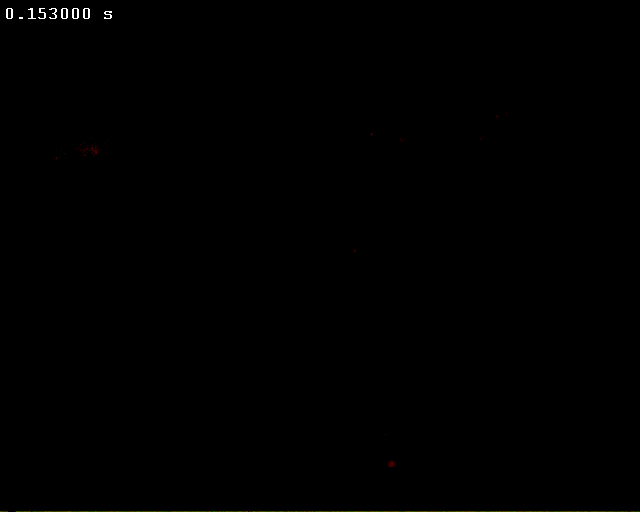

Supplement: S3 File — (ZIP) [file pone.0237709.s003.zip › PEDOT Electrode Recording/Position000153.tif]

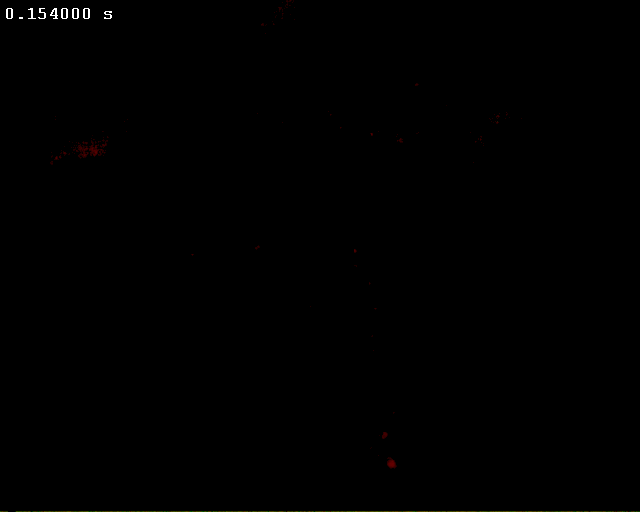

Supplement: S3 File — (ZIP) [file pone.0237709.s003.zip › PEDOT Electrode Recording/Position000154.tif]

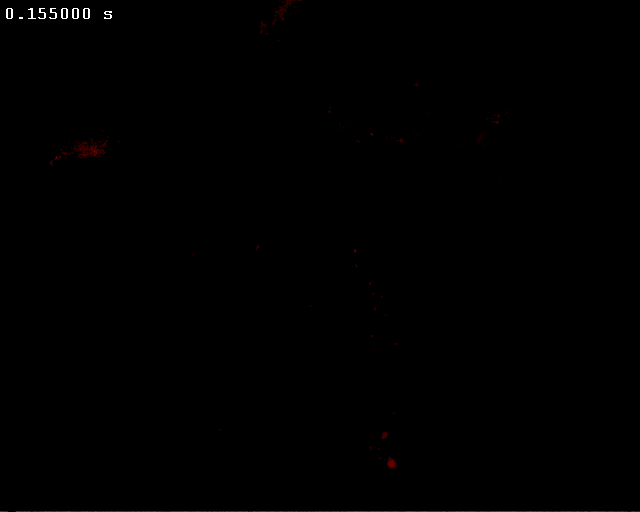

Supplement: S3 File — (ZIP) [file pone.0237709.s003.zip › PEDOT Electrode Recording/Position000155.tif]

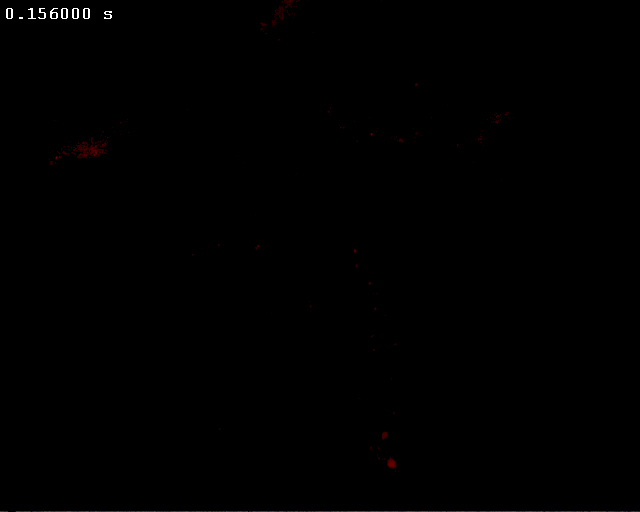

Supplement: S3 File — (ZIP) [file pone.0237709.s003.zip › PEDOT Electrode Recording/Position000156.tif]

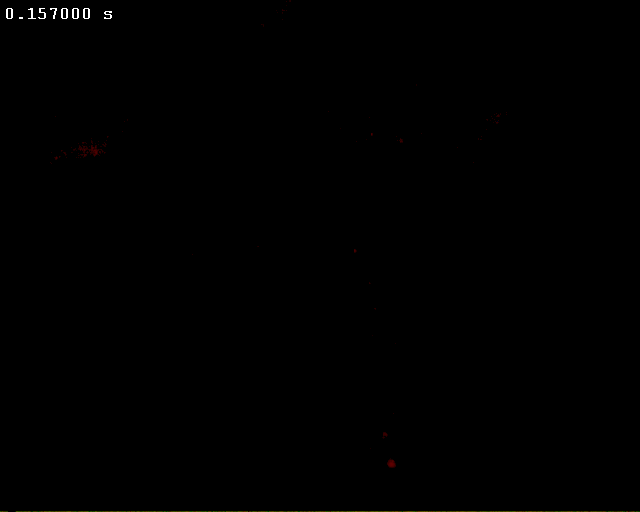

Supplement: S3 File — (ZIP) [file pone.0237709.s003.zip › PEDOT Electrode Recording/Position000157.tif]

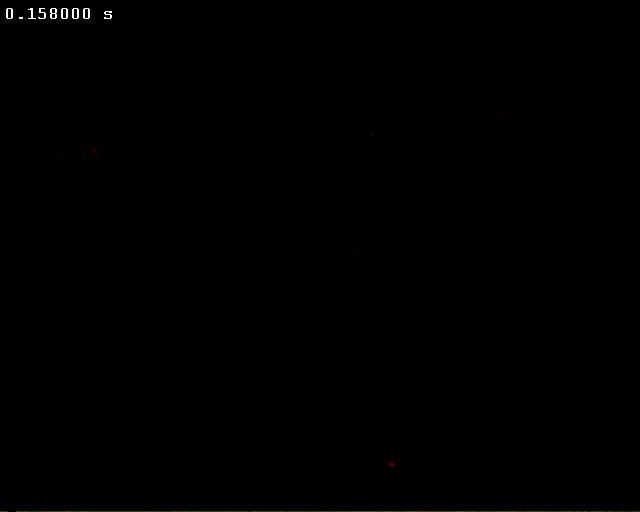

Supplement: S3 File — (ZIP) [file pone.0237709.s003.zip › PEDOT Electrode Recording/Position000158.tif]

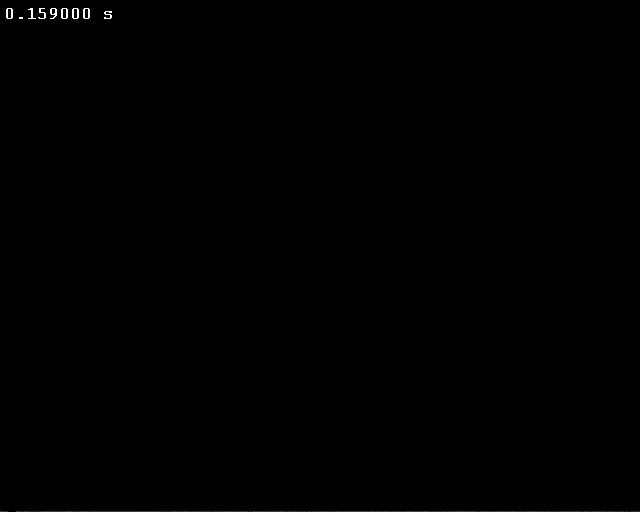

Supplement: S3 File — (ZIP) [file pone.0237709.s003.zip › PEDOT Electrode Recording/Position000159.tif]

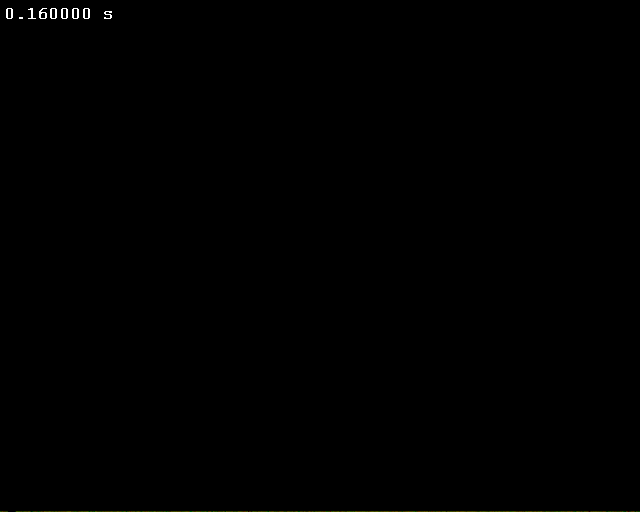

Supplement: S3 File — (ZIP) [file pone.0237709.s003.zip › PEDOT Electrode Recording/Position000160.tif]

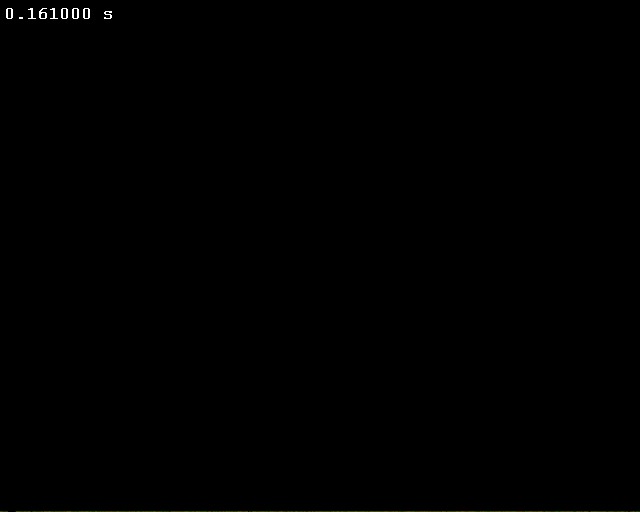

Supplement: S3 File — (ZIP) [file pone.0237709.s003.zip › PEDOT Electrode Recording/Position000161.tif]

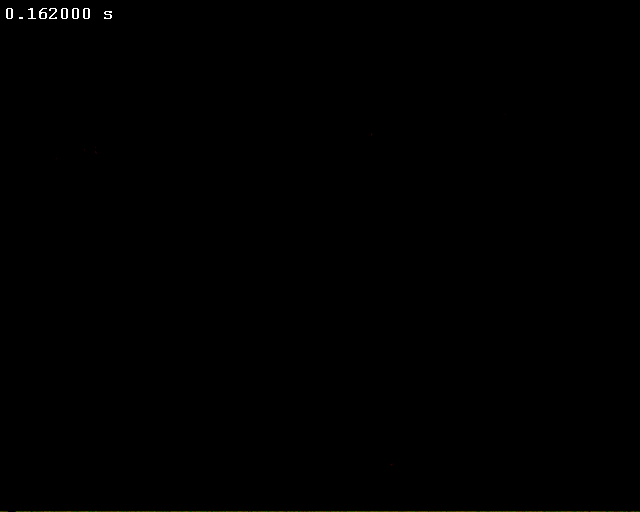

Supplement: S3 File — (ZIP) [file pone.0237709.s003.zip › PEDOT Electrode Recording/Position000162.tif]

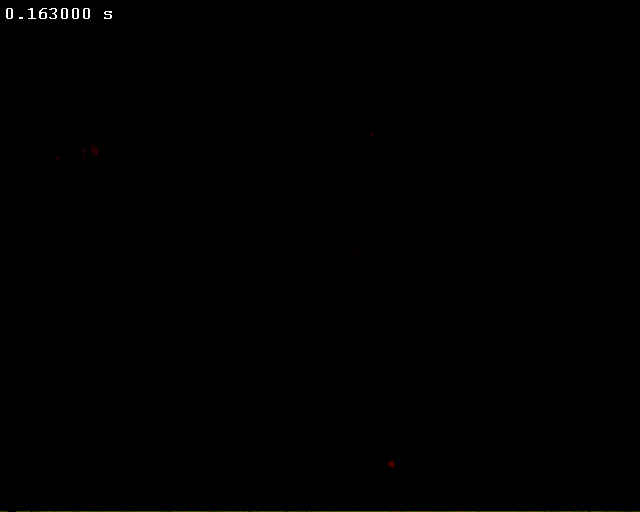

Supplement: S3 File — (ZIP) [file pone.0237709.s003.zip › PEDOT Electrode Recording/Position000163.tif]

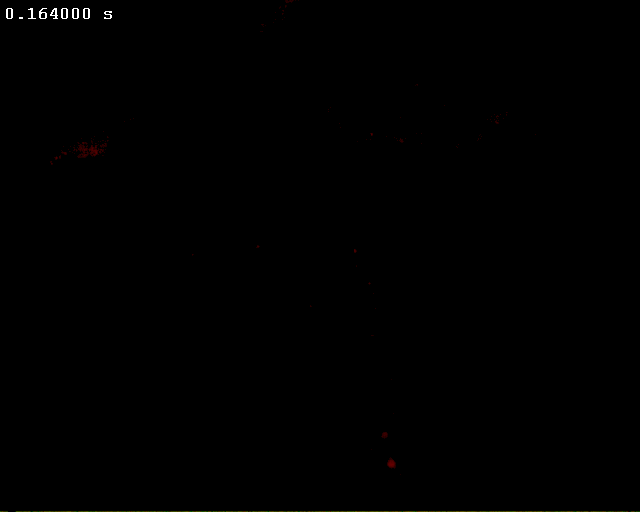

Supplement: S3 File — (ZIP) [file pone.0237709.s003.zip › PEDOT Electrode Recording/Position000164.tif]

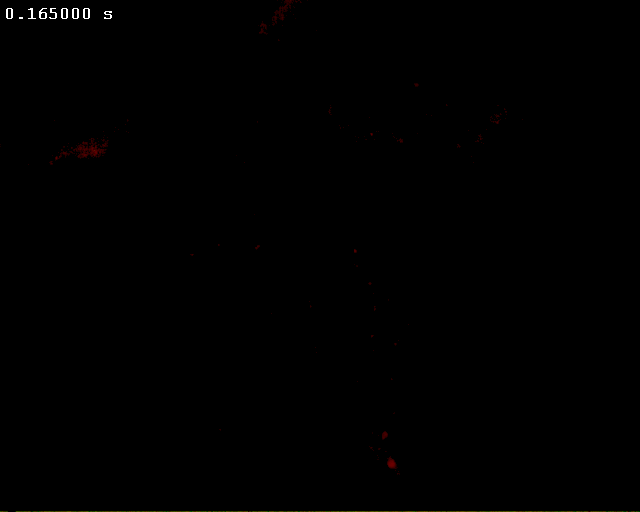

Supplement: S3 File — (ZIP) [file pone.0237709.s003.zip › PEDOT Electrode Recording/Position000165.tif]

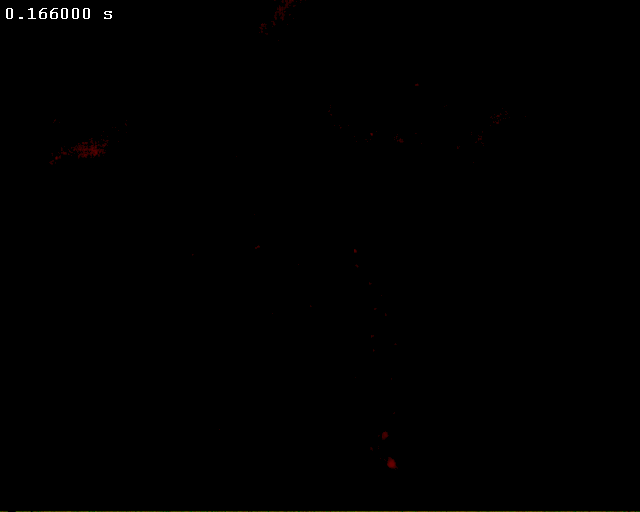

Supplement: S3 File — (ZIP) [file pone.0237709.s003.zip › PEDOT Electrode Recording/Position000166.tif]

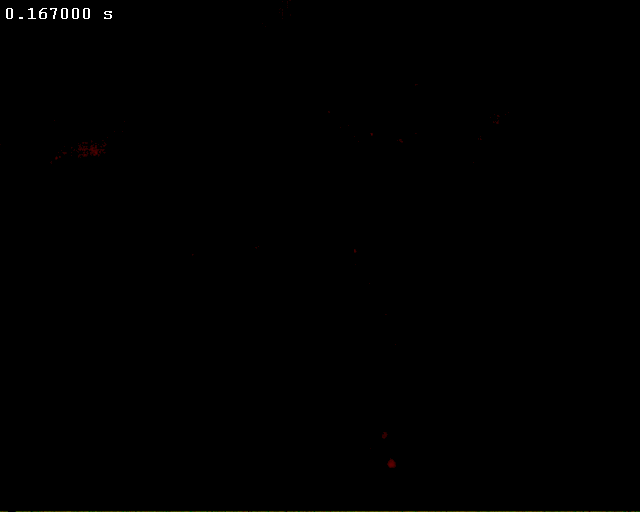

Supplement: S3 File — (ZIP) [file pone.0237709.s003.zip › PEDOT Electrode Recording/Position000167.tif]

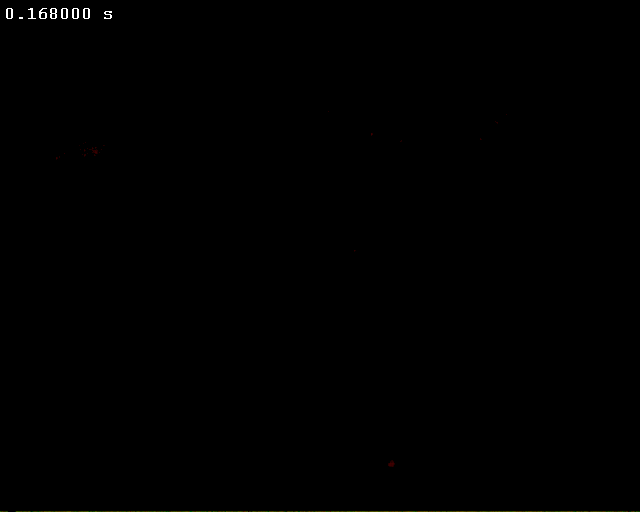

Supplement: S3 File — (ZIP) [file pone.0237709.s003.zip › PEDOT Electrode Recording/Position000168.tif]

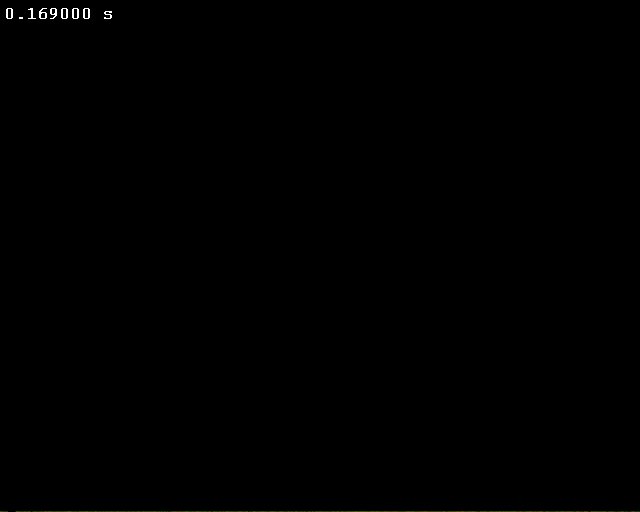

Supplement: S3 File — (ZIP) [file pone.0237709.s003.zip › PEDOT Electrode Recording/Position000169.tif]

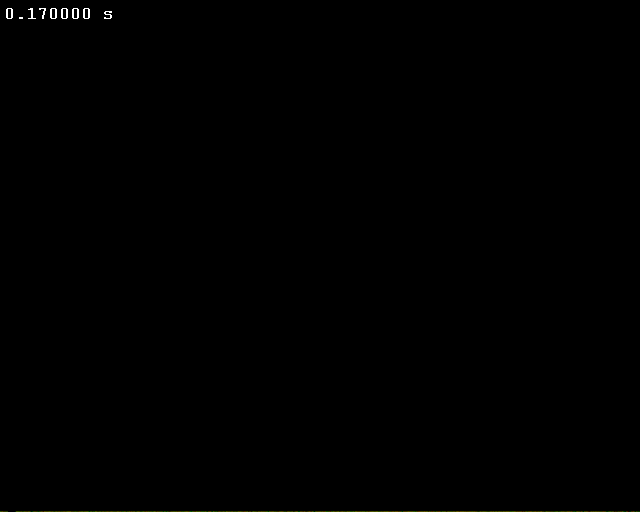

Supplement: S3 File — (ZIP) [file pone.0237709.s003.zip › PEDOT Electrode Recording/Position000170.tif]

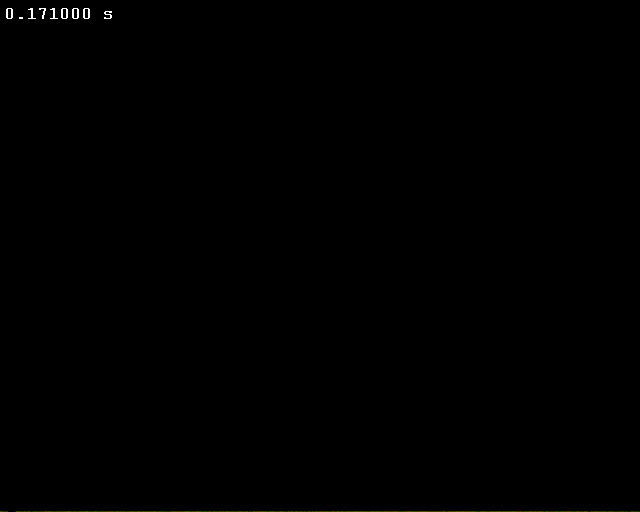

Supplement: S3 File — (ZIP) [file pone.0237709.s003.zip › PEDOT Electrode Recording/Position000171.tif]

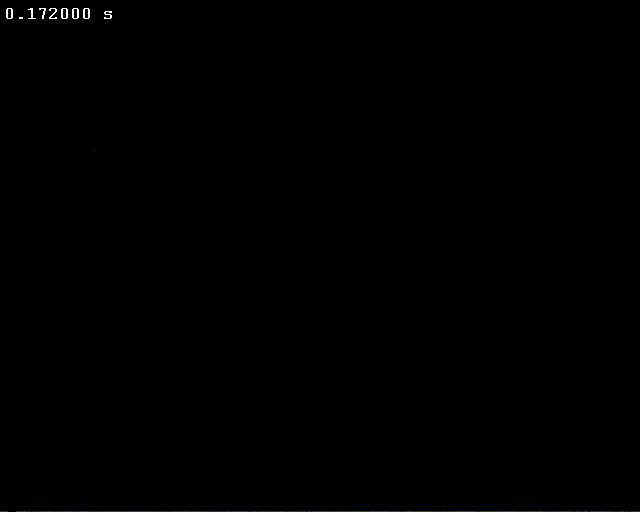

Supplement: S3 File — (ZIP) [file pone.0237709.s003.zip › PEDOT Electrode Recording/Position000172.tif]

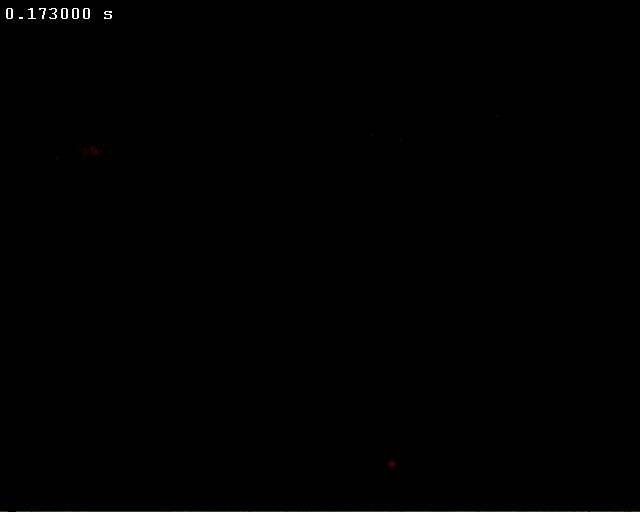

Supplement: S3 File — (ZIP) [file pone.0237709.s003.zip › PEDOT Electrode Recording/Position000173.tif]

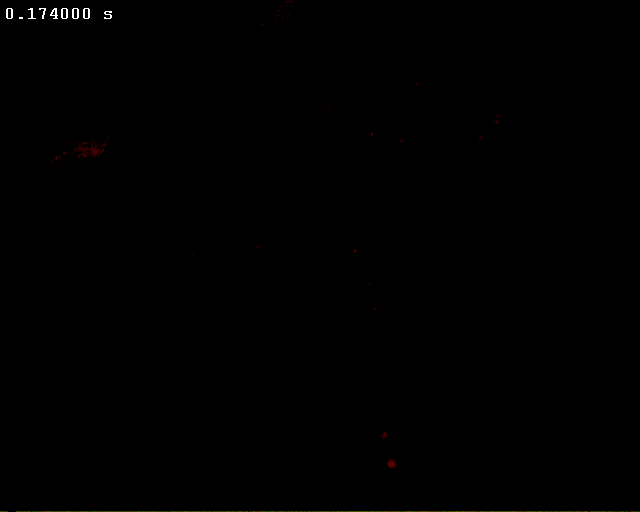

Supplement: S3 File — (ZIP) [file pone.0237709.s003.zip › PEDOT Electrode Recording/Position000174.tif]

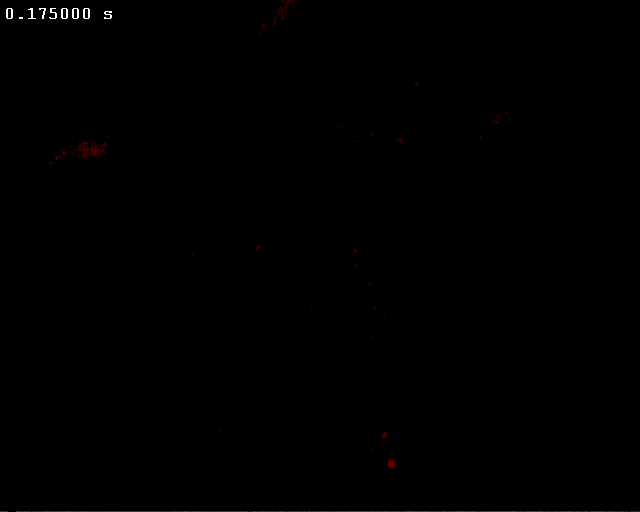

Supplement: S3 File — (ZIP) [file pone.0237709.s003.zip › PEDOT Electrode Recording/Position000175.tif]

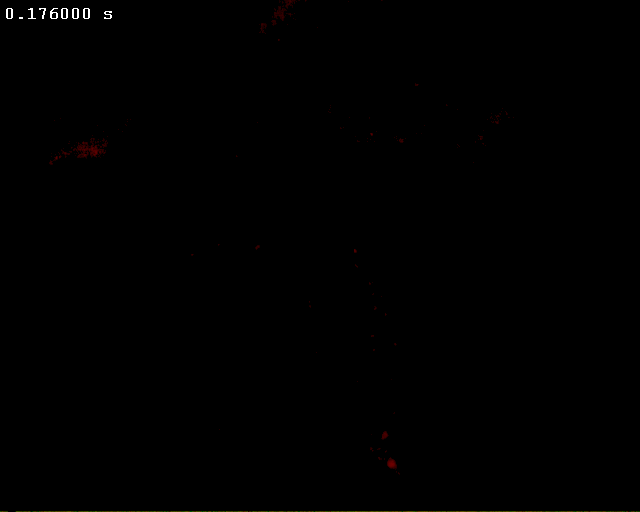

Supplement: S3 File — (ZIP) [file pone.0237709.s003.zip › PEDOT Electrode Recording/Position000176.tif]

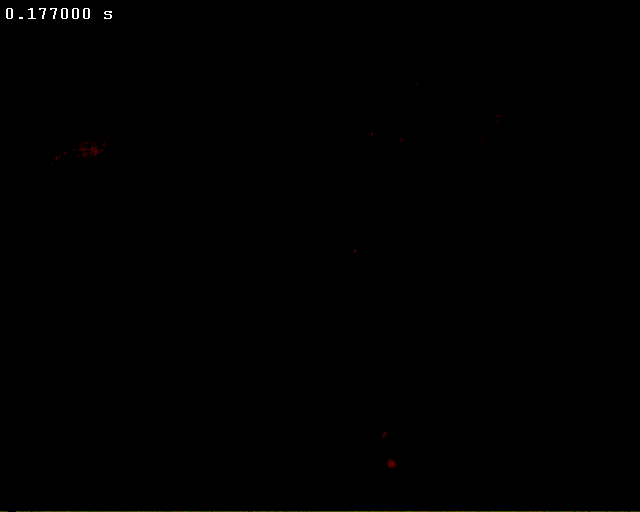

Supplement: S3 File — (ZIP) [file pone.0237709.s003.zip › PEDOT Electrode Recording/Position000177.tif]

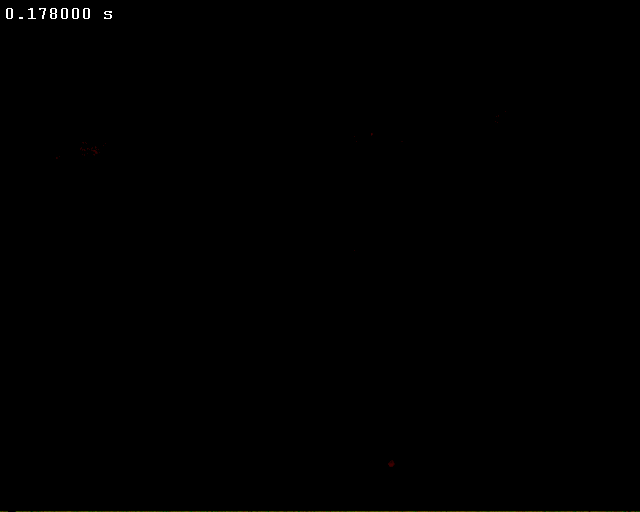

Supplement: S3 File — (ZIP) [file pone.0237709.s003.zip › PEDOT Electrode Recording/Position000178.tif]

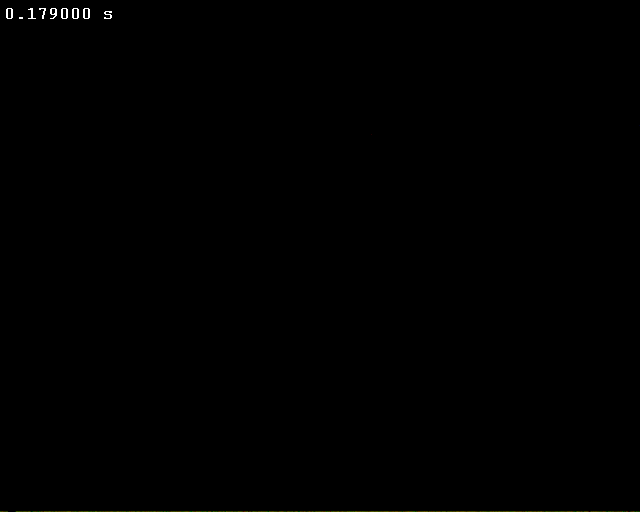

Supplement: S3 File — (ZIP) [file pone.0237709.s003.zip › PEDOT Electrode Recording/Position000179.tif]

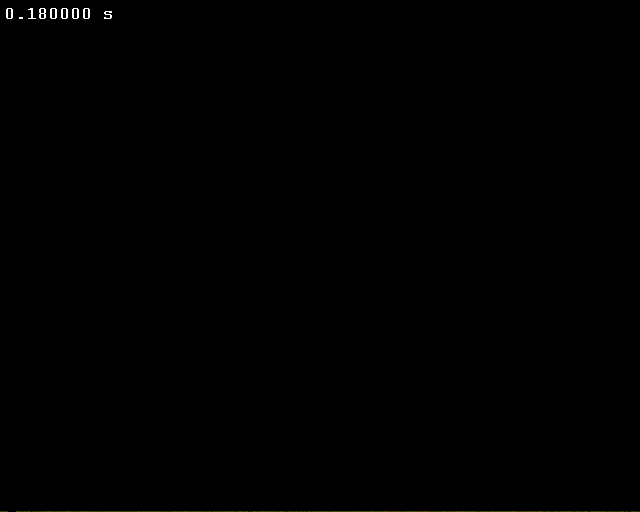

Supplement: S3 File — (ZIP) [file pone.0237709.s003.zip › PEDOT Electrode Recording/Position000180.tif]

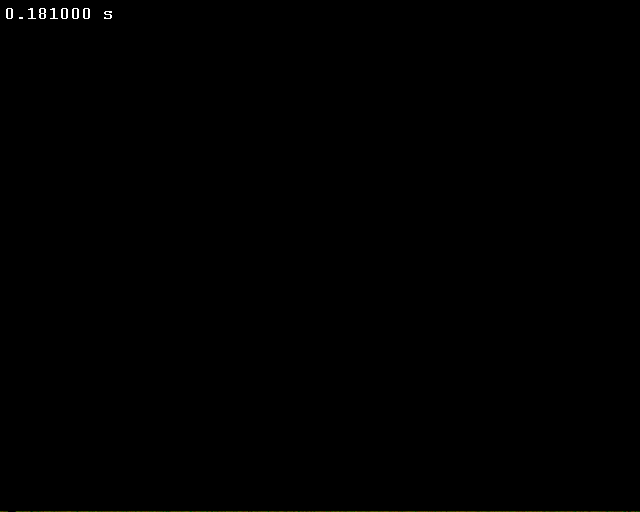

Supplement: S3 File — (ZIP) [file pone.0237709.s003.zip › PEDOT Electrode Recording/Position000181.tif]

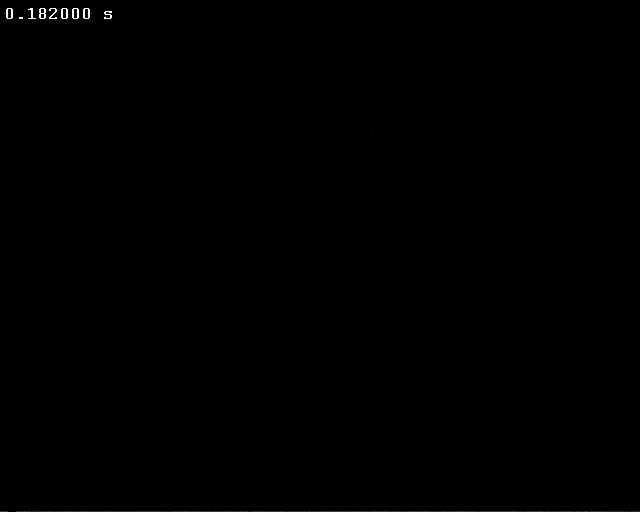

Supplement: S3 File — (ZIP) [file pone.0237709.s003.zip › PEDOT Electrode Recording/Position000182.tif]

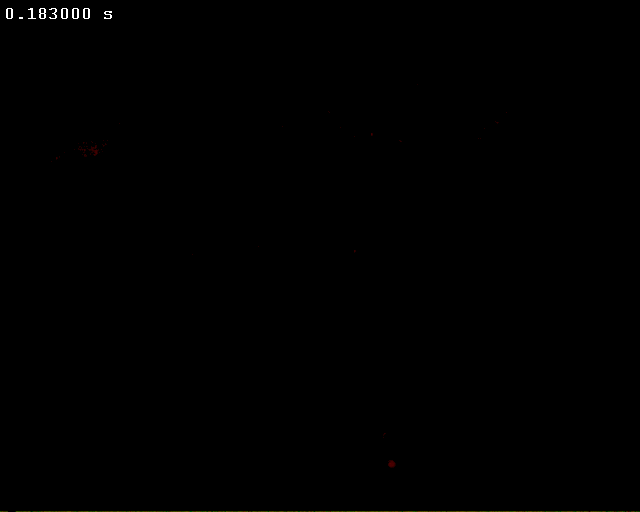

Supplement: S3 File — (ZIP) [file pone.0237709.s003.zip › PEDOT Electrode Recording/Position000183.tif]

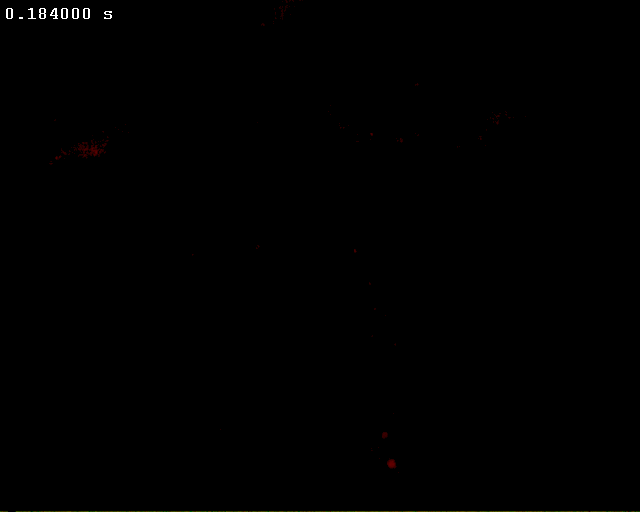

Supplement: S3 File — (ZIP) [file pone.0237709.s003.zip › PEDOT Electrode Recording/Position000184.tif]

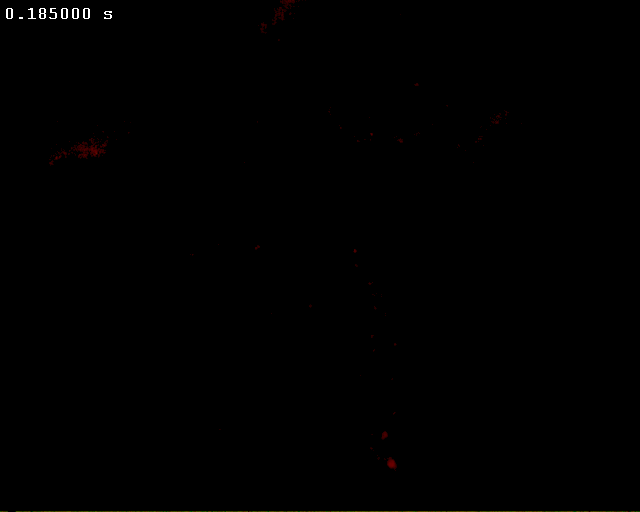

Supplement: S3 File — (ZIP) [file pone.0237709.s003.zip › PEDOT Electrode Recording/Position000185.tif]

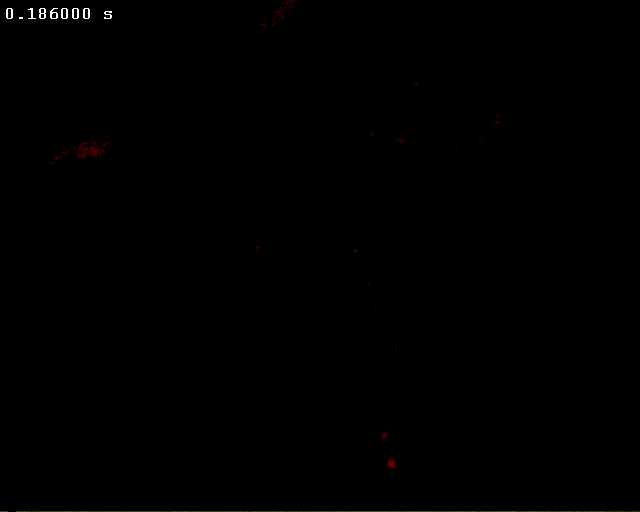

Supplement: S3 File — (ZIP) [file pone.0237709.s003.zip › PEDOT Electrode Recording/Position000186.tif]

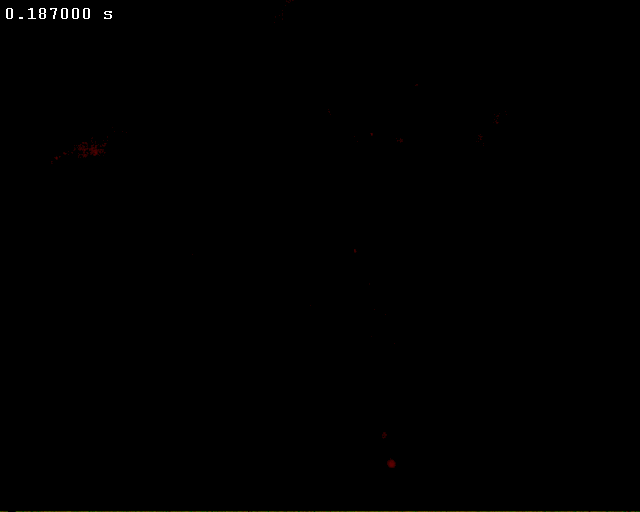

Supplement: S3 File — (ZIP) [file pone.0237709.s003.zip › PEDOT Electrode Recording/Position000187.tif]

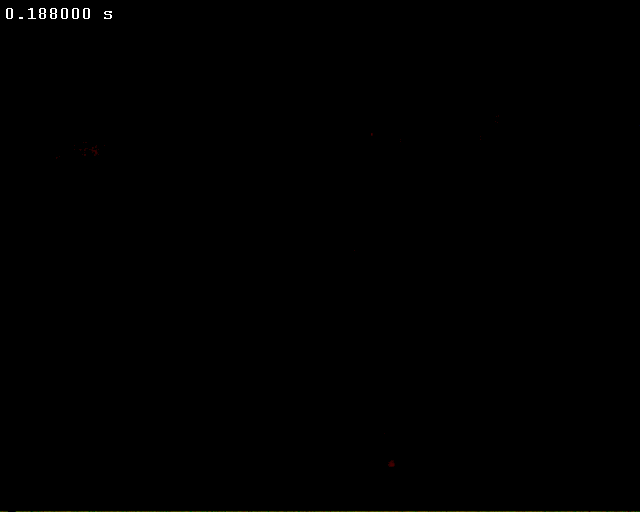

Supplement: S3 File — (ZIP) [file pone.0237709.s003.zip › PEDOT Electrode Recording/Position000188.tif]

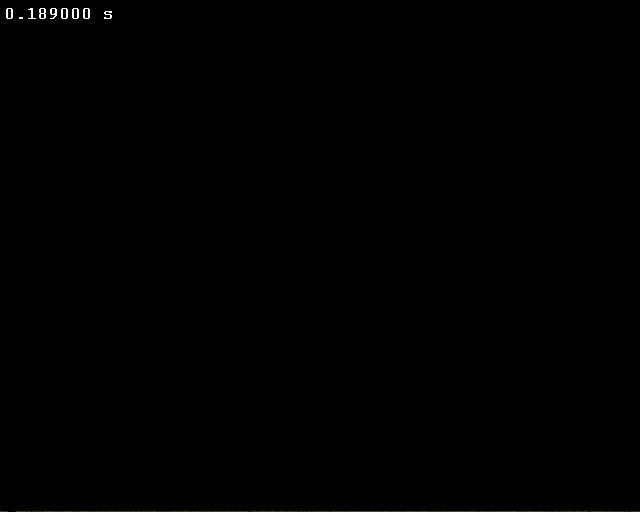

Supplement: S3 File — (ZIP) [file pone.0237709.s003.zip › PEDOT Electrode Recording/Position000189.tif]

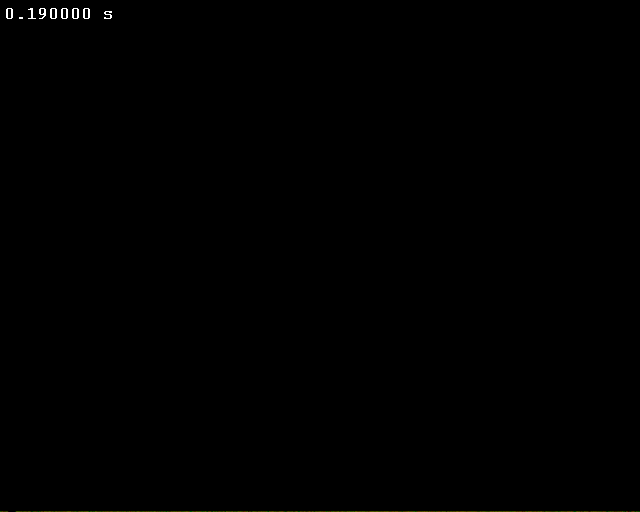

Supplement: S3 File — (ZIP) [file pone.0237709.s003.zip › PEDOT Electrode Recording/Position000190.tif]

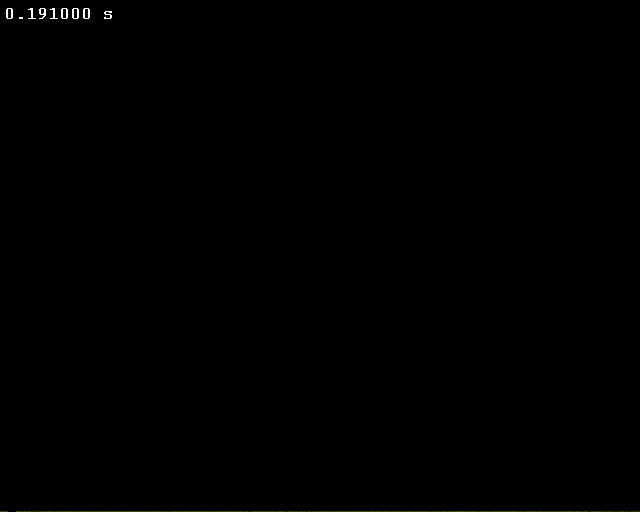

Supplement: S3 File — (ZIP) [file pone.0237709.s003.zip › PEDOT Electrode Recording/Position000191.tif]

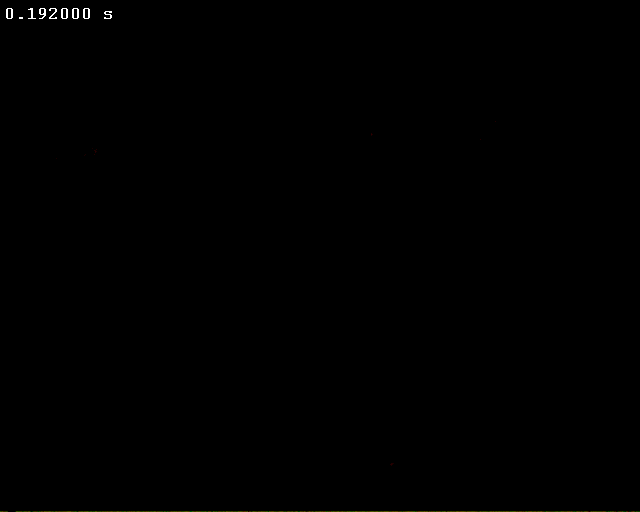

Supplement: S3 File — (ZIP) [file pone.0237709.s003.zip › PEDOT Electrode Recording/Position000192.tif]

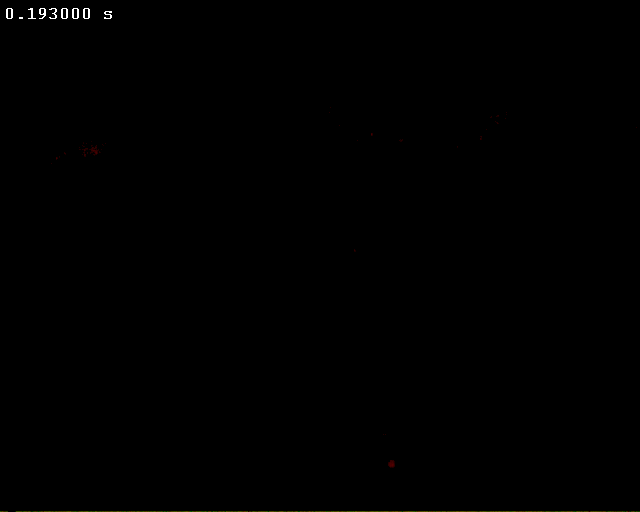

Supplement: S3 File — (ZIP) [file pone.0237709.s003.zip › PEDOT Electrode Recording/Position000193.tif]

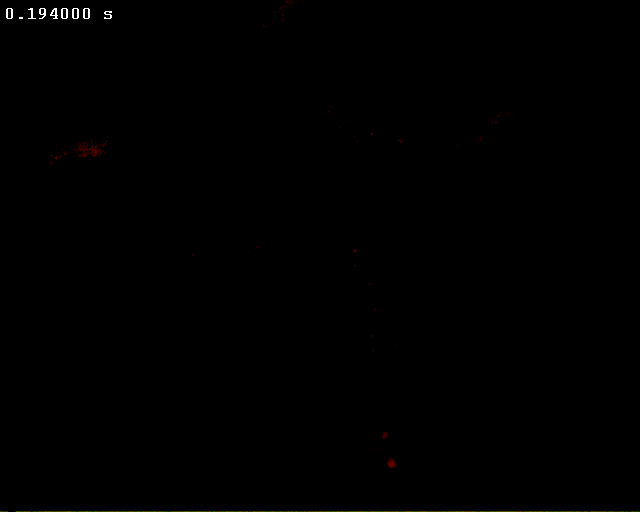

Supplement: S3 File — (ZIP) [file pone.0237709.s003.zip › PEDOT Electrode Recording/Position000194.tif]

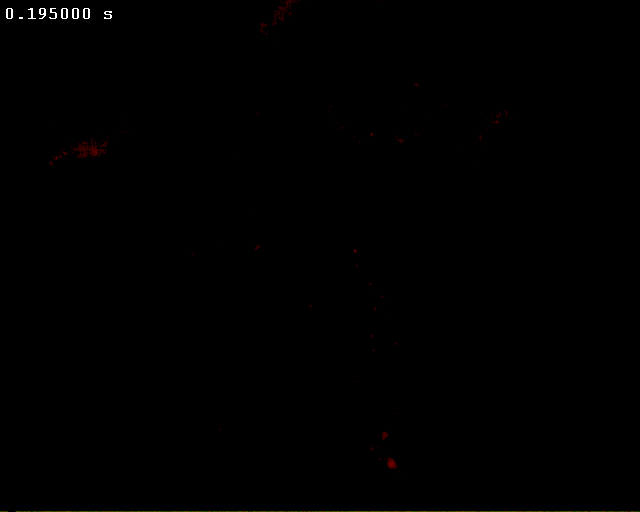

Supplement: S3 File — (ZIP) [file pone.0237709.s003.zip › PEDOT Electrode Recording/Position000195.tif]
